# ctDNA guided management of POLE mutant GI malignancies promotes exceptional responses and prolonged survival to immunotherapy

**DOI:** 10.3389/fimmu.2026.1738295

**Published:** 2026-02-17

**Authors:** Umair Mahmood, Chetan Bhan, Charles Imber, Jamie Murphy, Krish Menon, Tara D. Barwick, Zahir Amin, Khurum Khan

**Affiliations:** 1 Department of Gastrointestinal Oncology, University College London Hospitals NHS Foundation Trust, London, United Kingdom; 2 Cleveland Clinic London, London, United Kingdom; 3 Imperial College Healthcare NHS Trust, London, United Kingdom; 4 The London Clinic, London, United Kingdom; 5 Department of Radiology, University College Hospital NHS Foundation Trust, London, United Kingdom; 6 HCA Healthcare UK, London, United Kingdom

**Keywords:** colorectal cancer, ctDNA, gall bladder cancer, immunotherapy, pole

## Abstract

Patients with unresectable Gastrointestinal cancers (GI) including advanced colorectal cancer (CRC) and gall bladder cancer (GBC) have limited treatment options. Treatment with chemotherapy is associated with limited success resulting from therapeutic resistance. Immune checkpoint inhibitors are effective for a subset of metastatic CRC (5% with MSI-H status) and up to 30% of patients with GBC. Therefore, there is an urgent unmet need to discover new predictive biomarkers to aid patient selection for ICIs with a demonstrated clinical value in a challenging patient population. We highlight the utility of a liquid biopsy approach to aid selection of GI cancer patients harbouring rare *POLE* mutations for immunotherapy, leading to complete metabolic response in addition to radiologic responses and extended survival in all three patients. This study advocates for specialised multi-disciplinary teams performing shared clinical decision making to advance personalised care and improve outcomes of a subset of GI cancer patients with a poor prognosis.

## Introduction

Patients with GI cancers represent a clinical population with poor outcomes after conventional treatments and represent an active avenue of investigation to meet a major medical need. Immune checkpoint inhibitors (ICIs) targeting the programmed death-1 (PD-1) protein have shown significant efficacy and are now the treatment of choice for patients with mismatch repair-deficient (dMMR) metastatic colorectal cancer (1) and offer modest benefit in patients with biliary tract cancers when combined with chemotherapy. However, dMMR accounts for only a small subset of cases and despite advancements in systemic treatments such as immunotherapy, a paucity of additional validated predictive biomarkers in these GI malignancies can limit the potential use of ICIs in these disease settings.

The advent of liquid biopsies offers an emerging platform for molecularly guided treatment stratification to identify candidates well suited for ICIs. In this instance, DNA polymerase epsilon (*POLE*) could be utilised which encodes the catalytic subunit of the DNA polymerase epsilon enzyme and plays a critical role in DNA replication and repair (2). Mutations in the exonuclease domain of *POLE* (*POLE* EDM) are rare but have been identified across various malignancies, with the highest frequencies observed in endometrial cancer (6%–12%) (3) and CRC (1%–2%) (4), and a few instances in gallbladder cancer (5, 6). *POLE* EDM in endometrial cancer is associated with a strong intratumoural T-cell response, likely due to a high neoantigen load driven by an ultra-mutated tumour phenotype, contributing to favourable clinical outcomes (7). Similarly, in stage II colorectal cancer, somatic *POLE* EDM are linked to elevated intratumoural immune activity, driven partly by an increased tumour infiltration of CD8+ lymphocytes and CD8A expression (8). However, given the scarcity of data regarding *POLE* mutant clinical population, there is a notable absence of a standardised therapeutic protocol tailored to these patients. Additionally, there is no clear guidance on the duration of ICIs where data is extrapolated from dMMR CRC patients, which often warrants an assessment by clinicians within the appropriate clinical context. There is also a paucity of data to guide whether surgery should be offered in the event of complete metabolic response (CMR).

Our study provides a foundation to address these questions via successful treatment of *POLE* mutant patients achieving CMR. In this study, we analysed patients with gastrointestinal cancer (GI) including colorectal or gallbladder cancer who carried a *POLE* EDM and were managed with ICIs after diagnosis. Clinical management, radiologic outcomes and post-operative pathological evaluations were documented and reviewed. Additionally, gene mutation profiles and tumour mutation burden (TMB) were examined in these *POLE* EDM cases along with molecular characteristics, including *POLE* non-EDM and microsatellite stability (MSS) profiles.

## Methods

### Patient selection

We identified three adult gastrointestinal cancer patients harbouring a *POLE* EDM and treated with an ICI, with or without additional systemic agents. We abstracted data from patient medical records including clinician’s notes, radiology, operative and pathology reports as well as clinical reports regarding next-generation sequencing. Radiological and metabolic response was assessed using the immune Response Evaluation Criteria in Solid Tumours (iRECIST) (9) and immunotherapy PET Response Criteria in Solid Tumours (iPERCIST) (10). Assessment of survival outcomes comprised of progression-free survival (PFS) and overall survival (OS) after commencing ICI.

### Next-generation sequencing

Prior to commencing systemic treatment, all patients underwent the FoundationOne^®^Liquid CDx test, a next generation sequencing (NGS) assay which uses a patient’s plasma sample to identify clinically relevant genomic alterations in circulating cell-free DNA (cfDNA). An additional FoundationOne^®^Liquid CDx test was performed for one patient after completing systemic treatment. An additional patient underwent a Guardant Reveal™ test towards completion of immunotherapy.

Patients were subjected to a wide panel genomic sequencing for simultaneous detection of micro-satellite instability (MSI) status and genetic aberrations in 324 cancer related genes, including *KRAS, NRAS, BRAF, BRCA1/2, HER2, TP53, POLE* and other genes related to tumour development and carcinogenesis. TMB was also reported and was defined as the number of somatic non‐synonymous mutations per megabase of genome examined. Tumour fraction was assessed as an estimate of the percentage of circulating-tumour DNA (ctDNA) present in a cfDNA sample based on observed aneuploid instability. Additional details regarding technical specifications of molecular assays are reported under **Supplementary File 1**.

## Results

### Clinical characteristics and genomic profiles of POLE mutant patients

**Figure 1:**
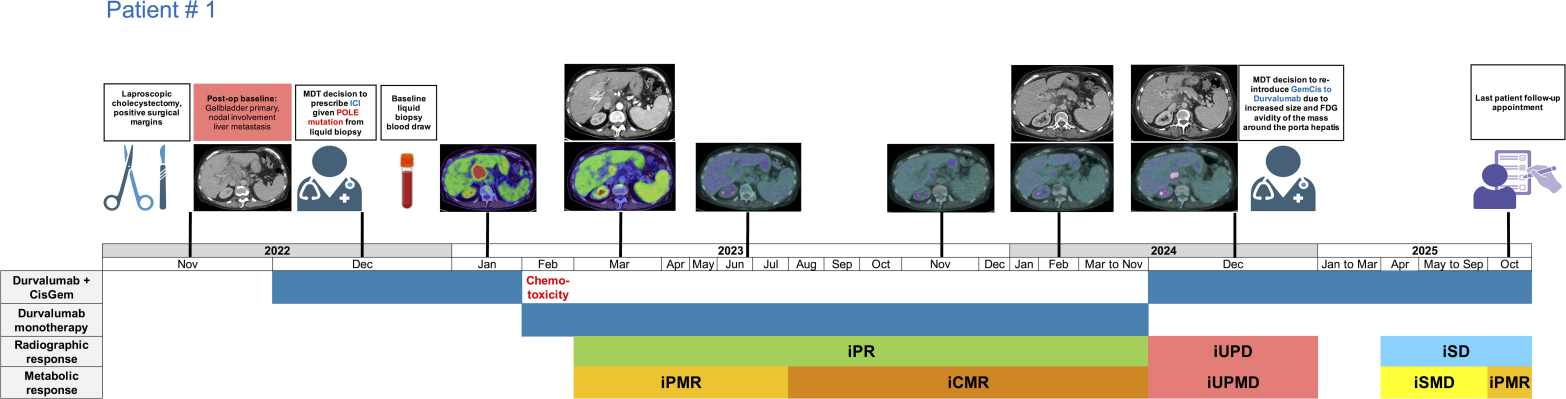
Treatment timeline of patient # 1 receiving long-term durvalumab with and without CisGem for the management of gallbladder adenocarcinoma. CisGem, cisplatin and gemcitabine, iCMR, immunotherapy complete metabolic response; iPR, immunotherapy partial response; iPMR, immunotherapy partial metabolic response; iSD, immunotherapy stable disease; iSMD, immunotherapy stable metabolic disease, iUPD; unconfirmed progressive disease; iUPMD, unconfirmed progressive metabolic disease.

Patient # 1 is a 71-year-old female who was diagnosed with unresectable, moderate to poorly differentiated gallbladder adenocarcinoma in November 2022 (**Figure 1**, **Table 1**). The patient had no prior family history of cancer. She presented with extensive involvement of the porta hepatis, hepatic artery, portal vein, and liver metastases. Molecular profiling of her baseline blood test revealed a *POLE* R150* mutation. This variant is a truncating nonsense mutation predicted to result in loss of function via premature protein truncation or nonsense-mediated decay. According to ACMG/AMP criteria (11), this variant meets PVS1 (predicted null variant in a gene where loss of function is a known disease mechanism) and PM2 (absence from population databases such as gnomAD), supporting classification as a likely pathogenic loss-of-function variant at the gene level. Importantly, this variant is located outside the POLE exonuclease proofreading domain (amino acids ~268–471) and therefore represents a non-EDM. Comprehensive mutational signature analysis was not available. Additionally, the patient also harboured *DNMT3A* R882H (Variant Allele Frequency percentage (VAF): 4.3%) and a *TP53* splice site 559 + 1G>A aberration (VAF: 8.3%) (**Supplementary Table 1**). The subject was microsatellite stable (MSS) and her TMB was reported as 4 Muts/Mb with an elevated tumour fraction (13%).

**Table 1:** Clinicopathological features, treatment, and outcomes of three gastrointestinal cancer patients harbouring *POLE* mutations.

	Patient # 1	Patient # 2	Patient # 3
Baseline characteristics			
Age	71	45	30
Sex	Female	Male	Male
Cancer type	Gallbladder adenocarcinoma	Colorectal adenocarcinoma	Rectal cancer
POLE variants	POLE R150*	*POLE* P286R, *POLE* L1235I	*POLE* P286R (VAF: 53.0%), *POLE* L1441R, *POLE* R1382C, *POLE* R1826W, *POLE* R2131C
Other major genomic variants	*DNMT3A* R882H (VAF: 4.3%), *TP53* splice site 559 + 1G>A aberration (VAF: 8.3%)	*KRAS* G13D (VAF: 0.41%), *BRCA2* E34* (VAF: 0.19%), *NF1* splice site 1063-1G>T (VAF: 0.73%)	*ATM* R35* (VAF: 41.6%), *BRCA1* E1353*(VAF: 43.8%), *BRCA2* E1593* (VAF: 0.21%), *BRCA2* E510* (VAF: 0.39%), *BRCA2* E2599* (VAF: 0.44%), *PIK3CA* E542A (VAF: 43.7%), *PIK3CA* R88Q (VAF: 45.6%)
MSI status	MSS	MSS	MSS†
TMB	4 Muts/Mb	24 Muts/Mb	286 Muts/Mb
Tumour fraction	Elevated (13%)	Not elevated	Elevated (25%)
Clinical history	Unresectable, moderate to poorly differentiated gallbladder adenocarcinoma. No previous family history of cancer. Patient presented with extensive involvement of the porta hepatis, hepatic artery, portal vein, and liver metastases. She underwent upfront laproscopic cholecystectomy, positive surgical margins (pT2 Nx Mx)	Patien with T4 N1 Mx colorectal adenocarcinoma, moderately differentiated, intestinal type with mucin production. No previous family history of cancer. CT scan revealed the primary tumour within the transverse colon, with invasion into the duodenum and local infiltration into adjacent structures, including the superior mesenteric artery and vein. A few local nodes were identified, but no distant metastases were present	Patient with T3d N2a V2 M1a, poorly differentiated rectal cancer, with extensive bilobar liver metastases. No previous family history of cancer
Treatment details			
Regimen	Cisplatin and gemcitabine with durvalumab	Pembrolizumab	Pembrolizumab
Dose and schedule	Cisplatin (25 mg/m² IV on days 1 and 8) and gemcitabine (1,000 mg/m² IV on days 1 and 8) in combination with durvalumab (1,500 mg IV on day 1) on a 3 week cycle. Maintenance durvalumab (1,500 mg IV on day 1) on a 4 week cycle	Pembrolizumab administered at a rate of 200 mg IV every three weeks for a period of two years, followed by 400 mg IV beyond the two-year timepoint	Pembrolizumab administered at a rate of 200 mg IV every three weeks for a period of two years
Clinical outcomes			
Best radiologic response	iPR	iPR	iCR
Best metabolic response	iCMR	iCMR	iCMR
PFS (months)	23 (event)	32+	58+
OS (months)	33 (censored)	32+	58+
Status at last follow-up	Alive, progressed	Alive, progression-free	Alive, progression-free
Rationale for key clinical decisions			
Choice of systemic treatment	POLE mutation	POLE mutation	*POLE* mutation and high TMB
Treatment modification	Discontinuation of cisplatin and gemcitabine due to chemotoxicity after 4 cycles but continued durvalumab until disease progression. Re-commenced cisplatin and gemcitabine with durvalumab after disease progression to maintain clinical benefit owing to previously attained treatment response	Continued pembrolizumab beyond 2 years at new dose schedule to maintain treatment response and attain symptomatic relief	Not applicable - completed 2 years of pembrolizumab treatment to maintain treatment response
Surgical intervention	Not applicable	Not applicable	Anterior resection and multiple liver lesions owing to excellent response to pembrolizumab and ctDNA negative results on follow-up liquid biopsy. Surgical tumour specimen was ypT0N0M0, R0

* indicates nonsense mutation.

† The MSI status of patient # 3 could not be determined in liquid biopsy results. However, subsequent pre-treatment immunohistochemistry analysis of biopsied tissue specimen revealed MSS status and all 4 proteins including MLH1, MSH2, MSH6 and PMS2 demonstrated normal expression.

+ indicates censored observation at last follow-up.

CT, computed tomography; ctDNA, circulating DNA; iCMR, immunotherapy complete metabolic response; iCR, immunotherapy complete response; iPR, immunotherapy partial response; IV, intravenous; MSI, microsatellite instability; MSS, microsatellite stable, OS, overall survival; PFS, progression-free survival; TMB, tumour mutational burden.

**Figure 2:**
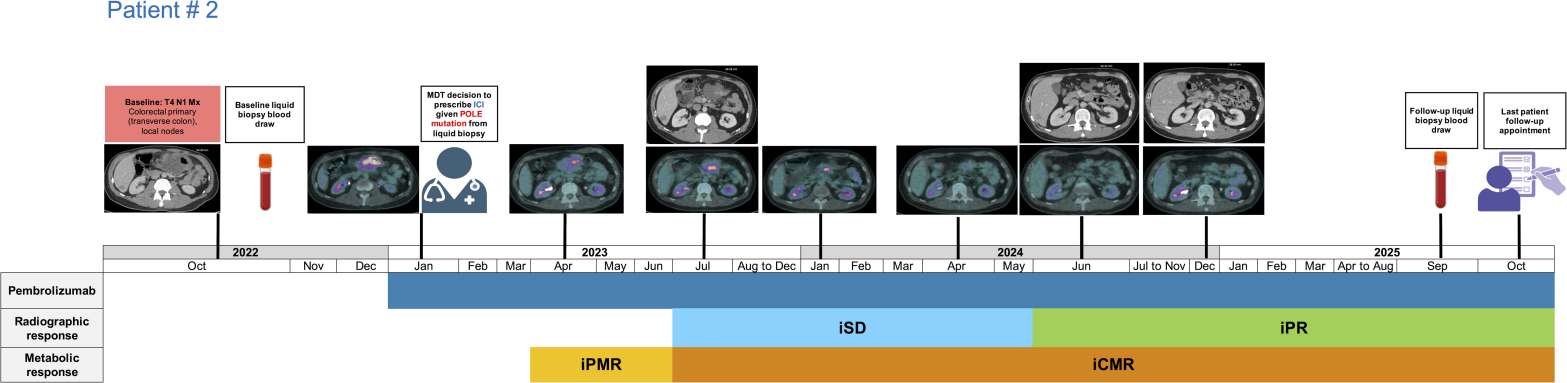
Treatment timeline of patient # 2 receiving long-term pembrolizumab for the management of transverse colon cancer. iCMR, immunotherapy complete metabolic response; iPR, immunotherapy partial response; iPMR, immunotherapy partial metabolic response; iSD, immunotherapy stable disease.

Subject # 2 is a 45-year-old male diagnosed in September 2022 with T4 N1 Mx colorectal adenocarcinoma, moderately differentiated, intestinal type with mucin production (**Figure 2**, **Table 1**). The patient had no prior family history of cancer. A CT scan revealed the primary tumour within the transverse colon, with invasion into the duodenum and local infiltration into adjacent structures, including the superior mesenteric artery and vein. A few local nodes were identified, but no distant metastases were present (**Figure 2**). Notably, the tumour was MMR proficient, and HER-2 negative. Molecular profiling revealed *POLE* P286R (VAF: 0.67%) and *POLE* L1235I mutations. The *POLE* P286R variant is a recurrent missense mutation located within the exonuclease proofreading domain of POLE. This EDM variant is a well-characterised pathogenic alteration and a recognised oncogenic driver in colorectal cancer. According to ACMG/AMP criteria, this meets PS1 (same amino acid change as a known pathogenic variant), PS3 (functional evidence demonstrating impaired proofreading activity) (12–14), PM1 (location in a critical functional domain), and PM2 (absence from population databases such as gnomAD), supporting classification as pathogenic. Although formal mutational signature analysis was not available, the presence of this canonical POLE EDM is strongly associated with COSMIC SBS10a/b and an ultra-mutated phenotype in colorectal cancer. The second variant, *POLE* L1235I, is a missense change located outside the exonuclease domain and involves a conservative amino acid substitution. Consistent with the genomic test report, this variant was classified as a variant of uncertain significance (VUS). It does not meet ACMG/AMP criteria for pathogenicity and was interpreted as a non-EDM. Additional aberrations in key actionable genetic sites were also noted in this patient such as *KRAS* G13D (VAF: 0.41%), *BRCA2* E34* (VAF: 0.19%) and *NF1* splice site 1063-1G>T (VAF: 0.73%) (**Supplementary Tables 2A, B**). The subject was MSS and his TMB was reported as 24 Muts/Mb without an elevated tumour fraction. This patient also had another liquid biopsy test performed in September 2025 which was reported as ctDNA negative with 0% tumour fraction.

**Figure 3:**
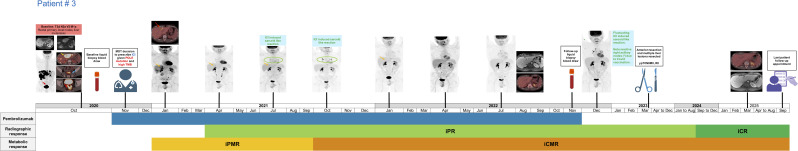
Treatment timeline of patient # 3 receiving pembrolizumab for the management of metastatic rectal cancer. iCMR, immunotherapy complete metabolic response; iCR, immunotherapy complete response; iPR, immunotherapy partial response; iPMR, immunotherapy partial metabolic response.

Patient # 3 is a 30-year-old male diagnosed in October 2020 with stage T3d N2a V2 M1a, poorly differentiated rectal cancer, with extensive bilobar liver metastases (**Figure 3**, **Table 1**). The patient had no prior family history of cancer. Patient’s MSI status could not be determined in liquid biopsy results. However, subsequent pre-treatment immunohistochemistry analysis of biopsied tissue specimen revealed MSS status and all 4 proteins including MLH1, MSH2, MSH6 and PMS2 demonstrated normal expression. Liquid biopsy demonstrated presence of a pathogenic *POLE* EDM at P286R (VAF: 53.0%). Additional *POLE* non-EDM VUS were noted at L1441R, R1382C, R1826W and R2131C and lack ACMG/AMP criteria for pathogenicity. The subject also had mutations in *ATM* R35* (VAF: 41.6%), *BRCA1* E1353*(VAF: 43.8%), *BRCA2* E1593* (VAF: 0.21%), E510* (VAF: 0.39%), E2599* (VAF: 0.44%) and *PIK3CA* E542A (VAF: 43.7%), R88Q (VAF: 45.6%), in addition to other genomic aberrations (**Supplementary Tables 3A, B**). The patient’s microsatellite status could not be determined but his TMB was reported as 286 Muts/Mb with a 25% tumour fraction in the overall sample. This patient also had another liquid biopsy test performed in November 2022 while off treatment where the *POLE* mutations or any other major genetic alterations were no longer identified (**Supplementary Table 3C**). The patient’s microsatellite status could not be determined in this instance but his TMB was reported as 1 Muts/Mb without an elevated tumour fraction.

### Clinical management and treatment outcomes

We observed 3 subjects in our clinical practice who attained an exceptional treatment response and survival outcomes following clinical management of their underlying gastrointestinal cancer with ICIs. All patients were extensively discussed in highly specialized multi-disciplinary meetings (MDT) on a 3 monthly basis during treatment with available re-staging scans. Patients were offered several opportunities to discuss the scope of surgery or discontinuation of immunotherapy and the decision varied in each individual based on the specific clinical context. It is vital to offer personalised care which also includes shared decision making based on unique patient circumstances and preferences.

Patient # 1 with gallbladder cancer commenced post-cholecystectomy cisplatin (25 mg/m² intraveneously (IV) on days 1 and 8) and gemcitabine (1,000 mg/m² IV on days 1 and 8) in combination with durvalumab (1,500 mg IV on day 1) on a 3 week cycle starting in December 2022 owing to the presence of a *POLE* mutation. However, onset of bilateral pulmonary embolism, deep vein thrombosis in the leg, and myelosuppression due to 4 cycles of chemotherapy led to treatment switch to durvalumab monotherapy (1,500 mg IV on day 1) on a 4 week cycle. The subject attained an immunotherapy partial radiological (iPR) and metabolic response (iPMR) after 3 months of systemic treatment (**Figure 1**). An immunotherapy complete metabolic response (iCMR) was achieved 8.11 months after commencing treatment. The iPR was sustained for 21.39 months. In December 2024, re-staging scans demonstrated an increase in size and FDG avidity of the mass around the porta hepatis regarded as unconfirmed progressive disease (iUPD)/unconfirmed progressive metabolic disease (iUPMD) due to absence of a second confirmatory re-staging scan. However, the level of progression warranted re-introduction of cisplatin and gemcitabine alongside durvalumab by institutional MDT. The patient had disease stabilisation for most of the period after recurrence, but recently achieved iPMR in October 2025. Overall, clinical management using a combination of chemo-immunotherapy resulted in an extended favourable response which promoted an OS of over 33 months in this patient. Treatment duration and adjustments were guided by sustained radiologic/metabolic response, clinical tolerance, and MDT recommendations rather than predefined protocols.

The 2^nd^ subject received pembrolizumab in January 2023 for the clinical management of transverse colon cancer administered at a rate of 200 mg IV every three weeks. The patient experienced an iPMR at the first re-staging scan timepoint (**Figure 2**). He continued to experience further tumour shrinkage and attained iCMR in July 2023, which was achieved 5.36 months after starting immunotherapy. The patient also attained iPR in June 2024 which is continued for 15.41 months and currently remains free of distant metastatic disease. Despite the metabolic remission of this patient’s sizable tumour, the patient continued ICI beyond 2 years based on review of serial imaging and MDT recommendation as his primary tumour was symptomatic and to maintain current treatment benefit. Liquid biopsy test results later revealed ctDNA negativity, prompting discontinuation of immunotherapy as of October 2025 followed by a watch and wait approach. This *POLE* mutation directed treatment management strategy has resulted in a long-term survival exceeding 32 months for this patient.

Patient # 3 diagnosed with rectal cancer and liver metastases was offered pembrolizumab from November 2020 to November 2022, administered at a rate of 200 mg IV every three weeks. FDG PET/CT in January 2021 demonstrated an excellent iPMR to ICI, with some sites of disease such as the mesorectal nodes and several liver metastases depicting an iCMR (**Figure 3**). The patient attained iCMR 10.8 months after commencing pembrolizumab. The subject attained an iPR in April 2021, followed by an iCR in September 2024 which was sustained as of the last follow-up timepoint in September 2025, resulting in a duration of radiological response exceeding 53 months. This improvement in overall disease status was further confirmed in a follow-up liquid biopsy test in November 2022 which demonstrated a significant reduction in tumour fraction, TMB, and genetic aberrations compared to the pre-treatment baseline molecular profiling test. Following MDT discussions in March 2023, the subject also underwent an anterior resection and multiple liver resections, with no viable tumour detected in the resected areas. Surgery was performed after achieving sustained metabolic response, and immunotherapy was not restarted postoperatively. Despite the short course of ICI treatment and a poor prognosis, this patient is currently on a 6-month routine surveillance with a PFS and OS exceeding 58 months.

## Discussion

ICIs are reported to be ineffective among the majority of cancer patients with advanced disease (15). In the context of metastatic CRC, favourable response rates ranging from 31.1% - 65% have been limited to a small subset (5%) of MSI-H cases (16). Similarly, the Phase 3 TOPAZ-1 trial demonstrated an overall response rate limited to 26.7% with durvalumab, gemcitabine and cisplatin in biliary tract cancer patients (17). Our study highlights the potential of *POLE* mutation as an alternative predictive biomarker to aid patient selection for ICIs, helping to bridge a critical gap in addressing a major unmet medical need.

Our results align with previous reports in colorectal cancer that *POLE* mutations, which impair DNA replication proofreading, are associated with an ultramutator phenotype (18). These tumours often present with early-onset colorectal or endometrial cancer and carry a higher mutational burden than typical hypermutated cancers. *POLE* mutations are related to other favourable predictive factors such as high expression of PD-L1, high TMB, and infiltration of CD8^+^ cells in the tumour microenvironment (TME) (19, 20). In endometrial cancer, *POLE* P286R is specifically implicated in exerting its anti-tumour effects via activation of the cGAS-STING pathway (21). In our case, the high TMB driven by *POLE* mutations may also reflect the observation of early and sustained ICI treatment response. Although previous *POLE* studies have primarily emphasized the role of pathogenic EDM mutations, recent evidence suggests the increasing prognostic significance of non-EDM *POLE* mutations (22). Chen et al. have demonstrated that POL-non-EDM tumours can exhibit a high neo-antigen burden, and their leukocyte infiltration and CD8+ T-cell infiltration can be similar to POL-EDM tumours (22). Thus, POL-non-EDM tumours could be associated with an activated immune response even in the context of low TMB, suggesting that certain POL mutations may elicit immune activation through mechanisms independent of a hypermutator phenotype. These mechanisms may include the generation of highly immunogenic neoantigens and direct engagement of immune-related pathways, such as those involved in DNA damage response. The study also demonstrated that POL-non-EDM patients achieved significantly higher overall response rates with ICIs than POL-WT patients (58.7% *vs*. 23.8%, p < 0.001), even in TMB-high and TMB-low subgroups (22). Similarly, compared with POL wild type patients, significantly longer OS and PFS were also observed in POL-non-EDM patients (22). In our study, patient’s # 2 and 3 harboured both POLE EDM and POL-non-EDM, potentially contributing to the observed clinical benefit with ICIs via complementary biological pathways. Taken together, these early results suggest that POL-non-EDMs could serve as potential biomarkers to identify ICI responders in addition to POL-EDMs. Additionally, the observation that the lack of *POLE* detection during ICI treatment can be predictive of radiological and metabolic response in CRC further highlights the importance of incorporating ctDNA based tests for serial disease monitoring in future standard of care clinical practice.

Conversely, we observed an exceptional treatment response and prolonged survival in gallbladder cancer despite a low TMB and MSS status. To our knowledge, this is the first case of a patient with GBC harbouring a POLE R150* mutation managed with ICIs and achieving improved outcomes in the current clinical context. Previous studies have assessed nonsynonymous variants in *POLE* and *POLD1* as potential biomarkers for response to ICI suggesting that these variants were independent predictors of prolonged OS, after adjusting for microsatellite stability and cancer type (20). Our finding alludes to the presence of TMB/MSS independent pathways conferring immuno-sensitivity in POLE R150* GBC patients, a hypothesis which warrants further exploration in future investigations. Additionally, expected survival in the absence of other actionable mutations in unresectable GBC patients is less than 1 year. Despite relapse after an iCMR, this patient successfully attained an iPR with re-introduction of chemotherapy to immunotherapy. Presence of a rare *POLE* variant might have influenced the lack of sustained response as compared to other patients. However, it is also possible that the underlying tumour biology of GBCs is different than compared to other GI malignancies which can become immuno-sensitive again with chemotherapy re-challenge to regenerate a metabolic response.

Data regarding management of POLE mutant patients is sparse, but emerging evidence primarily in metastatic colorectal cancer suggests a molecularly guided management approach for ICI use. Recent studies have demonstrated that such patients treated with pembrolizumab using a similar dosing regimen as our patients promotes sustained radiologic and metabolic response and in some instances, post-treatment ctDNA clearance, leading to long term survival benefit in a challenging clinical population (23–25). Decisions regarding immunotherapy duration, timing of surgical intervention following an iCMR, and treatment discontinuation were individualized and made within specialized MDT meetings. In the absence of established guidelines for POLE-mutant gastrointestinal cancers, treatment duration was guided by sustained radiologic and metabolic response, clinical tolerance, and patient preference. Surgical resection was considered only after prolonged and durable metabolic remission, with the aim of confirming pathological response and consolidating disease control rather than as a predefined treatment endpoint. Interpretation of these findings must account for the absence of standardized clinical criteria guiding ICI duration, surgical intervention following an iCMR, or treatment re-challenge in patients with POLE-mutant gastrointestinal cancers. In current practice, such decisions are extrapolated largely from mismatch repair–deficient colorectal cancer data and are further influenced by individual patient factors, disease burden, response kinetics, and treatment tolerance. In our cohort, prolonged ICI therapy, delayed surgical intervention following sustained metabolic remission, and selective discontinuation were guided by multidisciplinary consensus rather than predefined protocols. Accordingly, our observations should not be interpreted as prescriptive but rather as illustrative of a personalized, response-adaptive management approach in a rare molecular subset.

The prevalence of Lynch syndrome (LS) is another important consideration among the GI cancer clinical population. However, there are currently no guidelines recommending LS screening based on POLE mutation alone in an MMR-proficient tumour. ESMO guidelines suggest MMR IHC and/or MSI testing on tumour specimens to identify prevalence of MLH1, MSH2, MSH6 or PMS2 loss before further germline testing for LS is recommended (26). Further LS germline testing is not indicated unless there are additional high-risk factors. In our evaluated patient cohort, all patients had MSS status, thus obviating the need for further germline testing. Additionally, among the two young CRC patients below 50 years of age, none of them had a family history of cancer and did not qualify for further screening based on the NHS pathway at our institution. However, patient # 3 requested germline testing as his brother was also very young, but no evidence of any germline drivers was identified in himself or his family. Additional factors where germline evaluation would be considered include presence of multiple LS-spectrum cancers or clinical suspicion for polymerase proofreading–associated polyposis (PPAP) which were not applicable to our patients. However, this would involve germline *POLE* or *POLD1* mutations which is distinct from LS and associated with colorectal adenomas/cancer and some endometrial cancers. In this case, the germline test would target *POLE/POLD1*, not MMR genes, unless clinical history suggests otherwise.

Our study is limited by the small cohort of patients (n = 3) given the rarity of this mutation and patients, limiting our ability to perform more formal statistical analysis. Additionally, the variability in commercial molecular assays and thresholds for ctDNA and TMB analysis reflects laboratory reporting without universally harmonised thresholds for all tumour types, and interpretive cut-offs for immunotherapy prediction vary across studies and tumour contexts. Moreover, different ctDNA platforms were used for longitudinal monitoring in one patient, which may introduce analytical variability when comparing ctDNA dynamics over time. The report’s retrospective nature inherently limits the ability to draw causal inferences. The presence of heterogeneous treatment regimens and additional genetic mutations in CRC patients could have also influenced outcomes in these subjects and thus the effectiveness of ICI in POLE mutant subjects warrants further investigation in larger clinical populations with standardized molecular criteria.

This study provides a unique case set highlighting the potential role of *POLE* genetic biomarkers in clinical decision making in the first line setting for patients with advanced GI malignancies. Evidence of significant clinical response to ICIs is noteworthy, as is the durability of response. The promising radiological improvement among all patients are substantiated with FDG PET/CT imaging results which further corroborate the functional anti-tumour effect of ICIs via complete resolution of the metabolic activity of underlying disease. The median time to achieve an iCMR for our group of patients was 8.11 months. This highlights the importance of serial FDG PET/CT imaging in such patients to accurately assess tumour functional response status which may not be adequately captured via standard CT scans. The presence of novel *POLE* mutations allowed us to personalize their treatment based on their unique genomic profile, which has resulted in an exceptional survival outcome and quality of life in a challenging clinical population. Identification of genomic biomarkers of ICI response for these patients with a poor prognosis is important given the long-term benefit and reduced side-effects of ICIs compared to other systemic therapies, allowing patients to continue ICIs to ensure greater duration of treatment benefit. Incorporation of minimally invasive ctDNA based liquid biopsies in routine clinical practice is vital to identify *POLE* mutant patients and aid patient stratification for ICI treatment to optimize outcomes in a challenging GI clinical population. While our report demonstrates a favourable response to ICIs in *POLE* EDMs, these findings are hypothesis-generating and require validation in larger, prospective cohorts. Given the rarity of pathogenic *POLE* EDMs across tumour types, future studies should adopt a basket trial design, enrolling patients with advanced solid tumours harbouring confirmed *POLE* EDMs, irrespective of histology. Patients could be stratified by TMB and POLE-associated mutational signatures (e.g., SBS10a/b) to explore correlations with response. The primary endpoints could include objective response rate ORR and PFS, with secondary endpoints including OS and safety. Correlative translational studies could evaluate *POLE* non-EDMs, TMB, mutational signatures, immune infiltrates, and other predictive biomarkers, allowing assessment of the mechanistic basis of ICI responsiveness. Future prospective studies with predefined imaging and serial ctDNA sampling schedules will be necessary to enable robust longitudinal analyses correlating molecular dynamics with radiologic response and relapse. Such a basket trial, leveraging molecular rather than histologic selection, would provide a practical framework to prospectively evaluate the hypothesis that ICIs confer clinical benefit in patients with ultra-mutated *POLE* EDMs, including those with gastrointestinal cancers, while addressing the low prevalence of these mutations across individual tumour types.

## Data Availability

The original contributions presented in the study are included in the article/**Supplementary Material**. Further inquiries can be directed to the corresponding author.
