# Supplementary material for: ctDNA guided management of POLE mutant GI malignancies promotes exceptional responses and prolonged survival to immunotherapy
Source: Front Immunol. 2026 Feb 17;17:1738295. doi: 10.3389/fimmu.2026.1738295 (PMC12953476; doi:10.3389/fimmu.2026.1738295)
Supplement: Supplementary file 1 [file DataSheet1.docx]

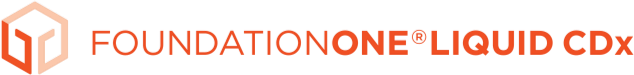


**FoundationOne^®^ Liquid CDx Technical Information**

Table of Content

[1 Intended Use 3](#_Toc541350)

[2 Contraindication 4](#_Toc541351)

[3 Warnings and Precautions 4](#_Toc541352)

[4 Limitations 4](#_Toc541353)

[5 Test Principle 7](#_Toc541354)

[6 FoundationOne Liquid CDx cfDNA Blood Specimen Collection Kit Contents 10](#_Toc541355)

[7 FoundationOne Liquid CDx Test Ordering 10](#_Toc541356)

[8 Instruments 10](#_Toc541357)

[9 Performance Characteristics 10](#_Toc541358)

[9.1 Concordance – Comparison to an Orthogonal cfDNA NGS Method #1 14](#_Toc541359)

[9.2 Concordance – FoundationOne Liquid CDx to validated NGS tumor tissue assay (BRCA1 and BRCA2 alterations) 16](#_Toc541360)

[9.3 Concordance – Comparison to an Orthogonal cfDNA NGS Method #2 17](#_Toc541361)

[9.4 Concordance – FoundationOne Liquid CDx to an externally validated cfDNA NGS assay (SNVs and indels that lead to MET exon 14 skipping) 18](#_Toc541362)

[9.5 Concordance – FoundationOne Liquid CDx to an externally validated cfDNA NGS assay (NTRK1/2/3](#_Toc541363)

[Fusions) 19](#_Toc541364)

[9.6 Concordance – FoundationOne Liquid CDx to an externally validated cfDNA NGS assay (EGFR exon 20 insertions) 20](#_Toc541365)

[9.7 Concordance – FoundationOne Liquid CDx to an externally validated ctDNA NGS assay (BRAF V600E) 21](#_Toc541366)

[9.8 Limit of Detection (Analytical Sensitivity) 22](#_Toc541367)

[9.9 Limit of Blank (LoB) 24](#_Toc541368)

[9.10 Potentially Interfering Substances 25](#_Toc541369)

[9.11 Hybrid Capture Bait Specificity 26](#_Toc541370)

[9.12 Carryover/Cross-Contamination 27](#_Toc541371)

[9.13 Precision: Reproducibility and Confirmation of LoD 27](#_Toc541372)

[9.14 Reagent Lot Interchangeability 32](#_Toc541373)

[9.15 Variant Curator Precision 32](#_Toc541374)

[9.16 Stability 32](#_Toc541375)

[9.17 DNA Extraction 35](#_Toc541376)

[9.18 Guard Banding/Robustness 36](#_Toc541377)

[9.19 Pan-Tumor Performance 38](#_Toc541378)

[9.20 Concordance – FoundationOne Liquid LDT to FoundationOne Liquid CDx 39](#_Toc541379)

[9.21 Molecular Index Barcode Performance 40](#_Toc541380)

[9.22 Automation Line Equivalence 40](#_Toc541381)

[9.23 Updated LC Method Comparison Study 41](#_Toc541382)

[9.24 Updated F1LCDx LC and HC Processes 41](#_Toc541383)

[10 Clinical Validation Studies 45](#_Toc541384)

[10.1 Clinical Bridging Study: Detection of ALK Rearrangements to Determine Eligibility for Treatment with Alectinib 45](#_Toc541385)

[10.2 FoundationOne Liquid CDx Concordance Study for EGFR exon 19 deletion and EGFR exon 21 L858R Alteration 46](#_Toc541386)

[10.3 Clinical Bridging Study: Detection of BRCA1/BRCA2/ATM Alterations to Determine Eligibility for Treatment with olaparib 48](#_Toc541387)

[10.4 Clinical Bridging Study: Detection of BRCA1 and BRCA2 Alterations to Determine Eligibility of mCRPC Patients for Treatment with rucaparib 49](#_Toc541388)

[10.5 Clinical Bridging Study: Detection of PIK3CA Alterations to Determine Eligibility for Treatment with alpelisib 52](#_Toc541389)

[10.6 Clinical Bridging Study: Detection of MET single nucleotide variants (SNVs) and indels that lead to MET exon 14 skipping to Determine Eligibility for Treatment with capmatinib 53](#_Toc541390)

[10.7 Clinical Bridging Study: Detection of ROS1 Fusions to Determine Eligibility for Treatment with](#_Toc541391)

[entrectinib 55](#_Toc541392)

[10.8 Clinical Bridging Study: Detection of NTRK 1/2/3 Fusions to Determine Eligibility for Treatment with entrectinib 58](#_Toc541393)

[10.9 Clinical Bridging Study: Detection of BRAF V600E to Determine Eligibility for Treatment with](#_Toc541394)

[encorafenib in combination with cetuximab 60](#_Toc541395)

[10.10 Clinical Bridging Study: Detection of BRAF V600E to Determine Eligibility for Treatment with](#_Toc541396)

[encorafenib in combination with binimetinib 64](#_Toc541397)

[10.11 Clinical Bridging Study: Detection of BRCA1 and BRCA2 Alterations to Determine Eligibility for Treatment with niraparib + abiraterone acetate 66](#_Toc541398)

[10.12 Clinical Bridging Study: Detection of BRCA1 and BRCA2 Alterations to Determine Eligibility for Treatment with LYNPARZA® in combination with abiraterone 69](#_Toc541399)

[10.13 FoundationOne Liquid CDx Clinical Efficacy Study: Detection of PIK3CA mutations to determine eligibility with ITOVEBI™ (inavolisib) in combination with palbociclib and fulvestrant 71](#_Toc541400)

[10.14 Clinical Bridging Study: Detection of MET single nucleotide variants (SNVs) and indels that lead](#_Toc541401)

[to MET exon 14 skipping to Determine Eligibility for Treatment with tepotinib 73](#_Toc541402)

[11 CDx Classification Criteria 75](#_Toc541403)

[11.1 CDx classification criteria for ALK rearrangements, qualifying NSCLC patients for therapy with ALECENSA® (alectinib): 75](#_Toc541404)

[11.2 CDx classification criteria for EGFR alterations, qualifying NSCLC patients for therapy with EGFR Tyrosine Kinase Inhibitors (TKI) approved by FDA: 75](#_Toc541405)

[11.3 CDx classification criteria for BRCA1, BRCA2, and ATM alterations, qualifying prostate cancer patients for therapy with LYNPARZA® (olaparib): 75](#_Toc541406)

[11.4 CDx classification criteria for BRCA1 and BRCA2 alterations, qualifying prostate cancer patients for therapy with RUBRACA® (rucaparib): 77](#_Toc541407)

[11.5 CDx classification criteria for PIK3CA alterations, qualifying breast cancer patients for therapy with PIQRAY® (alpelisib): 77](#_Toc541408)

[11.6 CDx classification criteria for SNVs and Indels that lead to MET exon 14 skipping: 77](#_Toc541409)

[11.7 CDx classification criteria for NTRK fusions: 78](#_Toc541410)

[11.8 CDx classification criteria for ROS1 fusions 78](#_Toc541411)

[11.9 CDx classification criteria for BRAF V600E 78](#_Toc541412)

[11.10 CDx classification criteria for BRCA1 and BRCA2 alterations, qualifying prostate cancer patients](#_Toc541413)

[for therapy with AKEEGA® (niraparib + abiraterone acetate) 78](#_Toc541414)

[11.11 CDx classification criteria for BRCA1 and BRCA2 alterations, qualifying prostate cancer patients](#_Toc541415)

[for therapy with LYNPARZA® in combination with abiraterone 79](#_Toc541416)

[11.12 CDx classification criteria for *PIK3CA* alterations, qualifying breast cancer patients for therapy with ITOVEBI™ (inavolisib) in combination with with palbociclib and fulvestrant 79](#_Toc541417)


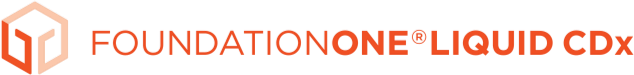


FoundationOne^®^ Liquid CDx Technical Information

Foundation Medicine, Inc.

150 Second Street, Cambridge, MA 02141

Phone: (617) 418-2200

#### 1 Intended Use

FoundationOne Liquid CDx is a qualitative next generation sequencing based *in vitro* diagnostic test that uses targeted high throughput hybridization-based capture technology to detect and report genomic alterations in 311 genes. These include substitutions, insertions and deletions (indels) in 311 genes, rearrangements in 8 genes and copy number alterations in 3 genes. FoundationOne Liquid CDx utilizes circulating cell-free DNA (cfDNA) isolated from plasma derived from anti-coagulated peripheral whole blood of cancer patients collected in FoundationOne Liquid CDx cfDNA blood collection tubes included in the FoundationOne Liquid CDx Blood Sample Collection Kit. The test is intended to be used as a companion diagnostic to identify patients who may benefit from treatment with the targeted therapies listed in **Table 1** in accordance with the approved therapeutic product labeling.

Table 1. Companion diagnostic indications

| **Tumor Type** | **Biomarker(s) Detected** | **Therapy** |
| --- | --- | --- |
| Breast cancer | *PIK3CA* mutations | ITOVEBI™ (inavolisib) in combination with palbociclib and fulvestrant |
|  | *PIK3CA* mutations C420R, E542K, E545A,  E545D [1635G>T only], E545G, E545K,  Q546E, Q546R; and H1047L, H1047R, and H1047Y | PIQRAY® (alpelisib) |
| Colorectal cancer (CRC) | *BRAF* V600E | BRAFTOVI® (encorafenib) in combination with cetuximab |
| Non-small cell lung cancer (NSCLC) | *ALK* rearrangements | ALECENSA® (alectinib) |
|  | *BRAF* V600E | BRAFTOVI® (encorafenib) in combination with MEKTOVI®  (binimetinib) |
|  | *EGFR* exon 19 deletions and  *EGFR* exon 21 L858R substitution | EGFR tyrosine kinase inhibitors approved by FDA* |
|  | *MET* single nucleotide variants (SNVs) and indels that lead *to MET* exon 14 skipping | TABRECTA® (capmatinib) |
|  |  | TEPMETKO® (tepotinib)** |
|  | *ROS1* fusions**** | ROZLYTREK® (entrectinib) |

| **Tumor Type** | **Biomarker(s) Detected** | **Therapy** |
| --- | --- | --- |
| Prostate cancer | *ATM, BRCA1, BRCA2* alterations | LYNPARZA® (olaparib) |
|  | *BRCA1*, *BRCA2* alterations | AKEEGA® (niraparib + abiraterone acetate) |
|  |  | LYNPARZA® (olaparib) in combination with abiraterone |
|  |  | RUBRACA® (rucaparib) |
| Solid tumors | *NTRK1/2/3* fusions** | ROZLYTREK® (entrectinib) |

*For the most current information about the therapeutic products in this group, go to: [https://www.fda.gov/medical-devices/in-vitro-diagnostics/list-cleared-or-approved-companion-diagnostic-devices-in-vitro-and-imaging- tools#Group_Labeling](https://www.fda.gov/medical-devices/in-vitro-diagnostics/list-cleared-or-approved-companion-diagnostic-devices-in-vitro-and-imaging-tools#Group_Labeling)

****** When considering eligibility for ROZLYTREK® based on the detection of *NTRK1/2/3* and *ROS1* fusions or for TEPMETKO® based on the detection of *MET* SNVs and indels that lead to *MET* exon 14 skipping, testing using plasma specimens is only appropriate for patients for whom tumor tissue is not available for testing.

Additionally, FoundationOne Liquid CDx is intended to provide tumor mutation profiling to be used by qualified health care professionals in accordance with professional guidelines in oncology for patients with solid malignant neoplasms.

A negative result from a plasma specimen does not mean that the patient’s tumor is negative for genomic findings. Patients with the tumor types above who are negative for the mutations listed in **Table 1** (see ** footnote under **Table 1** for *NTRK1/2/3* and *ROS1* fusions for ROZLYTREK®and *MET* SNVs and indels that lead to *MET* exon 14 skipping forTEPMETKO®) should be reflexed to routine biopsy and their tumor mutation status confirmed using an FDA-approved tumor tissue test, if feasible.

Genomic findings other than those listed in **Table 1** are not prescriptive or conclusive for labeled use of any specific therapeutic product.

FoundationOne Liquid CDx is a single-site assay performed at Foundation Medicine, Inc. in Cambridge, MA.

#### 2 Contraindication

There are no known contraindications.

#### 3 Warnings and Precautions

1. Alterations reported may include somatic (not inherited) or germline (inherited) alterations; however, the test does not distinguish between germline and somatic alterations. If a reported alteration is suspected to be germline, confirmatory testing should be considered in the appropriate clinical context.

1. The test is not intended to replace germline testing or to provide information about cancer predisposition.

1. Patients for whom no companion diagnostic alterations are detected should be considered for confirmation with an FDA-approved tumor tissue test, if possible.

#### 4 Limitations

1. For *in vitro* diagnostic use only.

1. For prescription use only. This test must be ordered by a qualified medical professional in accordance with clinical laboratory regulations.

1. Genomic findings other than those listed in **Table 1** of the intended use are not prescriptive or conclusive for labeled use of any specific therapeutic product.

1. A negative result does not rule out the presence of an alteration in the patient’s tumor.

1. Decisions on patient care and treatment must be based on the independent medical judgment of the treating physician, taking into consideration all applicable information concerning the patient’s condition, such as patient and family history, physical examinations, information from other diagnostic tests, and patient preferences, in accordance with the standard of care in a given community.

1. The test is intended to be performed on specific serial number-controlled instruments by Foundation Medicine, Inc.

1. Genomic findings from cfDNA may originate from circulating tumor DNA fragments, germline alterations, or nontumor somatic alterations, such as clonal hematopoiesis of indeterminate potential (CHIP). Genes with alterations that may be derived from CHIP include, but are not limited to, the following: *ASXL1, ATM, CBL, CHEK2, DNMT3A, JAK2, KMT2D (MLL2), MPL, MYD88, SF3B1, TET2, TP53*, and *U2AF1*. The efficacy of targeting such nontumor somatic alterations (e.g., CH) is unknown.

1. The analytical accuracy for the FoundationOne Liquid CDx assay has not been demonstrated in all genes.

1. The analytical accuracy for the FoundationOne Liquid CDx assay for the detection of SNVs and indels that lead to *MET* exon 14 skipping has not been demonstrated for samples with variant allele frequencies (VAF) below 0.34% for base substitutions and 0.73% VAF for small insertions and small deletions.

1. TABRECTA® efficacy has not been established in patients with *MET* SNVs <0.21% VAF and in patients with *MET* indels <0.16% VAF tested with FoundationOne Liquid CDx.

1. TEPMETKO® efficacy has not been established in patients with *MET* SNVs <0.19% VAF and in patients with *MET* indels <0.10% VAF tested with FoundationOne Liquid CDx.

12. ALECENSA® efficacy has not been established in patients with *ALK* rearrangements <0.06% VAF tested with FoundationOne Liquid CDx.

1. LYNPARZA® efficacy has not been established in prostate cancer patients with *BRCA1/2* or *ATM* rearrangements with <0.25% VAF or with short variants in *BRCA1/2* or *ATM* <0.11% VAF tested with FoundationOne Liquid CDx.

1. RUBRACA® efficacy has not been established in prostate cancer patients with *BRCA1/2* rearrangements with <0.85% VAF or with short variants in *BRCA1/2* <0.15% VAF tested with FoundationOne Liquid CDx.

1. PIQRAY® efficacy has not been established in patients with *PIK3CA* SNVs with <0.14% VAF tested with FoundationOne Liquid CDx.

1. BRAFTOVI® (encorafenib) in combination with cetuximab efficacy has not been established in patients with the *BRAF* V600E with <0.11 % VAF tested with FoundationOne Liquid CDx.

1. BRAFTOVI® (encorafenib) in combination with MEKTOVI® (binimetinib) efficacy has not been established in patients with *BRAF* V600E with < 0.099% VAF tested with FoundationOneLiquid CDx.

1. The precision of FoundationOne Liquid CDx was only confirmed for select variants at the limit of detection (LoD).

1. The FoundationOne Liquid CDx assay does not detect heterozygous deletions.

1. The FoundationOne Liquid CDx assay does not detect copy number losses/homozygous deletions in *ATM*.

1. A complete assessment of the impact of cfDNA blood collection tube lot-to-lot variability on the performance of the test has not been evaluated.

1. The test is not intended to provide information on cancer predisposition.

1. *BRCA1/BRCA2* homozygous deletions and rearrangements were not adequately represented in all analytical studies.

1. Representation of *ALK* rearrangements were limited in the analytical validation studies.

1. The representation of *ATM* short variants and rearrangements was limited in the analytical validation studies.

1. Performance has not been validated for cfDNA input below the specified minimum input.

1. Representation of SNV and indels that lead to *MET* exon 14 skipping that represent biomarker rule category 1 and 2 (refer to Section 11.6 for the companion diagnostic biomarker definition), were limited in the analytical validation studies.

28. For optimal ctDNA shed, it is recommended that blood be drawn prior to therapy or at a time of disease progression. The sensitivity of liquid biopsy is related to adequate levels of ctDNA shed. Therefore, assay performance will be dependent upon level of ctDNA shed at time of testing.

1. Due to the low prevalence of *ROS1* fusions and *NTRK1/2/3* fusions, the positive predictive value (PPV) of the test (FoundationOne Liquid CDx positive, tissue negative) may be lower than reported in test labeling.

1. FoundationOne Liquid CDx may miss a subset of patients with *NTRK1/2/3* fusion and *ROS1* fusion positive solid tumors who may derive benefit from ROZLYTREK®. In a retrospective-prospective clinical study assessing concordance between FoundationOne Liquid CDx test results in plasma and patients whose tumor tissue tested positive and was the basis for enrollment into a clinical trial, the data demonstrated that the FoundationOne Liquid CDx test did not detect approximately 46% of potential responders with *NTRK1/2/3* fusions and 49% of responders with *ROS1* fusions.

1. ROZLYTREK® efficacy has not been established in patients with *NTRK2* fusions tested with FoundationOne Liquid CDx, given the low prevalence of the biomarker.

1. In a retrospective-prospective clinical study assessing concordance between FoundationOne Liquid CDx test results in plasma and patients whose tumor tissue tested positive and was the basis for enrollment into a clinical trial, FoundationOne Liquid CDx detected 1 of 7 different *NTRK3* fusion partners. Due to the rarity of these fusions, the accuracy of FoundationOne Liquid CDx for *NTRK3* fusions has not been adequately determined.

1. *NTRK2* fusions per the FoundationOne Liquid CDx biomarker rules for *NTRK1/2/3* fusions were not represented in analytical validation studies.

1. A study evaluating the concordance to a second method demonstrated that the agreement between FoundationOne Liquid CDx positive results and a comparator method for *NTRK1/3*, and *ROS1* was ≤ 50% (i.e., whether these are potential FoundationOne Liquid CDx false positives or false negatives by the comparator is unknown).

1. FoundationOne Liquid CDx may not detect a subset of patients with prostate cancer with *BRCA1/2* homozygous deletions who may derive benefit from AKEEGA. A subgroup analysis indicated that the FoundationOne Liquid CDx test did not detect approximately 81% (21/26) of prostate cancer patients with *BRCA1/2* homozygous deletions detected by FoundationOne CDx who derived benefit from AKEEGA. All samples were below the limit of detection for *BRCA2* copy number loss by the FoundationOne Liquid CDx assay. Additional testing to confirm the absence of *BRCA1/2* homozygous deletions in the patient’s tumor tissue with another FDA approved test, if feasible, is strongly recommended. (Around 2.72% of patients with prostate cancer are expected to harbor *BRCA1/2* homozygous deletions, based on FMI internal data).

1. Results reported may include variants with very low variant allele frequency (SNVs <0.5% VAF and indels <1.0% VAF). Such alterations may be false positive results detected due to background signals inherent in sequencing methods designed for high sensitivity or due to clonal hematopoiesis of indeterminate potential (CHIP). Additional clinical investigation to confirm the presence of these variants in the patient’s tumor with another FDA-approved or cleared test is strongly recommended.

1. *BRCA1/2* homozygous deletions may not be detected in a subset of prostate cancer patients by FoundationOne Liquid CDx. Additional testing to confirm the absence of *BRCA1/2* homozygous deletions in the patient’s tumor tissue with another FDA approved test, if feasible, is strongly recommended.

1. FoundationOne Liquid CDx may miss a subset of patients with *MET* single nucleotide variants (SNVs) and indels that lead to *MET* exon 14 skipping alterations in NSCLC patients who may derive benefit from TEPMETKO®. In a retrospective clinical study assessing concordance between FoundationOne Liquid CDx test results in plasma and patients whose plasma or tumor tissue tested positive and was the basis for enrollment into a clinical trial, the data demonstrated that the FoundationOne Liquid CDx test did not detect approximately 33% of responders with *MET* single nucleotide variants (SNVs) and indels that lead to *MET* exon 14 skipping alterations. Non-detection of *MET* exon 14 skipping alterations in these samples was attributed to low tumor fraction.

#### 5 Test Principle

The FoundationOne Liquid CDx assay is performed exclusively as a laboratory service using circulating cfDNA isolated from plasma derived from anti-coagulated peripheral whole blood from patients with solid malignant neoplasms. The assay employs a single DNA extraction method to obtain cfDNA from plasma from whole blood. Extracted cfDNA undergoes whole-genome shotgun library construction and hybridization-based capture of 324 cancer-related genes. All coding exons of 309 genes are targeted; select intronic or non-coding regions are targeted in 15 of these genes (refer to **Table 2** for the complete list of genes interrogated by FoundationOne Liquid CDx). Hybrid-capture selected libraries are sequenced with deep coverage using the NovaSeq® 6000 platform. Sequence data are processed using a custom analysis pipeline designed to detect genomic alterations in 311 genes. These include base substitutions and indels in 311 genes, copy number alterations in 3 genes, and gene rearrangements in 8 genes. A subset of targeted regions in 75 genes is baited for enhanced sensitivity.

**Table 2. The FoundationOne Liquid CDx assay interrogates 324 genes, including 309 genes with complete exonic (coding) coverage and 15 genes with only select non-coding coverage (indicated with an asterisk).**

Select regions in 75 genes (indicated in bold) are captured with increased sensitivity. Genes are captured for increased sensitivity with complete exonic coverage unless otherwise noted.

| ***ABL1***  ***[Exons 4-***  ***9]*** | *ACVR1B* | ***AKT1***  ***[Exon 3]*** | *AKT2* | *AKT3* | ***ALK***  ***[Exons 20-***  ***29, Introns***  ***18,19]*** | *ALOX12B* | *AMER1 (FAM123B)* | ***APC*** | ***AR*** |
| --- | --- | --- | --- | --- | --- | --- | --- | --- | --- |
| ***ARAF***  ***[Exons 4,***  ***5, 7, 11, 13,***  ***15, 16]*** | *ARFRP1* | *ARID1A* | *ASXL1* | ***ATM*** | ***ATR*** | *ATRX* | *AURKA* | *AURKB* | *AXIN1* |
| *AXL* | *BAP1* | *BARD1* | *BCL2* | *BCL2L1* | *BCL2L2* | *BCL6* | *BCOR* | *BCORL1* | *BCR**  *[Introns 8,*  *13, 14]* |
| ***BRAF***  ***[Exons 11-***  ***18****, Introns*  *7-10]* | ***BRCA1***  *[Introns 2,*  *7, 8, 12, 16,*  *19, 20]* | ***BRCA2***  *[Intron 2]* | *BRD4* | *BRIP1* | *BTG1* | *BTG2* | ***BTK***  ***[Exons 2,***  ***15]*** | *C11orf30 (EMSY)* | *C17orf39 (GID4)* |
| *CALR* | *CARD11* | *CASP8* | *CBFB* | *CBL* | ***CCND1*** | *CCND2* | *CCND3* | *CCNE1* | *CD22* |
| *CD70* | *CD74**  *[Introns 6- 8]* | *CD79A* | *CD79B* | ***CD274 (PD- L1)*** | *CDC73* | ***CDH1*** | ***CDK12*** | ***CDK4*** | ***CDK6*** |
| *CDK8* | *CDKN1A* | *CDKN1B* | ***CDKN2A*** | *CDKN2B* | *CDKN2C* | *CEBPA* | *CHEK1* | ***CHEK2*** | *CIC* |
| *CREBBP* | ***CRKL*** | *CSF1R* | *CSF3R* | *CTCF* | *CTNNA1* | ***CTNNB1***  ***[Exon 3]*** | *CUL3* | *CUL4A* | *CXCR4* |
| *CYP17A1* | *DAXX* | *DDR1* | ***DDR2***  ***[Exons 5,***  ***17, 18]*** | *DIS3* | *DNMT3A* | *DOT1L* | *EED* | ***EGFR***  ***[Introns 7,***  *15,* ***24-27]*** | *EP300* |
| *EPHA3* | *EPHB1* | *EPHB4* | ***ERBB2*** | ***ERBB3***  ***[Exons 3,***  ***6, 7, 8, 10,***  ***12, 20, 21,***  ***23, 24, 25]*** | *ERBB4* | *ERCC4* | *ERG* | ***ERRFI1*** | ***ESR1***  ***[Exons 4-***  ***8]*** |
| *ETV4**  *[Intron 8]* | *ETV5**  *[Introns 6, 7]* | ***ETV6****  ***[Introns 5, 6]*** | *EWSR1**  *[Introns 7-*  *13]* | ***EZH2***  ***[Exons 4,***  ***16, 17, 18]*** | *EZR**  *[Introns 9-*  *11]* | *FAM46C* | *FANCA* | *FANCC* | *FANCG* |
| *FANCL* | *FAS* | *FBXW7* | *FGF10* | *FGF12* | *FGF14* | *FGF19* | *FGF23* | *FGF3* | *FGF4* |
| *FGF6* | ***FGFR1***  *[Introns 1,*  *5,* ***Intron***  ***17]*** | ***FGFR2***  *[Intron 1,*  ***Intron 17]*** | ***FGFR3***  ***[Exons 7, 9***  ***(alternative designation***  ***exon 10),***  ***14, 18,***  ***Intron 17]*** | *FGFR4* | *FH* | *FLCN* | *FLT1* | ***FLT3***  ***[Exons 14,***  ***15, 20]*** | ***FOXL2*** |
| *FUBP1* | *GABRA6* | *GATA3* | *GATA4* | *GATA6* | ***GNA11***  ***[Exons 4,***  ***5]*** | *GNA13* | ***GNAQ***  ***[Exons 4,***  ***5]*** | ***GNAS***  ***[Exons 1, 8]*** | *GRM3* |
| *GSK3B* | *H3F3A* | *HDAC1* | *HGF* | *HNF1A* | ***HRAS***  ***[Exons 2,***  ***3]*** | *HSD3B1* | *ID3* | ***IDH1 [Exon 4]*** | ***IDH2 [Exon 4]*** |
| *IGF1R* | *IKBKE* | *IKZF1* | *INPP4B* | *IRF2* | *IRF4* | *IRS2* | *JAK1* | ***JAK2***  ***[Exon 14]*** | ***JAK3***  ***[Exons 5,***  ***11, 12, 13,***  ***15, 16]*** |
| *JUN* | *KDM5A* | *KDM5C* | *KDM6A* | *KDR* | *KEAP1* | *KEL* | ***KIT***  ***[Exons***  ***8,9,11,12,***  ***13, 17****,*  *Intron 16]* | *KLHL6* | *KMT2A*  *(MLL)*  *[Introns 6,*  *8-11,*  *Intron 7]* |
| *KMT2D (MLL2)* | ***KRAS*** | *LTK* | *LYN* | *MAF* | ***MAP2K1***  ***(MEK1)***  ***[Exons 2,***  ***3]*** | ***MAP2K2***  ***(MEK2)***  ***[Exons 2-***  ***4, 6, 7]*** | *MAP2K4* | *MAP3K1* | *MAP3K13* |
| *MAPK1* | *MCL1* | ***MDM2*** | *MDM4* | *MED12* | *MEF2B* | *MEN1* | *MERTK* | ***MET*** | *MITF* |
| *MKNK1* | *MLH1* | ***MPL***  ***[Exon 10]*** | *MRE11A* | *MSH2*  *[Intron 5]* | *MSH3* | *MSH6* | *MST1R* | *MTAP* | ***MTOR***  ***[Exons 19,***  ***30, 39***  ***40, 43-45,***  ***47, 48, 53, 56]*** |
| *MUTYH* | *MYB**  *[Intron 14]* | ***MYC***  ***[Intron 1]*** | *MYCL (MYCL1)* | ***MYCN*** | ***MYD88***  ***[Exon 4]*** | *NBN* | ***NF1*** | *NF2* | *NFE2L2* |
| *NFKBIA* | *NKX2-1 (TTF-*  *1)* | *NOTCH1* | *NOTCH2*  *[Intron 26]* | *NOTCH3* | ***NPM1***  ***[Exons 4- 6, 8, 10]*** | ***NRAS***  ***[Exons 2,***  ***3]*** | *NSD3 (WHSC1L1)* | *NT5C2* | ***NTRK1***  ***[Exons 14,***  ***15,***  ***Introns 8- 11]*** |
| *NTRK2*  *[Intron 12]* | ***NTRK3***  ***[Exons 16, 17]*** | *NUTM1**  *[Intron 1]* | *P2RY8* | ***PALB2*** | *PARK2* | *PARP1* | *PARP2* | *PARP3* | *PAX5* |
| *PBRM1* | *PDCD1 (PD-*  *1)* | ***PDCD1LG2***  ***(PD-L2)*** | ***PDGFRA***  ***[Exons 12,***  ***18, Introns***  ***7, 9, 11]*** | ***PDGFRB***  ***[Exons 12-***  ***21, 23]*** | *PDK1* | *PIK3C2B* | *PIK3C2G* | ***PIK3CA***  ***[Exons 2, 3,***  ***5-8, 10, 14,***  ***19, 21***  ***(Coding***  ***Exons 1, 2,***  ***4-7, 9, 13,***  ***18, 20)]*** | *PIK3CB* |
| *PIK3R1* | *PIM1* | *PMS2* | *POLD1* | *POLE* | *PPARG* | *PPP2R1A* | *PPP2R2A* | *PRDM1* | *PRKAR1A* |
| *PRKCI* | *PTCH1* | ***PTEN*** | ***PTPN11*** | *PTPRO* | *QKI* | *RAC1* | *RAD21* | *RAD51* | *RAD51B* |
| *RAD51C* | *RAD51D* | *RAD52* | *RAD54L* | ***RAF1***  ***[Exons 3,***  ***4, 6, 7, 10, 14, 15, 17,***  *Introns 4-8]* | *RARA*  *[Intron 2]* | ***RB1*** | *RBM10* | *REL* | ***RET***  ***[****Introns 7,*  *8,* ***Exons***  ***11, 13-16,***  ***Introns 9- 11]*** |
| *RICTOR* | *RNF43* | ***ROS1***  ***[Exons 31,***  ***36-38, 40,***  ***Introns 31- 35]*** | *RPTOR* | *RSPO2**  *[Intron 1]* | *SDC4**  *[Intron 2]* | *SDHA* | *SDHB* | *SDHC* | *SDHD* |
| *SETD2* | *SF3B1* | *SGK1* | *SLC34A2**  *[Intron 4]* | *SMAD2* | *SMAD4* | *SMARCA4* | *SMARCB1* | ***SMO*** | *SNCAIP* |
| *SOCS1* | *SOX2* | *SOX9* | *SPEN* | *SPOP* | *SRC* | *STAG2* | *STAT3* | ***STK11 (LKB1)*** | *SUFU* |
| *SYK* | *TBX3* | *TEK* | *TERC* {ncRNA}* | ***TERT* {Promoter}*** | *TET2* | *TGFBR2* | *TIPARP* | *TMPRSS2**  *[Introns 1-3]* | *TNFAIP3* |
| *TNFRSF14* | ***TP53*** | *TSC1* | *TSC2* | *TYRO3* | *U2AF1* | ***VEGFA*** | *VHL* | *WHSC1* | *WT1* |
| *XPO1* | *XRCC2* | *ZNF217* | *ZNF703* |  | | | | | |

The classification criteria for all companion diagnostic (CDx) variants are outlined at the end of this document. The output of the test includes:

Category 1: CDx claims noted in **Table 1** of the Intended Use

Category 2: cfDNA Biomarkers with Strong Evidence of Clinical Significance in cfDNA

Category 3: Biomarkers with Evidence of Clinical Significance in tissue supported by:

3A: strong analytical validation using cfDNA

3B: analytical validation using cfDNA

Category 4: Other Biomarkers with Potential Clinical Significance

As part of its FDA-approved intended use, copy number alterations and rearrangements are reported in the genes listed in **Table 3**.

Table 3. Genes for which copy number alterations and rearrangements are reported for tumor profiling by FoundationOne Liquid CDx

| **Alteration Type** | **Genes** |
| --- | --- |
| Copy Number Alterations | *BRCA1, BRCA2, ERBB2* |
| Rearrangements | *ALK, BRCA1, BRCA2, NTRK1, NTRK2, NTRK3* |

#### 6 FoundationOne Liquid CDx cfDNA Blood Specimen Collection Kit Contents

Test Kit Contents

The test includes a sample shipping kit, which is sent to ordering laboratories and physicians. The shipping kit contains the following components:

1. Specimen preparation and shipping instructions
2. Two FoundationOne Liquid CDx cfDNA blood collection tubes (8.5 mL nominal fill volume per tube)
3. Return shipping label

All other reagents, materials and equipment needed to perform the assay are used exclusively in the Foundation Medicine laboratory. The FoundationOne Liquid CDx assay is intended to be performed with serial number-controlled instruments.

#### 7 FoundationOne Liquid CDx Test Ordering

To order FoundationOne Liquid CDx, the test order form in the test kit must be fully completed and signed by the ordering physician or other authorized medical professional. Please refer to Specimen Preparation Instructions and Shipping Instructions included in the test kit.

#### 8 Instruments

The FoundationOne Liquid CDx device is intended to be performed with the following instruments, as identified by specific serial numbers:

Illumina NovaSeq 6000

Thermo Scientific Kingfisher Flex DW 96

Hamilton STARTlet-STAR Liquid Handling Workstation

#### 9 Performance Characteristics

Performance characteristics were established using contrived and clinical circulating cfDNA derived from blood specimens extracted from a wide range of tumor types. **Table 4** below provides a summary of the number of tumor types and variants included in each study. As summarized in this table, each study included a broad range of representative alteration types (substitutions, insertions and deletions, rearrangements, and copy number alterations) in various genomic contexts across a number of genes. The validation studies included >7,000 sample replicates, >31,000 unique variants [includes variants classified as variants of unknown significance (VUS) and/or benign], and >30 tumor types, representing all 324 genes targeted by the FoundationOne Liquid CDx assay.

Table 4. Representation of tumor types and variants^1^ across validation studies

| **Study Title** | **Cancer Types Represented** | **# Unique Samples** | **# of**  **Sample**  **Replicates** | **# of Unique Genes** | **# of Unique** | | | | |
| --- | --- | --- | --- | --- | --- | --- | --- | --- | --- |
|  |  |  |  |  | **Subs** | **Indels** | **Rearrang.** | **Copy**  **Number**  **Amplif.** | **Copy**  **Number**  **Losses** |
| Contrived Sample  Functional  Characterization  (CSFC) Study | Breast cancer  Colorectal cancer  Lung cancer  Contrived samples | 13 | 1843 | 228 | 563 | 81 | 11 | 1 | 1 |
| FoundationOne  Liquid CDx to  Validated NGS  Tumor Tissue Test Concordance:  *BRCA1* and *BRCA2*  Variants | Prostate cancer Ovarian cancer | 279 | N/A | 2 | 100 | 87 | 9 | 0 | 2 |
| FoundationOne  Liquid CDx to Validated NGS cfDNA Assay Concordance:  *PIK3CA* mutations | Breast cancer | 412 | N/A | 1 | 32 | 5 | 0 | 0 | 0 |
| Orthogonal Concordance | 23 cancer types  Contrived samples | 278 | N/A | 64 | 541 | 12 | 11 | 3 | 0 |
| LoD Estimation | Prostate Contrived samples | 10 | 877 | 286 | 1490 | 247 | 32 | 13 | 3 |
| LoB Study 1 | Healthy Donors | 28 | 79 | 322 | 26134 | 4482 | 911 | 222 | 42 |
| LoB Study 2^3^ | Healthy Donors | 44 | 131 | 532 | 29507 | 4438 | 2752 | 222 | 42 |
| Potentially  Interfering  Substances | Contrived samples | 9 | 336 | 18 | 16 | 11 | 11 | 1 | 2 |
| Hybrid Capture Bait Specificity | 25 cancer types  Contrived samples | 3546 | N/A | 324 | N/A | N/A | N/A | N/A | N/A |
| Reagent Stability | Contrived samples | 8 | 142 | 279 | 1090 | 215 | 32 | 17 | 2 |
| Reagent  Interchangeability | Contrived samples | 8 | 192 | 20 | 15 | 11 | 11 | 1 | 1 |
| Platform Precision study 1 | Breast cancer  Colon cancer  Lung cancer  Ovarian cancer  Prostate cancer Skin cancer  Contrived samples | 47 | 1121 | 280 | 900 | 229 | 63 | 49 | 5 |
| Platform Precision study 2 | Lung cancer  Prostate cancer  Stomach cancer  Colorectal cancer Bile duct cancer  Breast cancer | 10 | 230 | 6 | 6 | 4 | 0 | 0 | 0 |
| Precision of detection of SNVs  and indels that lead to *MET* exon 14 skipping (Precision study 3) | Lung Cancer | 5 | 166 | 1 | 2 | 3 | N/A | N/A | N/A |

| **Study Title** | **Cancer Types Represented** | **# Unique Samples** | **# of**  **Sample**  **Replicates** | **# of Unique Genes** | **# of Unique** | | | | |
| --- | --- | --- | --- | --- | --- | --- | --- | --- | --- |
|  |  |  |  |  | **Subs** | **Indels** | **Rearrang.** | **Copy**  **Number**  **Amplif.** | **Copy**  **Number**  **Losses** |
| Platform Precision study 4^3^ | Ovarian cancer  Prostate cancer  Breast cancer  Lung cancer Colon  adenocarcinoma Soft tissue neuroblastoma | 17 | 402 | 159 | 258 | 43 | 6 | 22 | 1 |
| DNA Extraction | Colorectal cancer  Prostate cancer  Breast cancer  Lung cancer  Skin cancer | 6 | 72 | 161 | 265 | 53 | 2 | 0 | 0 |
| Whole Blood Sample  Stability | Lung cancer  Colorectal cancer  Gastrointestinal (non-Colorectal cancer)  Prostate cancer  Breast cancer  Ovarian cancer | 74 | 148 | 206 | 490 | 76 | 12 | 14 | 0 |
| Inverted Tube Whole  Blood Sample  Stability | Lung cancer  Colorectal cancer  Breast cancer  Ovarian cancer  Prostate cancer | 156 | 312 | 280 | 1295 | 195 | 19 | 27 | 0 |
| Cross Contamination | Contrived samples | 5 | 376 | 39 | 9 | 5 | 4 | 21 | 1 |
| Guard Banding | Contrived samples | 10 | 375 | 20 | 17 | 12 | 12 | 1 | 1 |
| Guard Banding with updated LC input^3^ | Contrived samples | 7 | 105 | 22 | 16 | 11 | 6 | 1 | 1 |
| Clinical validation for detection of *EGFR* exon 19 deletions  and L858R  alterations: non- inferiority study^2^ | Lung cancer | 177 | N/A | 1 | 5 | 7 | N/A | N/A | N/A |
| Clinical validation study for detection of deleterious alterations in *BRCA1* and *BRCA2*  in prostate cancer^2^ | Prostate cancer | 199 | N/A | 2 | 44 | 55 | 8 | 0 | 1 |
| Clinical validation study for the detection of deleterious alterations in *BRCA1* and *BRCA2*  in prostate cancer | Prostate Carcinoma | 396 | N/A | 2 | 44 | 77 | 7 | N/A | 1 |
| Clinical validation study for detection of *PIK3CA* mutations in breast cancer^2^ | Breast | 359 | N/A | 1 | 28 | 4 | 0 | 0 | 0 |
| Clinical validation study for *ALK* rearrangements in  NSCLC^2^ | Lung cancer | 249 | N/A | 1 | 13 | 1 | 11 | 1 | 0 |
| **Study Title** | **Cancer Types Represented** | **# Unique Samples** | **# of**  **Sample**  **Replicates** | **# of Unique Genes** | **# of Unique** | | | | |
|  |  |  |  |  | **Subs** | **Indels** | **Rearrang.** | **Copy**  **Number**  **Amplif.** | **Copy**  **Number**  **Losses** |
| Clinical validation study for *BRCA1,*  *BRCA2*, and *ATM*  alterations in prostate cancer^2^ | Prostate cancer | 333 | N/A | 3 | 48 | 75 | 10 | 0 | 1 |
| Clinical validation study for detection of SNVs and indels that lead to *MET* exon 14 skipping^2^ | Lung Cancer | 1712 | N/A | 1 | 10 | 22 | N/A | N/A | N/A |
| Clinical validation study for detection of SNVs and indels that lead to *MET* exon 14 skipping^2^ | Lung Cancer | 241 | N/A | 1 | 11 | 45 | N/A | N/A | N/A |
| Clinical validation study for detection of rearrangements that lead to *NTRK* fusions^2^ | Solid Tumor | 203 | N/A | 14 | N/A | N/A | 12 | N/A | N/A |
| Clinical validation study for detection of rearrangements that lead to *ROS1* fusions^2^ | Lung Cancer | 203 | N/A | 8 | N/A | N/A | 7 | N/A | N/A |
| Clinical validation study for detection of  *BRAF* V600E in  CRC | Colorectal Cancer | 433 | N/A | 1 | 1 | 0 | 0 | 0 | 0 |
| Clinical validation study for detection  *BRAF* V600E in  NSCLC | Lung Cancer | 218 | N/A | 1 | 1 | N/A | N/A | N/A | N/A |
| Clinical validation study for detection of *PIK3CA* short variants in breast cancer | Breast Cancer | 268 | N/A | 1 | 21 | N/A | N/A | N/A | N/A |
| Blood Collection Tube Equivalence | Ovarian cancer  Breast cancer  Colorectal cancer  Prostate cancer  Lung cancer  Skin cancer  Stomach cancer | 60 | 192 | 116 | 135 | 39 | 13 | 5 | 0 |
| Automation Line Equivalence | Contrived samples | 8 | 187 | 303 | 1926 | 337 | 63 | 61 | 4 |
| Updated LC Method  Comparison Study^3^ | 10 cancer types | 81 | 324 | 338 | 4220 | 364 | 148 | 116 | 2 |
| Variant Report Curation | Breast cancer  Colorectal cancer  Lung cancer  Prostate cancer  Skin cancer | 19 | 57 | 183 | 300 | 104 | 15 | 11 | 2 |
| Pan-tumor performance  (includes historical analysis) | 20 cancer types | 19868 | N/A | N/A | N/A | N/A | N/A | N/A | N/A |

| **Study Title** | **Cancer Types Represented** | **# Unique Samples** | **# of**  **Sample**  **Replicates** | **# of Unique Genes** | **# of Unique** | | | | |
| --- | --- | --- | --- | --- | --- | --- | --- | --- | --- |
|  |  |  |  |  | **Subs** | **Indels** | **Rearrang.** | **Copy**  **Number**  **Amplif.** | **Copy**  **Number**  **Losses** |
| Molecular Index  Barcode  Performance | 25 cancer types  Contrived samples | 7637 | N/A | 324 | N/A | N/A | N/A | N/A | N/A |
| FoundationOne  Liquid LDT to  FoundationOne  Liquid CDx  Concordance | 25 cancer types | 927 | N/A | 73 | 1815 | 376 | 109 | 46 | N/A |
| FoundationOne  Liquid CDx to  Validated cfDNA  NGS Assay Concordance: *MET* exon 14 (Primary  Analysis) | Lung Cancer | 172 | N/A | 1 | 11 | 21 | N/A | N/A | N/A |
| FoundationOne  Liquid CDx to  Validated cfDNA  NGS Assay  Concordance: *NTRK*  fusions^4^ | Solid Tumor | 116 | N/A | 5 | N/A | N/A | 4 | N/A | N/A |
| Precision and LoD  Confirmation of  *NTRK* Gene Fusions in a Pan-tumor  Setting^4^ | Solid Tumor | 4 | 93 | 6 | N/A | N/A | 4 | N/A | N/A |
| FoundationOne  Liquid CDx to  Validated cfDNA  NGS Assay  Concordance: *EGFR* exon 20 insertions | Lung Cancer | 151 | N/A | 1 | N/A | 25 | N/A | N/A | N/A |
| Precision and LoD  Confirmation of *EGFR* exon 20  insertions | Lung Cancer | 3 | 72 | 1 | N/A | 3 | N/A | N/A | N/A |
| FoundationOne  Liquid CDx to  Validated cfDNA  NGS Assay  Concordance: *BRAF*  V600E | Colorectal Cancer  NSCLC | 304 | 608 | 1 | 1 | N/A | N/A | N/A | N/A |
| Precision and  Confirmation of LoD of *BRAF* V600E | Colorectal Cancer | 1 | 24 | 1 | 1 | N/A | N/A | N/A | N/A |
| Precision and  Confirmation of LoD of *BRAF* V600E | NSCLC | 1 | 22 | 1 | 1 | N/A | N/A | N/A | N/A |

^1^Variants detected may include variants classified as VUS and benign.

^2^Clinical validation study was conducted using the original LC input range for FoundationOne Liquid CDx (30ng-80ng, with conditional processing of samples between 20-30ng)

^3^Study was conducted to validate the new LC input range for FoundationOne Liquid CDx (20ng-60ng). LDT = Laboratory Developed Test

##### 9.1 Concordance – Comparison to an Orthogonal cfDNA NGS Method #1

The detection of short variants and rearrangements by the FoundationOne Liquid CDx assay was compared to that of an externally validated cfDNA next generation sequencing (NGS) assay in 74 genes common to both assays across 278 samples that represented an array of tumor types (>50 unique disease ontologies across 23 cancer types). The cancer types (# samples) included lung [NSCLC (75) and other (3)]; breast (54); prostate (32); colorectal [colon (27) and rectal (6)]; liver (11); ovarian (6); pancreas (9); gastrointestinal (7); bile duct (2); esophageal (5); skin (6); cervical (1); anal (1); bladder (1); gallbladder (1); salivary gland (2); thymus (1); thyroid (3); uterine (2); fallopian tube (1); head and neck (1); soft tissue (1); and unknown primary (19). The study included samples selected from clinical FoundationOne Liquid testing (n=268) and contrived samples consisting of fragmented gDNA diluted in clinical cfDNA to represent rare alterations (n=10).

Using the externally validated NGS assay as the comparator, the analysis demonstrated a short variant positive percent agreement (PPA) of 96.2% with a 95% two-sided confidence interval (CI) of [94.8%- 97.4%]. The short variant negative percent agreement (NPA) was >99.9% with a 95% two-sided CI of [99.9%-100.0%]. The respective PPA of base substitutions and indels with a 95% two-sided CI was 96.1% [94.6%-97.3%] and 100.0% [85.2%-100.0%]. The respective NPA and 95% two-sided CI of base substitutions and indels was >99.9% [99.9%-100.0%] and 100.0% [99.89%-100.0%] (**Table 5**)**.**

Table 5. Concordance of short variants called in FoundationOne Liquid CDx and the cfDNA comparator assay (n= 902 positive variants, n= 152,832 negative variants* by the comparator assay

| **Variant Type** | **FoundationOne**  **Liquid CDx(+)**  **Comparator(+)** | **FoundationOne**  **Liquid CDx(**  **-**  **)**    **Comparator(+)** | **FoundationOne**  **Liquid CDx(+)**  **Comparator(**  **-**  **)** | **FoundationOne**  **Liquid CDx(**  **-**  **)**    **Comparator(**  **-**  **)** | **PPA**  **[95% CI]** | **NPA**  **[95% CI]** | **OPA**  **[95% CI]** |
| --- | --- | --- | --- | --- | --- | --- | --- |
| **All Short Variants** | 868 | 34 | 8 | 152824 | 96.2%  [94.8%-97.4%] | >99.9%  [99.9%-100.0%] | >99.9%  [99.9%-100.0%] |
| **Base**  **Substitutions** | 845 | 34 | 8 | 149511 | 96.1%  [94.6%-97.3%] | >99.9%  [99.9%-100.0%] | >99.9%  [99.9%-100.0%] |
| **Indels** | 23 | 0 | 0 | 3313 | 100.0%  [85.2%- 100.0%] | 100.0%  [99.9%- 100.0%] | 100.0%  [99.9%- 100.0%] |

* Variants detected include variants classified as VUS and benign.

For the concordance of rearrangement detection between FoundationOne Liquid CDx and the comparator assay, the observed rearrangement PPA was 100.0%, with a 95% two-sided CI of [59.0%- 100.0%]. The NPA was 99.8%, with a 95% two-sided CI [99.5%-100.0%] (**Table 6**).

Table 6. Concordance of rearrangements called in FoundationOne Liquid CDx and the cfDNA comparator assay (n= 7 positive, n=1685 negative* as determined by the comparator assay)

|  | **Comparator (+)** | **Comparator (-)** | **Total** |
| --- | --- | --- | --- |
| **FoundationOne Liquid CDx (+)** | 7 | 3 | 10 |
| **FoundationOne Liquid CDx (-)** | 0 | 1682 | 1682 |
| **Total** | 7 | 1685 | 1692 |
|  | **PPA:**  **100.0%**  **[59.0% - 100.0%]** | **NPA:**  **99.8%**  **[99.5% - 100.0%]** | **OPA:**  **99.8%**  **[99.5% - 100.0%]** |

* Variants detected include variants classified as VUS and benign.

Assessment of a subset of highly-actionable alterations were compared between the two assays. The analysis resulted in a PPA of 100% across all eligible highly-actionable alterations called in the comparator assay (**Table 7**).

Table 7. Concordance of CDx alterations called between FoundationOne Liquid CDx and the comparator assay (n = 78)

| **Targeted Alteration** | ***n*** | **PPA [95% CI]** | **NPA [95% CI]** | **PPV [95% CI]** | **NPV^1^ [95% CI]** |
| --- | --- | --- | --- | --- | --- |
| *ALK* rearrangements* | 1 | 100% [2.5%-100%] | 99.9%  [99.7%-100%] | 50% [1.3%-98.7%] | 100% [99.3%-100%] |
| *BRCA1* short variants* | 1 | 100% [2.5%-100%] | 100% [98.7%-100%] | 100% [2.5%-100%] | 100% [98.7%-100%] |
| *BRCA2* short variants* | 2 | 100% [15.8%-100%] | 100% [99.3%-100%] | 100% [15.8%-100%] | 100% [99.3%-100%] |
| *EGFR* exon 19 deletions* | 11 | 100% [71.5%-100%] | 100% [99.7%-100%] | 100% [71.5%-100%] | 100% [99.7%-100%] |
| *EGFR* L858R* | 10 | 100% [69.2%-100%] | 100% [98.7%-100%] | 100% [69.2%-100%] | 100% [98.7%-100%] |
| *NTRK1* rearrangements*** | 3 | 100% [29.2%-100%] | 100% [99.8%-100%] | 100% [29.2%-100%] | 100% [99.3%-100%] |
| *PIK3CA* base substitutions* | 49 | 100% [92.7%-100%] | 100% [99.9%-100%] | 100% [92.7%-100%] | 100% [99.9%-100%] |
| *ROS1* rearrangements* | 1 | 100% [20.7%-100%] | 99.6%  [98.0%-99.9%] | 50% [9.5%-90.6%] | 100% [98.6%-100%] |

*The PPA and NPA for these alterations are unadjusted

^1^Negative Predictive Value (NPV)

These data demonstrate that the FoundationOne Liquid CDx assay and an externally-validated NGS assay are highly concordant across the 76 genes common between the two panels.

##### 9.2 Concordance – FoundationOne Liquid CDx to validated NGS tumor tissue assay (BRCA1 and BRCA2 alterations)

Samples from a total of 279 prostate and ovarian cancer patients were tested and the concordance evaluated between FoundationOne Liquid CDx and the validated NGS tumor tissue assay for the detection of deleterious alterations in *BRCA1* or *BRCA2*. As summarized below, a PPA of 88.03% and an NPA of 95.68% were observed on a sample level (**Table 8**). As summarized in **Table 9** an overall PPA of 87.28% and an NPA of 99.83% were observed at the variant level. Some discordance is expected based on biological differences and sampling times between tumor tissue and plasma samples. Considering the impact of biological differences between analytes, these data demonstrate a high concordance between FoundationOne Liquid CDx and the validated NGS tumor tissue assay for the detection of deleterious alterations in *BRCA1* or *BRCA2*.

Table 8. Concordance (by sample) of FoundationOne Liquid CDx and validated NGS tumor tissue assay in prostate and ovarian cancer patients for the detection of alterations in BRCA1 or BRCA2

|  |  | **NGS Tumor Tissue Assay** | |
| --- | --- | --- | --- |
|  |  | **Positive** | **Negative** |
| **FoundationOne Liquid CDx** | **Positive** | 103 | 7 |
|  | **Negative** | 14 | 155 |
|  |  | **PPA:** 88.03% [80.91%-92.74%] | **NPA:** 95.68% [91.35%-97.89%] |

Table 9. Concordance (by variant) of FoundationOne Liquid CDx and validated NGS tumor tissue assay in prostate and ovarian cancer patients for the detection of alterations in BRCA1 or BRCA2

|  | **F1LCDx+ /Tissue+** | **F1LCDx- /Tissue+** | **F1LCDx+**  **/Tissue-** | **F1LCDx-/ Tissue-** | **PPA (95% CI)** | **NPA (95% CI)** |
| --- | --- | --- | --- | --- | --- | --- |
| **Substitutions** | 77 | 6 | 29 | 20255 | 92.77%  (85.11%, 96.64%) | 99.86%  (99.79%, 99.90%) |
| **Indels** | 65 | 3 | 31 | 16362 | 95.59%  (87.81%, 98.49%) | 99.81%  (99.73%, 99.87%) |
| **Rearrangements** | 4 | 3 | 7 | 1939 | 57.14%  (25.05%, 84.18%) | 99.64%  (99.26%, 99.83%) |
| **Copy number loss** | 5 | 10 | 1 | 263 | 33.33%  (15.18%, 58.29%) | 99.62%  (97.89%, 99.93%) |
| **Total** | 151 | 22 | 68 | 38819 | 87.28%  (81.50%, 91.45%) | 99.83%  (99.78%, 99.86%) |

##### 9.3 Concordance – Comparison to an Orthogonal cfDNA NGS Method #2

The accuracy of using FoundationOne Liquid CDx as a companion diagnostic to identify breast cancer patients harboring *PIK3CA* alterations was assessed with residual plasma samples from the SOLAR-1 clinical trial. Of the remaining plasma samples, 542 were evaluable by the externally-validated NGS method and produced valid results. 418 were evaluable by FoundationOne Liquid CDx, of which 192 positive variants were detected across 188 patients, with 4 patients possessing 2 positive variants each. The distribution of counts per positive variant is listed in **Table 10**.

**Table 10. Distribution of variants detected with FoundationOne Liquid CDx evaluable samples.**

| **Protein Effect in *PIK3CA*** | **# Variant Calls**  **(188 Positive Samples)** |
| --- | --- |
| C420R | 3 |
| E542K | 25 |
| E545A | 1 |
| E545G | 2 |
| E545K | 50 |
| H1047L | 9 |
| H1047R | 100 |
| H1047Y | 1 |
| Q546R | 1 |
| **Total** | **192** |

A total of 412 valid samples generated valid results with both assays. The primary analysis using NGS Method #2 as the reference assay achieved a PPA [95% CI] of 97.06% [93.27%, 99.04%], and an NPA [95% CI] of 91.74% [87.52%, 94.88%]. The contingency table for this comparison is provided in **Table** **11**, with counts representing number of samples (versus number of variant calls).

The sample counts in the core 2x2 white boxes total to 412 samples. There were seven samples evaluable with FoundationOne Liquid CDx but failed (italicized in **Table 11**), as well as three samples missing from reference assay data. There were five samples unevaluable by the reference assay; three of these aligned with the 418 evaluable FoundationOne Liquid CDx samples, while two were among the 130 samples not evaluable due to insufficient plasma.

**Table 11. Contingency table comparing FoundationOne Liquid CDx with the reference assay, primary analysis with 412 cases.**

|  | |  | **Reference Assay** | |  |  |  |
| --- | --- | --- | --- | --- | --- | --- | --- |
|  |  | **Positive** | **Negative** | **Not Evaluable** | **Missing** | **Total** |  |
| **FoundationOne**  **Liquid CDx** | **Positive** | 165 | 20 | 2 | 1 | **188** | PPA_F1L:_ 89.19% [83.80%, 93.27%] |
|  | **Negative** | 5 | 222 | 1 | 2 | **230** | NPA_F1L_: 97.80% [94.93%-99.28%] |
|  | **Evaluable but Failed** | 0 | *7* | 0 | 0 | 7 |  |
|  | **Not Evaluable** | 35 | 93 | 2 | 0 | 130 |  |
|  | **Total** | 205 | 342 | 5 | 3 | 555 |  |
|  | | PPA_ONC_: 97.06% [93.27%, 99.04%] | NPA_ONC_: 91.74% [87.52%, 94.88%] |  |  |  | OPA: 93.93% [91.17%, 96.04%] |

##### 9.4 Concordance – FoundationOne Liquid CDx to an externally validated cfDNA NGS assay (SNVs and indels that lead to MET exon 14 skipping)

An analytical accuracy study was performed to demonstrate the concordance between FoundationOne Liquid CDx and an externally validated cfDNA NGS comparator (evNGS) assay for the detection of SNVs and indels that lead to *MET* exon 14 skipping. Overall, there were 74 overlapping genes targeted by the 2 assays and the comparator assay bait set covered the same regions as the FoundationOne Liquid CDx bait set.

The analytical accuracy study was conducted with 45 samples from the clinical bridging study with 41 samples from patients enrolled in the GEOMETRY-mono 1 trial (refer to Section 10.7 below). An additional 100 NSCLC samples were sourced from Foundation Medicine’s clinical archives, 38 samples from NSCLC patients previously evaluated in the accuracy study to support the original PMA P190032 (refer to section 9.1 above) and 31 externally sourced plasma samples from NSCLC cases whose tissue specimens tested positive for *MET* exon 14 skipping alterations and were subsequently tested with FoundationOne Liquid CDx to determine their *MET* exon 14 skipping associated alteration status prior to conducting the accuracy study statistical analysis. Samples selected from Foundation Medicine’s clinical archives that were positive for *MET* exon 14 skipping alterations had to have a variant allele frequency (VAF) greater than or equal 0.40%.

Of the 214 samples, 179 samples had DNA yield that allowed processing with FoundationOne Liquid CDx at the specified LC DNA input of 30ng-80ng. Thirty-five (35) samples were tested with FoundationOne Liquid CDx at a lower LC DNA input of out of specification of 20ng-<30ng LC DNA input. Of the 179 samples that had sufficient DNA yield for testing with FoundationOne Liquid CDx, 3 samples had a FoundationOne Liquid CDx sequence analysis Quality Control (QC) failure, while 4 had an evNGS QC failure.

The primary analytical concordance analysis, using the evNGS assay results as the reference, included 172 samples that passed QC with both assays. Forty-eight (48) of the 172 samples were identified as positive for *MET* exon 14 skipping alterations by FoundationOne Liquid CDx. The statistical analysis using the evNGS assay results as the reference showed a PPA of 94.87% with 95% CI (83.11%-

98.58%), a NPA of 91.83% with 95% CI (85.80%, 95.32%), a PPV of 77.08% with 95% CI (63.46%,

86.69%) and a negative predictive value (NPV) of 98.39% with 95% CI (94.31%, 99.56%) as shown in **Table 12**. Since the samples were selected from different sources based on different assays, the unadjusted PPA/NPA and unadjusted PPV/NPV in **Table 12** may be subject to potential bias.

Table 12. Primary Concordance Analysis Comparing Sample-level Biomarker Detection between FoundationOne Liquid CDx and Comparator Assay

|  |  |  | **evNGS** |  |  |
| --- | --- | --- | --- | --- | --- |
|  |  | ***MET* ex14 positive** | ***MET* ex14 negative** | **Total** | **PPV/NPV (95%**  **CI)** |
| **F1LCDx** | ***MET* ex14 positive** | 37 | 11 | 48 | PPV: 77.08% (63.46%, 86.69%) |
|  | ***MET* ex14 Negative** | 2 | 122 | 124 | NPV: 98.39% (94.31%, 99.56%) |
|  | **Total** | 39 | 133 | 172 |  |
|  | **PPA/NPA (95% CI)** | PPA: 94.87% (83.11%, 98.58%) | NPA: 91.83% (85.80%, 95.32%) |  |  |

Ten (10) of the eleven (11) samples that were F1LCDx-positive/evNGS-negative [F1LCDx(+)/evNGS()] were discordant due to differences in variant reporting by assays. Of the 11 samples, 10 samples harbored *MET* exon 14 deletions ≥6bp detectable by the evNGS variant caller, which calls variants in the evNGS’s loci of interest (LOI) and indels ≥6bp in *MET* exon 14. Since *MET* ex14 indels ≥6bp are not part of the evNGS’s LOI, this variant type is filtered out and not reported by the evNGS’s analysis software in the default setting, and thus are considered negatives by the evNGS comparator assay. Further the remaining one (1) sample from the 11 samples that were F1LCDx(+)/evNGS(-), contained a *MET* exon 14 deletion <6bp which cannot be called with the evNGS variant because the variant caller can only output *MET* exon 14 deletions ≥6bp. The evNGS reporting rules only correspond to biomarker rule category 3, so all 37 samples that were F1LCDx(+)/evNGS(+) had *MET* exon 14 skipping alterations that correspond to biomarker rule category 3, i.e., these samples had base substitutions and indels affecting positions 0, +1, +2, or +3 at the splice donor site of the 3′ boundary of *MET* exon 14. The evNGS assay does not call category 1 and 2 biomarkers as they are not included in their LOI. In the two discordant samples that were F1LCDx-negative(-)/evNGS(+), base substitutions reported by the evNGS were not detected in the variant analysis pipeline of FoundationOne Liquid CDx.

Four of the eleven discordant samples that were F1LCDx(+)/evNGS(-) were from patients evaluated in the clinical therapeutic study for whom efficacy data was available. Of these 4 patients, 3 had partial response to TABRECTA, while one had progressive disease. Although these patients had discordant results, these results appear to suggest that these patient with F1LCDx(+)/evNGS(-) were *MET* exon 14 deletion positive.

##### 9.5 Concordance – FoundationOne Liquid CDx to an externally validated cfDNA NGS assay

###### (NTRK1/2/3 Fusions)

An analytical accuracy study was performed to demonstrate the concordance between FoundationOne Liquid CDx and an externally validated cfDNA NGS comparator assay for the detection of *NTRK* fusions. For this study, seven (7) residual cfDNA samples were selected from patients enrolled in the STARTRK-2 trial used to support the effectiveness of the device, seven (7) residual cfDNA clinical samples were externally sourced, and 102 residual cfDNA samples were sourced from Foundation

Medicine’s clinical archives. Overall, a total of 116 sample replicates were processed using FoundationOne Liquid CDx in this study. Of the 116 samples, 113 were processed with the evNGS. Of the 113 samples run by both assays for this study, one (1) sample had a FoundationOne Liquid CDx post-sequencing QC failure, while 10 had an evNGS post-sequencing QC failure.

Measures of analytical concordance for the 102 samples that passed QC with both assays were determined. Since specimens were selected based on FoundationOne Liquid CDx and confirmed by the evNGS agreement, PPV and NPV are estimated conditional on FoundationOne Liquid CDx. PPV was estimated as 40% (4/10) with two-sided 95% CI (16.8%, 68.7%), and NPV as 100% (92/92) with two-sided 95% CI (95.99%, 100.00%), as shown in **Table 13**, below. For informational purposes, unadjusted PPA and NPA are also displayed.

Table 13. Concordance Analysis Comparing Sample-level Biomarker Detection between FoundationOne Liquid CDx and evNGS

|  |  |  | **evNGS** | |  |  |
| --- | --- | --- | --- | --- | --- | --- |
|  |  | ***NTRK1/2/3* fusion positive** | ***NTRK1/2/3* fusion negative** |  | **Total** | **PPV/NPV (95% CI)** |
| **F1LCDx** | ***NTRK1/2/3* fusion** | 4 | 62 |  | 10 | PPV: 40.0%  (16.8%,  68.7%) |
|  | **positive^1^** |  |  |  |  |  |
|  | ***NTRK1/2/3* fusion negative** | 0 | 92 |  | 92 | NPV: 100%  (95.99%,  100%) |
|  | **Total** | 4 | 98 |  | 102 |  |
|  | **PPA/NPA** | PPA: 100% | NPA: 93.9% |  |  |  |
|  | **(Unadjusted)** | (51.01%, | (87.3%, |  |  |  |
|  | **(95% CI)** | 100%) | 97.2%) |  |  |  |

^1^No *NTRK2* fusion positive samples were evaluated in this study

^2^These six samples were discordant due to the fusion breakpoints falling in regions that the evNGS assay does not bait for.

The six (6) samples that were *NTRK1/2/3* fusion positive by FoundationOne Liquid CDx and *NTRK1/2/3* fusion negative by the evNGS were discordant due to the fusion breakpoints falling in regions that the evNGS assay does not bait for. Specifically, the evNGS assay did not claim to generate coverage in certain regions of interest (e.g., intron 8 of *NTRK1* and intron 5 of *ETV6*), and thus were negative by the evNGS comparator assay.

##### 9.6 Concordance – FoundationOne Liquid CDx to an externally validated cfDNA NGS assay (EGFR exon 20 insertions)

An analytical accuracy study was performed to demonstrate the concordance between FoundationOne Liquid CDx and an externally validated cfDNA NGS comparator assay for the detection of *EGFR* exon 20 insertions. For this study, 101 frozen plasma samples were identified from patients enrolled in a clinical therapeutic study and 125 residual cfDNA samples were sourced from Foundation Medicine’s clinical archives. Of the 125 residual cfDNA samples, four (4) were excluded due to diluted DNA concentration being out of acceptable range or evNGS post-sequencing QC failure. Of the 101 frozen plasma samples, 71 were excluded from the analysis due to insufficient cfDNA yield, diluted DNA concentration being out of acceptable range, or evNGS post-sequencing QC failure. Overall, a total of 151 samples from NSCLC patients were processed using both FoundationOne Liquid CDx and an externally validated cfDNA NGS assay in this study.

Analytical concordance was determined for the 151 samples that passed QC with both assays. Since specimens were selected based on FoundationOne Liquid CDx and confirmed by the evNGS assay, PPV and NPV are estimated conditional on FoundationOne Liquid CDx. Forty-nine (49) of the 151 samples were identified as positive for *EGFR* exon 20 insertions by both FoundationOne Liquid CDx and evNGS. The statistical analysis showed a PPV of 100% with two-sided 95% CI [92.70%-100%] and a NPV of 99.02% with two-sided 95% CI [94.65%-99.83%], as shown in

***Table* *14*** below.

Table 14. Concordance Analysis Comparing Sample-Level Biomarker Detection Between FoundationOne Liquid CDx and evNGS

|  |  |  | **evNGS** |  |  |
| --- | --- | --- | --- | --- | --- |
|  |  | ***EGFR* exon 20**  **insertion positive** | ***EGFR* exon 20 insertion negative** | **Total** | **PPV/NPV**  **(95% CI^1^)** |
| **F1LCDx** | ***EGFR* exon 20**  **insertion positive** | 49 | 0 | 49 | PPV: 100% (92.70%, 100%) |
|  | ***EGFR* exon 20 insertion negative** | 1 | 101 | 102 | NPV: 99.02% (94.65%, 99.83%) |
|  | **Total** | 50 | 101 | 151 |  |
|  | **PPA/NPA (Unadjusted) (95% CI^1^)** | PPA: 98.00% (89.50%, 99.65%) | NPA: 100% (96.34%, 100%) |  |  |

In the one (1) discordant sample that was F1LCDx-negative/evNGS-positive, a 3 bp *EGFR* exon 20 insertion reported by the evNGS was not detected in the variant analysis pipeline of FoundationOne Liquid CDx.

##### 9.7 Concordance – FoundationOne Liquid CDx to an externally validated ctDNA NGS assay (BRAF V600E)

An analytical accuracy study was performed to demonstrate the concordance between FoundationOne Liquid CDx and an externally validated ctDNA NGS (evNGS) comparator assay for the detection of *BRAF* V600E. Overall, a total of 304 samples from CRC (n=189) and NSCLC (n=115) patients were processed using both FoundationOne Liquid CDx and an externally validated ctDNA NGS assay in this study.

Analytical concordance using the evNGS assay results as the reference for the 304 samples that passed QC with both assays was determined. Since archived specimens were selected based on previous FoundationOne Liquid CDx or FoundationOne Liquid results and tested again by the evNGS assay and FoundationOne Liquid CDx, calculation of PPA and NPA is presented adjusted for the enrichment of *BRAF* V600E positives in the concordance evaluation sample cohort. Ninety-one (91) of the 304 samples were identified as positive for *BRAF* V600E by both FoundationOne Liquid CDx and the evNGS. Adjusted PPA has a point estimate of 98.91% with two-sided 95% CI [94.10%-99.81%]. Adjusted NPA has a point estimate of 100.00% with a 95% two-sided CI of [98.22%-100.00%]. For informational purposes, unadjusted PPA, NPA, PPV and NPV are also displayed, as shown in **Table 15**, below.

Table 15. Contingency Table Comparing the Detection of BRAF V600E by the FoundationOne Liquid CDx and Externally Validated ctDNA Assay

|  | | **evNGS** | | |  |
| --- | --- | --- | --- | --- | --- |
|  |  | ***BRAF* V600E positive** | ***BRAF* V600E negative** | **Tota l** | **PPV/NPV**  **(Unadjusted) (95% CI)** |
| **F1LCDx** | ***BRAF* V600E positive** | 91 | 0 | 91 | PPV: 100% (95.95%, 100%) |
|  | ***BRAF* V600E negative** | 1* | 212 | 213 | NPV: 9.53% (97.39%, 99.92%) |
|  | **Total** | 92 | 212 | 304 |  |
|  | **PPA/NPA**  **(Unadjusted) (95% CI)** | PPA: 98.91% (94.10%, 99.81%) | NPA: 100% (98.22%, 100%) |  |  |

**This discordant sample had very low supporting reads and variant allele frequency in FoundationOne Liquid CDx, which did not pass FoundationOne Liquid CDx calling threshold.*

##### 9.8 Limit of Detection (Analytical Sensitivity)

The LoD for each variant type was established by processing a total of 1,069 sample replicates across ten contrived (enzymatically fragmented cell-line gDNA) samples representing short variants, rearrangements, and copy number alterations. The LoD was determined using the conservative hit rate approach for the majority of variants. A probit model was used when appropriate (when ≥3 dilution levels with hit rates between 10% and 90% were observed). LoD by hit rate was defined as the mean variant allele frequency (VAF) value (for short variants and rearrangements) or mean tumor fraction (TF) value (for copy number alterations) at the lowest dilution level tested with at least 95% detection across replicates. The hit rate was computed as the number of replicates with positive variant calls per the total number of replicates tested at each level of the targeted VAF (short variants and rearrangements) or tumor fraction (copy number alterations). Short variants with hit rates of at least 95% at all dilution levels or hit rates below 95% for all dilution levels were excluded from analysis as LoD could not be reliably estimated.

Confirmed LoDs for CDx alterations are presented below in **Table 16** and are taken from the confirmation of LoD studies as presented in Section 9.13. The confirmation of LoD studies utilized clinical samples assessed near the established LoD (targeting 1x-1.5x LoD). The confirmed LoD for targeted short variants, rearrangements, and copy number alterations demonstrate at least a 95% hit rate at a level near the established LoD (**Table 17**).

Table 16. Established and Confirmed LoD for CDx alterations

| **Tumor Type** | **Gene/variant** | **Alteration Subtype** | **Established LoD** | **Confirmed LoD (Fold LoD)** |
| --- | --- | --- | --- | --- |
| *Breast cancer* | *PIK3CA* | Substitutions | 0.34% VAF | 0.39% VAF (1.14x) |
| *CRC* | *BRAF* V600E | Substitution | 0.33% VAF | 0.70% VAF (2.12x) |
| *NSCLC* | *ALK* | Rearrangement | 0.24% VAF | 0.68% VAF (2.84x) |
|  | *BRAF* V600E | Substitution | 0.33% VAF | 0.86% VAF(2.61x) |
|  | *EGFR* | Substitutions (L858R) | 0.34% VAF | 0.64% VAF (1.90x) |
|  |  | Indels (exon 19 deletions) | 0.27% VAF | 0.45% VAF (1.65x) |
|  | *MET* | Substitutions (exon 14) | Not Determined | 0.40% VAF (N/A)^^[[1]](#footnote-1)^^ |
|  |  | Indels (exon 14) | 0.41% VAF | 0.28% VAF (0.67x) |
|  | *ROS1* | Fusion | 0.52% VAF | 1.30% VAF (2.51x) |
| *Prostate cancer* | *ATM* | Substitutions | 0.51% VAF | 0.56% VAF (1.09x) |
|  |  | Indels | 0.51% VAF | 0.86% VAF (1.68x) |
|  |  | Rearrangement | Not Determined | 1.13% VAF (N/A)^1^ |
|  | *BRCA1* | Substitutions | 0.34% VAF | 0.51% VAF (1.49x) |
|  |  | Indels | 0.38% VAF | 0.55% VAF (1.44x) |
|  |  | Rearrangement | Not Determined | 0.87% VAF (N/A)^1,^ ^^[[2]](#footnote-2)^^ |
|  | *BRCA2* | Substitutions | Not Determined | 0.71% VAF (N/A)^1^ |
|  |  | Indels | 0.36% VAF | 0.63% VAF (1.74x) |
|  |  | Rearrangement | Not Determined | 0.48% VAF (N/A)^1,^ ^3^ |
|  |  | Copy Number Loss | 48.1% TF^4^ | N/A |
| *Solid tumors* | *NTRK1* | Fusion | 0.44% VAF | 0.75% VAF (1.70x) |
|  | *NTRK3* | Fusion | 0.27% VAF | 0.68% VAF (2.52x) |

^3^Confirmed LoD for BRCA2 RE was using the DIBv1 primer set. LoD was also confirmed using the DIBv2 primer set at 1.49% VAF. ^4^LoD was established in a clinical sample and therefore confirmation of LoD was not applicable.

The platform LoD for short variants, rearrangements, and copy number alterations are presented in **Table 17.** A total of 864 short variants were included in the platform LoD analysis. The enhanced sensitivity region of the bait set contains 269 of the short variants analyzed and the standard sensitivity region of the bait set contains 595 of the short variants analyzed. The estimated LoD for short variants is 0.40% for the enhanced sensitivity region and 0.82% of the standard sensitivity region. The median LoD is 30.4% tumor fraction for copy number losses.

Because a major component driving the detectability of a variant is genomic context (repetitiveness of the reference genomic region), the LoD analysis by alteration subtype was also evaluated within categories based on genomic context as summarized in **Table 17**.

Table 17. LoD by variant subtype based on genomic context

| **Region** | **Alteration Subtype** | **LoD**  **Unit** | **N** | **Minimum LoD** | **1st Quantile LoD** | **Median LoD** | **3rd Quantile LoD** |
| --- | --- | --- | --- | --- | --- | --- | --- |
| Enhanced  Sensitivity Region | **Short Variants: Enhanced Sensitivity Region Total** | VAF | **269** | **0.20%** | **0.33%** | **0.40%** | **0.50%** |
|  | Insertion/Deletion in non- repetitive region or a repetitive region of <=3 base pairs |  | 10 | 0.23% | 0.29% | 0.31% | 0.36% |
|  | Insertion/Deletion in a repetitive region of 4 to 6 base pairs |  | 23 | 0.28% | 0.37% | 0.48% | 0.56% |
|  | Insertion/Deletion in a repetitive region of >=7 base pairs |  | 6 | 0.33% | 0.48% | 0.58% | 0.82% |
|  | Substitution in a non-repetitive region or a repetitive region of <=7 base pairs |  | 229 | 0.20% | 0.33% | 0.39% | 0.49% |
|  | Substitution in a repetitive region of >7 base pairs |  | 1 | 0.32% | 0.32% | 0.32% | 0.32% |
| Standard  Sensitivity Region | **Short Variants: High**  **Sensitivity Region Total** | VAF | **595** | **0.40%** | **0.70%** | **0.82%** | **0.98%** |
|  | Insertion/Deletion in non- repetitive region or a repetitive region of <=3 base pairs |  | 18 | 0.46% | 0.68% | 0.87% | 1.00% |
|  | Insertion/Deletion in a repetitive region of 4 to 6 base pairs |  | 32 | 0.61% | 0.75% | 0.87% | 0.95% |
|  | Insertion/Deletion in a repetitive region of >=7 base pairs |  | 11 | 0.59% | 1.07% | 1.15% | 1.20% |
|  | Substitution in a non- repetitive region or a repetitive region of <=7 base pairs |  | 524 | 0.40% | 0.70% | 0.81% | 0.96% |
|  | Substitution in a repetitive region of >7 base pairs |  | 8 | 0.69% | 0.83% | 0.96% | 1.28% |
| Enhanced  Sensitivity Region | **Rearrangements** | VAF | **7** | **0.20%** | **0.26%** | **0.37%** | **0.47%** |
| Enhanced/  Standard  Sensitivity Region | **Rearrangements** | VAF | **1** | **0.28%** | **0.28%** | **0.28%** | **0.28%** |
| Standard  Sensitivity Region | **Rearrangements** | VAF | **1** | **0.90%** | **0.90%** | **0.90%** | **0.90%** |
| NA | **Copy Number Amplifications** | TF | **8** | **19.8%** | **19.8%** | **21.7%** | **25.2%** |
| NA | **Copy Number Losses** | TF | **2** | **12.70%** | **21.55%** | **30.40%** | **39.25%** |

The median LoD for highly-actionable, non-CDx alterations evaluated for LoD are presented in **Table** **18**. The median LoD for these targeted short variants are consistent with the platform LoD presented in **Table 16.**

Table 18. LoD for non-CDx alterations

| **Gene** | **Alteration Subtype** | **Number of Samples Evaluated** | **Median LoD^1^** |
| --- | --- | --- | --- |
| *BRAF* | Substitutions | 1 | 0.33% VAF |
| *EGFR* | Indels | 3 | 0.65% VAF |
| *ERBB2* | Copy Number Amplification | 1 | 19.8% TF |
| *KRAS* | Substitutions | 2 | 0.33% VAF |
| *MET*2 | Indels | 1 | 0.41% VAF |
| *NRAS* | Substitutions | 2 | 0.42% VAF |
| *PALB2* | Indels | 1 | 0.37% VAF |
|  | Substitutions | 1 | 0.51% VAF |

VAF = variant allele frequency TF = tumor fraction

^1^Quantitative reporting of %VAF/%TF has not been approved by FDA. ^2^This LoD applies to *MET* alterations that do not meet the CDx rules.

##### 9.9 Limit of Blank (LoB)

The study evaluated the limit of blank (LoB) of the F1LCDx assay updated with DIBv2 improvements. Two plasma cfDNA replicates and one replicate of donor-matched gDNA from 44 donors with no known cancer diagnosis, who were categorized in both age and smoking cohorts, were collected, extracted, and tested across 2 reagent lots where 2 cfDNA replicates and 1 matched gDNA replicate from each donor were tested in the same lot. A total of 87 cfDNA sample replicates (prepared from 44 donors x 2 replicates minus 1 replicate with DNA extraction failure) and 44 matched buffy coat gDNA sample replicates (single extraction from each of the 44 donors) were processed with F1LCDx across 2 reagent lots. Out of 132 sample replicates, 131 passed all QC specifications. LoB was assessed by calculating positive call rates for universal variants in technical blank samples simulated by excluding from F1LCDx cfDNA sample data variants identified in the matched donor gDNA F1LCDx data.

However, it should be noted that F1LCDx does not normalize patient samples with their germline DNA. Universal variants are defined as a set of variants detected at least once in any sample processed in F1LCDx validation studies. The LoB study results obtained in the technical blank samples are summarized in **Table 19** below.

Table 19. LoB Study Results in Technical Blanks

| **Variant Category** | **# of detected variants**  **across all source**  **samples**  **(n=44) in a variant level** | **Total # of unique variants for a variant level in the**  **universal set ×**  **Total # of source samples** | **False Positive Rate (%)** | **# of detected variants across all sample replicates**  **(n=87) in a variant category** | **Per Sample**  **Replicate False**  **Positive Rate (%)**  **[number of replicates with at least one detected**  **variant/total sample**  **replicates)** |
| --- | --- | --- | --- | --- | --- |
| Category 1:  *ATM* 1208C>G | 1 | 39,512 | 0.0026 | 1 | 1.15% [1/87] |
| Category 1:  *BRCA* 6116T>G | 1 | 39,512 | 0.0026 | 1 | 1.15% [1/87] |
| Category 1:  All **other*** CDx variants  noted in Table 1 of Intended Use | 0 | 39,512 | 0.0000 | 0 | 0.00% [0/87] |
| Category 2: cfDNA  Biomarkers with Strong  Evidence of Clinical  Significance in cfDNA | 0 | 44 | 0.0000 | 0 | 0.00% [0/87] |
| Category 3: Biomarkers with  Evidence of Clinical Significance in tissue supported by:  3A: strong analytical validation using cfDNA  3B: analytical validation using  cfDNA | 2 | 13,288 | 0.0151 | 3 | 3.45% [3/87] |
| Category 4: Other Biomarkers  with Potential Clinical Significance** | 19 | 314,776 | 0.0060 | 22 | 19.54% [17/87] |

*Note that the two false positives observed in Category 1, identified as ATM 2921+1G>C and BRAF 1799T>A, are excluded from the false positive rate calculation of ‘All other CDx variants’ category.

**False positive results may be due to background signals inherent in sequencing methods designed for high sensitivity, clonal hematopoiesis of indeterminate potential, or germline polymorphisms.

All CDx variants had false positive rate (FPR) < 5% and 2 CDx variants were detected at VAFs below the 0.40% VAF LoD for SNVs and indels in F1LCDx (a single call for *ATM*_1208C>G and a single call for *BRCA*_6116T>G, both at VAF around 0.10%.

Across all four categories reported by F1LCDx, FPR of on a per variant per sample replicate ranged from 1.15% (1 variant call out of 87 valid cfDNA replicates) to 2.30% (2 variant calls out of 87 valid sample replicates) for non-companion diagnostic variants (Categories 2-4 of the tumor profiling variants reported).

No rearrangements or copy number alterations were detected in any of the four categories.

Since a large number of non-CDx SNV and indel variants are detected in technical blanks at low VAF levels (less than 0.5% VAF for SNVs and 1% VAF for indels), i.e., in plasma from donors that do not have solid neoplasms after subtracting the presumably germline variants detected in the donors buffy coat, there is a risk that variants detected at very low allele frequency may be a false positive result. This risk may be due to background signals inherent in sequencing methods designed for high sensitivity. They may also be due to CHIP. Additional clinical investigation to confirm the presence of the variant in the patient’s tumor with another FDA approved or cleared test is strongly recommended.

##### 9.10 Potentially Interfering Substances

To evaluate the robustness of the FoundationOne Liquid CDx results in the presence of potentially interfering exogenous and endogenous substances, a total of 11 potential interferents were evaluated. These potential interferents included six endogenous substances (albumin, conjugated bilirubin, unconjugated bilirubin, cholesterol, hemoglobin and triglycerides) and five exogenous substances (DNA from another source [the microorganism Staphylococcus epidermidis], excess anticoagulant, proteinase K, ethanol and molecular index barcodes).

A total of 340 samples were tested to evaluate the potential interference of these substances. An assessment of the cfDNA yield obtained during the DNA isolation, purification, and quantification steps, as well as at library construction QC and hybrid capture QC was performed. The process success rates for each step are listed in **Table 20**.

Table 20. Process success rates with interfering substances

| **Process** | **# Failed** | **# Pass** | **Total** | **Success Rate (%)** | **95% CI LB (%)** | **95% CI UB (%)** |
| --- | --- | --- | --- | --- | --- | --- |
| DNA  Extraction | 0 | 180 | 180 | 100.00 | 97.97 | 100.00 |
| LC | 1 | 339 | 340 | 99.71 | 98.37 | 99.99 |
| HC | 3 | 336 | 339 | 99.12 | 97.44 | 99.82 |
| Sequencing | 0 | 336 | 336 | 100.00 | 98.91 | 100.00 |

For each potential interferent, concordance of alteration calls was calculated relative to a control sample without interferent. The pre-defined variants included 27 short variants, 17 rearrangements, and 3 copy number variants. Of the 11 potential interferents tested across 16 conditions, concordance for all variant calls was 100% for 8 conditions and ≥97% for all conditions (**Table 21**).

Table 21. Concordance per substance for variants ≥1x LoD

| **Substance** | **Detected Reps** | **Total Reps** | **Concordance** | **95% two-sided exact CI_lower** | **95% two-sided exact CI_upper** |
| --- | --- | --- | --- | --- | --- |
| Triglycerides, 37 mmol/L (or 33 g/L) | 80 | 80 | 100.00% | 95.49% | 100.00% |
| Hemoglobin, 2.0 g/L | 78 | 78 | 100.00% | 95.38% | 100.00% |
| Albumin, 60 g/L | 80 | 82 | 97.56% | 91.47% | 99.7% |
| Bilirubin (conjugated), 0.2 g/L | 84 | 84 | 100.00% | 95.7% | 100.00% |
| Bilirubin (unconjugated), 0.2 g/L | 76 | 78 | 97.44% | 91.04% | 99.69% |
| Cholesterol Level 2, 3.88 mmol (150 mg/dL) | 80 | 82 | 97.56% | 91.47% | 99.7% |
| Cholesterol Level 1, 6.47mmol (250 mg/dL) | 74 | 76 | 97.37% | 90.82% | 99.68% |
| Staphylococcus epidermidis, 1 x 106 CFU/mL | 78 | 78 | 100.00% | 95.38% | 100.00% |
| Anticoagulant, 5X nominal volume | 82 | 82 | 100.00% | 95.6% | 100.00% |
| Proteinase K, +0.6 mg/mL | 98 | 99 | 98.99% | 94.50% | 99.97% |
| Proteinase K, +0.3 mg/mL | 92 | 92 | 100.00% | 96.07% | 100.00% |
| Ethanol, +2.5% | 96 | 98 | 97.96% | 92.82% | 99.75% |
| Ethanol, +5.0% | 94 | 95 | 98.95% | 94.27% | 99.97% |
| Molecular Index barcodes, +5% | 70 | 72 | 97.22% | 90.32% | 99.66% |
| Molecular Index barcodes, +15% | 96 | 96 | 100.00% | 96.23% | 100.00% |
| Molecular Index barcodes, +30% | 98 | 98 | 100.00% | 96.31% | 100.00% |

Taken together, these data indicate that the FoundationOne Liquid CDx assay is robust to potential specimen- related endogenous substances and exogenous contaminants or interferents.

##### 9.11 Hybrid Capture Bait Specificity

Bait specificity was addressed through an assessment of coverage of targeted regions in FoundationOne Liquid CDx using 3,546 validation study samples. Results show that targeted genomic regions have consistently high, uniform coverage. For each genomic region associated with a predefined subset of highly-actionable alterations, between 94% to 100% of samples possessed the expected level of coverage. An in-depth, platform-wide examination of the FoundationOne Liquid CDx baitset through the analysis of HapMap process control samples revealed that, on average, 98.8% and 94.1% of platform-wide baited coding and non-coding regions, respectively, met their expected coverage levels. Samples assessed in this study consistently demonstrated high quality uniform and deep coverage across the entire genomic region targeted by the assay.

##### 9.12 Carryover/Cross-Contamination

The study demonstrated that the risk of cross contamination (intra-plate), and carry-over contamination (inter- plate) of samples during the processing of the FoundationOne Liquid CDx assay is low. A total of 376 wells were examined for intra-plate and inter-plate contamination by processing and sequencing of contrived samples derived from cell lines at high input concentrations with known genomic backgrounds. Unique variants of each cell line were characterized by independent control sequencing runs. The samples were arrayed in a checkerboard fashion across four 96-well PCR plates to detect cross-contamination events. A cross-contamination rate of 0.53% (2/376) was observed in this study. These data demonstrate a low probability of cross contamination during the FoundationOne Liquid CDx process.

##### 9.13 Precision: Reproducibility and Confirmation of LoD

Multiple Precision and Confirmation of LoD studies were performed, using both clinical and contrived samples to evaluate precision and only clinical samples for confirmation of LoD. Precision was evaluated for alterations associated with both CDx claims and tumor profiling. Target alterations were assessed at two target levels each (near LoD and 2-3x LoD) for the contrived samples, and at one level (targeting 1-1.5x LoD) for clinical cfDNA samples.

In all studies, each sample was divided into 24 aliquots, with 12 duplicates being processed on the same plate under the same conditions. Each sample was tested across 24 replicates. Reproducibility was assessed and compared across three lots, two sequencers, and two processing runs. Samples were processed near the assay’s minimum DNA input mass.

The studies evaluate the precision of FoundationOne Liquid CDx for detecting a set of highly actionable variants. **Table 22** and **Table 23** summarize the Disease Ontology (if applicable), Variant Subtype, Targeted Variant, Reproducibility, Observed Average Measurand, and LoD for each sample with CDx variants and non-CDx variants, respectively.

Table 22. Precision and Confirmation of LoD by Targeted CDx Variant

| **Targeted Variant** | **Variant Subtype** | **Cancer Type** | **Reproducibility**  **(%) (95% Two- sided CI)** | **Observed**  **Average**  **Measurand** | **LoD** |
| --- | --- | --- | --- | --- | --- |
| ALK_EML4_fusion | RE | Lung cancer | 100 (86.2, 100) | 0.68% VAF^1^ | 0.24% VAF |
| ALK-EML4 fusion | RE |  | 100 (85.75, 100) | 1.39% VAF^1^ | 0.24% VAF |
| ALK-EML4 fusion | RE | Contrived | 100 (85.75, 100) | 0.64% VAF | 0.24% VAF |
| ALK-EML4 fusion | RE |  | 100 (85.18, 100) | 0.89% VAF | 0.24% VAF |
| ALK-NPM1 fusion | RE |  | 78.26 (56.3, 92.54) | 0.4% VAF | 0.94% VAF |
| ALK-NPM1 fusion | RE |  | 100 (85.75, 100) | 0.64% VAF | 0.94% VAF |
| ATM K1773fs*3 | Indel | Contrived | 100 (85.75, 100) | 0.77% VAF | 0.51% VAF |
| ATM K1773fs*3 | Indel |  | 100 (85.18, 100) | 1.04% VAF | 0.51% VAF |
| ATM I2012fs*4 | Indel | Prostate cancer | 100 (85.18, 100) | 0.86% VAF^1^ | 0.51% VAF |
| ATM splice site 8850+1G>A | Sub | Prostate cancer | 100 (85.75, 100) | 0.56% VAF^1^ | 0.51% VAF |
| ATM-EXPH5 truncation | RE |  | 100 (85.75, 100) | 1.13% VAF^1^ | Not Determined |
| BRAF 1799T>A | Sub | CRC | 100 (86.2, 100) | 0.70% VAF^1^ | 0.33% VAF |
| BRAF 1799T>A | Sub | NSCLC | 100 (85.13, 100) | 0.86% VAF^1^ | 0.33% VAF |
| BRCA N1784fs*3 | Indel | Stomach cancer | 87.5 (69, 95.7) | 0.34% VAF | 0.36% VAF |
| BRCA1 D825fs*21 | Indel | Contrived | 100 (85.75, 100) | 0.61% VAF | 0.38% VAF |
| BRCA1 D825fs*21 | Indel |  | 100 (85.75, 100) | 0.93% VAF | 0.38% VAF |
| BRCA1 P871fs*32 | Indel |  | 100 (85.18, 100) | 0.51% VAF | 0.38% VAF |

| **Targeted Variant** | **Variant Subtype** | **Cancer Type** | **Reproducibility**  **(%) (95% Two- sided CI)** | **Observed**  **Average**  **Measurand** | **LoD** |
| --- | --- | --- | --- | --- | --- |
| BRCA1 P871fs*32 | Indel |  | 100 (85.75, 100) | 1.08% VAF | 0.38% VAF |
| BRCA1 Y465* | Sub | Prostate cancer | 100 (86.2, 100) | 0.51% VAF^1^ | 0.34% VAF |
| BRCA1_D1840fs*32 | del |  | 95.83 (79.76,  99.26) | 0.55% VAF^1^ | 0.38% VAF |
| BRCA1_N/A_truncation | RE |  | 100 (86.2, 100) | 1.27% VAF^1^ | Not Determined |
| BRCA2 C1200fs*1 | Indel | Contrived | 100 (85.75, 100) | 0.58% VAF | 0.36% VAF |
| BRCA2 C1200fs*1 | Indel |  | 100 (85.75, 100) | 0.92% VAF | 0.36% VAF |
| BRCA2 N1784fs*7 | Indel |  | 100 (85.75, 100) | 1.22% VAF | 0.36% VAF |
| BRCA2 N1784fs*7 | Indel |  | 100 (85.75, 100) | 1.85% VAF | 0.36% VAF |
| BRCA2 N1784fs*7 | Indel |  | 100 (85.18, 100) | 1.07% VAF | 0.36% VAF |
| BRCA2 N1784fs*7 | Indel |  | 100 (85.75, 100) | 2.24% VAF | 0.36% VAF |
| BRCA2 N1822fs*2 | Indel |  | 100 (85.75, 100) | 0.92% VAF | 0.36% VAF |
| BRCA2 N1822fs*2 | Indel |  | 100 (85.18, 100) | 1.19% VAF | 0.36% VAF |
| BRCA2 Q1429fs*9 | Indel |  | 100 (85.75, 100) | 0.94% VAF | 0.36% VAF |
| BRCA2 Q1429fs*9 | Indel |  | 100 (85.18, 100) | 1.26% VAF | 0.36% VAF |
| BRCA2 T3033fs*11 | Indel |  | 21.74 (7.46, 43.7) | 0.71% VAF | 0.36% VAF |
| BRCA2 T3033fs*11 | Indel |  | 91.67 (73, 98.97) | 1.03% VAF | 0.36% VAF |
| BRCA2_CDH17_truncation | RE | Prostate cancer | 100 (86.2, 100) | 1.49% VAF^1^ | Not Determined |
| BRCA2_G995fs*4 | del |  | 95.83 (79.76,  99.26) | 0.63% VAF^1^ | 0.36% VAF |
| BRCA2_loss | CN |  | 100 (86.2, 100) | 53.11% TF^1^ | 48.1% TF |
| BRCA2_loss | CN |  | 87.5 (67.64, 97.34) | 39.43% TF | 48.1% TF |
| BRCA2_N/A_truncation | RE |  | 70.83 (50.83,  85.09) | 1.32% VAF | 0.48% VAF |
| BRCA2_Q1361* | sub |  | 100 (85.69, 100) | 0.71% VAF^1^ | 0.49% VAF |
| BRCA2-EDA truncation | RE |  | 100 (85.18, 100) | 0.48% VAF^1^ | 0.47% VAF^2^ |
| EGFR E746 A750del | Indel | Lung cancer | 95.7 (79, 99.2) | 0.45% VAF^1^ | 0.27% VAF |
| EGFR E746_A750del | Indel |  | 100 (84.56, 100) | 0.34% VAF^1^ | 0.27% VAF |
| EGFR E746_A750del | Indel | Contrived | 100 (85.75, 100) | 0.51% VAF | 0.27% VAF |
| EGFR E746_A750del | Indel |  | 100 (85.75, 100) | 0.74% VAF | 0.27% VAF |
| EGFR E746_A750del | Indel |  | 100 (85.75, 100) | 0.93% VAF | 0.27% VAF |
| EGFR E746_A750del | Indel |  | 100 (85.18, 100) | 1.2% VAF | 0.27% VAF |
| EGFR E746_A750del | Indel |  | 100 (85.18, 100) | 0.51% VAF | 0.27% VAF |
| EGFR E746_A750del | Indel |  | 100 (85.75, 100) | 1.01% VAF | 0.27% VAF |
| EGFR L858R | Sub | Lung cancer | 100 (85.75, 100) | 0.64% VAF^1^ | 0.34% VAF |
| EGFR L858R | Sub |  | 100 (85.75, 100) | 1.64% VAF^1^ | 0.34% VAF |
| EGFR L858R | Sub | Contrived | 100 (85.75, 100) | 0.46% VAF | 0.34% VAF |
| EGFR L858R | Sub |  | 100 (85.75, 100) | 0.68% VAF | 0.34% VAF |
| EGFR L858R | Sub |  | 100 (85.75, 100) | 0.68% VAF | 0.34% VAF |
| EGFR L858R | Sub |  | 100 (85.18, 100) | 0.95% VAF | 0.34% VAF |
| ETV6-NTRK3 fusion | RE | Thyroid cancer | 100 (86.20, 100) | 0.82% VAF^1^ | 0.27% VAF |
| ETV6-NTRK3 fusion | RE | Contrived | 95.83 (78.88,  99.89) | 0.32% VAF | 0.474% VAF^2^ |
| ETV6-NTRK3 fusion | RE |  | 95.83 (78.88,  99.89) | 0.59% VAF | 0.474% VAF^2^ |
| ETV6-NTRK3 fusion | RE | Lung cancer | 100 (85.75, 100) | 26.33% VAF | 0.474% VAF^2^ |
| ETV6-NTRK3 fusion | RE | Salivary gland cancer | 100 (85.69, 100) | 0.68% VAF^1^ | 0.27% VAF |
| GOPC-ROS1 fusion | RE | Contrived | 86.96 (66.41,  97.22) | 0.35% VAF | 0.474% VAF^2^ |
| **Targeted Variant** | **Variant Subtype** | **Cancer Type** | **Reproducibility**  **(%) (95% Two- sided CI)** | **Observed**  **Average**  **Measurand** | **LoD** |
| GOPC-ROS1 fusion | RE |  | 91.67 (73, 98.97) | 0.91% VAF | 0.474% VAF^2^ |
| MET exon14 splice site 288835_2889>A | Indel | Lung cancer | 95.8 (79.8, 99.3) | 0.28% VAF^1^ | 0.41% VAF |
| MET exon14 splice site 3028+1G>T | Sub |  | 95.8 (79.8, 99.3) | 0.45% VAF^1^ | Not Determined |
| MET exon14 splice site 3028+2T>C | Sub |  | 95.7 (79.0, 99.2) | 0.35% VAF^1^ | Not Determined |
| MET exon14splice site 3028+1G>T | Sub |  | 100 (85.7, 100) | 0.85% VAF | Not Determined |
| MET exon14splice site 3028+2T>C | Sub |  | 100 (85.75, 100) | 0.76% VAF | Not Determined |
| MET splice site 3029-1G>T | Sub | Contrived | 62.5 (40.59, 81.2) | 0.21% VAF | Not Determined |
| MET splice site 3029-1G>T | Sub |  | 91.3 (71.96, 98.93) | 0.3% VAF | Not Determined |
| MET splice site 2888- 17_2888-3del15 | Indel | Lung cancer | 100 (85.75, 100) | 1.17% VAF^1^ | 0.41% VAF |
| MET splice site  3005_3028+3>C | Indel |  | 100 (85.75, 100) | 1.67% VAF^1^ | 0.41% VAF |
| MPRIP-NTRK1 fusion | RE | Contrived | 69.57 (47.08,  86.79) | 0.49% VAF | 0.44% VAF |
| MPRIP-NTRK1 fusion | RE |  | 87.5 (67.64, 97.34) | 0.69% VAF | 0.44% VAF |
| PIK3CA E542K | Sub | Breast cancer | 100 (85.75, 100) | 0.89% VAF^1^ | 0.34% VAF |
| PIK3CA E545A | Sub | Contrived | 100 (85.75, 100) | 0.52% VAF | 0.34% VAF |
| PIK3CA E545A | Sub |  | 100 (85.18, 100) | 0.7% VAF | 0.34% VAF |
| PIK3CA E545K | Sub |  | 100 (85.75, 100) | 0.45% VAF | 0.34% VAF |
| PIK3CA E545K | Sub |  | 100 (85.75, 100) | 0.66% VAF | 0.34% VAF |
| PIK3CA H1047R | Sub | Breast cancer | 100 (85.75, 100) | 1.04% VAF^1^ | 0.34% VAF |
| PIK3CA H1047R | Sub | Contrived | 100 (85.18, 100) | 0.41% VAF | 0.34% VAF |
| PIK3CA H1047R | Sub |  | 100 (85.75, 100) | 0.76% VAF | 0.34% VAF |
| PIK3CA Q546R | Sub | Breast cancer | 91.7 (74.2, 97.7) | 0.44% VAF | 0.34% VAF |
| PIK3CA Q546R | Sub | Contrived | 95.65 (78.05,  99.89) | 0.49% VAF | 0.34% VAF |
| PIK3CA Q546R | Sub |  | 100 (85.75, 100) | 0.92% VAF | 0.34% VAF |
| PIK3CA_H1047R | Sub | Breast cancer | 95.65 (79.01,  99.23) | 0.39% VAF^1^ | 0.34% VAF |
| ROS1-CD74 fusion | RE | Lung cancer | 100 (85.75, 100) | 1.32% VAF^1^ | 0.52% VAF |
| ROS1-EZR fusion | RE |  | 100 (85.75, 100) | 1.3% VAF^1^ | 0.52% VAF |
| SLC34A2-ROS1 fusion | RE | Contrived | 100 (85.75, 100) | 1.03% VAF | 0.284% VAF^2^ |
| SLC34A2-ROS1 fusion | RE |  | 100 (85.18, 100) | 1.36% VAF | 0.284% VAF^2^ |
| TPM3-NTRK1 fusion | RE | Lung cancer | 91.67 (73, 98.97) | 8.48% VAF | 0.44% VAF |
| TPM3-NTRK1 fusion | RE | Contrived | 100 (85.75, 100) | 0.3% VAF | 0.44% VAF |
| TPM3-NTRK1 fusion | RE |  | 100 (85.75, 100) | 0.4% VAF | 0.44% VAF |
| TPM3-NTRK1 fusion | RE | Colon cancer | 100 (85.69, 100) | 0.83% VAF^1^ | 0.44% VAF |
| TPR-NTRK1 fusion | RE | Thyroid cancer | 100 (85.69, 100) | 0.75% VAF^1^ | 0.44% VAF^2^ |

^1^LoD was confirmed for these variants with hit rate (same as the reproducibility) which met the acceptance criteria defined in respective study.

^2^ LoD was not determined for these specific variants; platform LoD for the variant type is listed.

Table 23. Precision and Confirmation of LoD by Targeted Non-CDx Variant

| **Targeted Variant** | **Variant Subtype** | **Cancer Type** | **Reproducibility (%) (95% Two-sided CI)** | **Observed**  **Average**  **Measurand** | **LoD** |
| --- | --- | --- | --- | --- | --- |
| BRAF L597R | Sub | Contrived | 95.65 (78.05, 99.89) | 0.42% VAF | 0.49% VAF |
| BRAF L597R | Sub |  | 100 (85.75, 100) | 0.85% VAF | 0.49% VAF |
| BRAF V600E | Sub | Skin cancer | 100 (85.75, 100) | 0.44% VAF^1^ | 0.33% VAF |
| **Targeted Variant** | **Variant Subtype** | **Cancer Type** | **Reproducibility (%) (95% Two-sided CI)** | **Observed**  **Average**  **Measurand** | **LoD** |
| BRAF V600E | Sub | Contrived | 100 (85.18, 100) | 0.72% VAF | 0.49% VAF |
| BRAF V600E | Sub |  | 100 (85.75, 100) | 1.38% VAF | 0.49% VAF |
| BRAF V600K | Indel | Skin cancer | 95.83 (78.88, 99.89) | 0.36% VAF^1^ | 0.33% VAF |
| BRCA1 E23fs*17 | Indel | Ovarian cancer | 100 (85.75, 100) | 0.66% VAF^1^ | 0.38% VAF |
| BRCA1 Q780* | Sub |  | 100 (85.75, 100) | 1.11% VAF^1^ | 0.34% VAF |
| BRCA1_S646fs*5 | del |  | 100 (85.69, 100) | 0.54% VAF^1^ | 0.38% VAF |
| BRCA1_Y1563* | Sub |  | 100 (86.2, 100) | 1.66% VAF^1^ | 0.51% VAF |
| BRCA1-BRCA1 deletion | RE |  | 100 (85.75, 100) | 0.87% VAF^1^ | 0.28% VAF^2^ |
| BRCA2 G267* | Sub |  | 91.67 (73, 98.97) | 0.5% VAF | Not Determined |
| BRCA2 S2988fs*12 | Indel |  | 100 (85.75, 100) | 1.07% VAF^1^ | 0.36% VAF |
| BRCA2_E2198fs*4 | del |  | 100 (86.2, 100) | 0.65% VAF^1^ | 0.36% VAF |
| BRCA2_N3124I | Sub |  | 100 (86.2, 100) | 0.74% VAF^1^ | 0.49% VAF |
| BRCA2 R2842C | Sub | Lung cancer | 100 (85.7, 100) | 0.57% VAF^1^ | 0.49% VAF |
| EGFR S492R | Sub | Colon cancer | 71.4 (45.4, 88.3) | 0.39% VAF | 0.34% VAF |
| EGFR T790M | Sub | Lung cancer | 100 (85.75, 100) | 1.26% VAF^1^ | 0.34% VAF |
| EGFR T790M | Sub | Contrived | 100 (85.18, 100) | 0.36% VAF | 0.49% VAF |
| EGFR T790M | Sub |  | 100 (85.75, 100) | 0.65% VAF | 0.49% VAF |
| EGFR T790M | Sub |  | 100 (85.75, 100) | 0.44% VAF | 0.49% VAF |
| EGFR T790M | Sub |  | 100 (85.75, 100) | 0.66% VAF | 0.49% VAF |
| EGFR ex20 insertion H773_V774insH | Indel | Lung Cancer | 100 (86.2, 100) | 0.98% VAF^1^ | Not Determined |
| EGFR ex20 insertion V769_D770insASV | Indel |  | 100 (86.2, 100) | 1.28% VAF^1^ | Not Determined |
| EGFR ex20 insertion D770_N771insSVD | Indel |  | 100 (86.2, 100) | 0.65% VAF^1^ | Not Determined |
| ERBB2_amplification | CN | Breast cancer | 100 (85.75, 100) | 61.73% TF^1^ | 19.8% TF |
| ERBB2_amplification | CN | Lung cancer | 100 (85.69, 100) | 0% TF^1^ | 19.8% TF |
| ERBB2_amplification | CN | Colon cancer | 100 (86.2, 100) | 31.05% TF^1^ | 19.8% TF |
| ERBB2_amplification | CN | Unknown primary cancer | 100 (85.69, 100) | 33.12% TF^1^ | 19.8% TF |
| ERBB2_amplification | CN | Contrived | 100 (85.75, 100) | 35.78% TF | 25.2% TF |
| ERBB2_amplification | CN |  | 100 (85.75, 100) | 39.79% TF | 25.2% TF |
| ERBB2_amplification | CN | Soft tissue cancer | 0 (0, 13.8) | 54.53% TF | 19.8% TF |
| ERBB2_amplification | CN | Lung cancer | 0 (0, 14.31) | 54.8% TF | 19.8% TF |
| KRAS G12D | Sub | Contrived | 100 (85.75, 100) | 0.89% VAF | 0.49% VAF |
| KRAS G12D | Sub |  | 100 (85.18, 100) | 1.12% VAF | 0.49% VAF |
| KRAS G12L | Sub | Colon cancer | 100 (85.75, 100) | 0.49% VAF^1^ | 0.33% VAF |
| KRAS G13D | Sub | Contrived | 100 (85.75, 100) | 0.55% VAF | 0.49% VAF |
| KRAS G13D | Sub |  | 100 (85.75, 100) | 0.82% VAF | 0.49% VAF |
| KRAS G13D | Sub |  | 100 (85.18, 100) | 0.57% VAF | 0.49% VAF |
| KRAS G13D | Sub |  | 100 (85.75, 100) | 0.92% VAF | 0.49% VAF |
| KRAS Q61R | Sub | Colon cancer | 100 (85.75, 100) | 0.53% VAF^1^ | 0.33% VAF |
| MET L1312fs*4 | Indel | Contrived | 100 (85.75, 100) | 0.69% VAF | 0.56% VAF |
| MET L1312fs*4 | Indel |  | 100 (85.75, 100) | 0.96% VAF | 0.56% VAF |
| NRAS G12C | Sub |  | 100 (85.75, 100) | 0.69% VAF | 0.49% VAF |
| NRAS G12C | Sub |  | 100 (85.75, 100) | 0.96% VAF | 0.49% VAF |
| NRAS G12C | Sub | Lung cancer | 91.3 (73.2, 97.6) | 0.55% VAF | 0.42% VAF |
| NRAS G12D | Sub | Contrived | 82.61 (61.22, 95.05) | 0.48% VAF | 0.49% VAF |

| **Targeted Variant** | **Variant Subtype** | **Cancer Type** | **Reproducibility (%) (95% Two-sided CI)** | **Observed**  **Average**  **Measurand** | **LoD** |
| --- | --- | --- | --- | --- | --- |
| NRAS G12D | Sub |  | 100 (85.75, 100) | 0.84% VAF | 0.49% VAF |
| NTRK2-N/A rearrangement | RE |  | 95.83 (78.88, 99.89) | 1.85% VAF | 0.897% VAF |
| NTRK2-N/A rearrangement | RE |  | 95.83 (78.88, 99.89) | 2.03% VAF | 0.897% VAF |
| PALB2 G808* | Sub |  | 100 (85.18, 100) | 0.47% VAF | 0.49% VAF |
| PALB2 G808* | Sub |  | 100 (85.75, 100) | 0.92% VAF | 0.49% VAF |
| PALB2 K908fs*15 | Indel |  | 100 (85.75, 100) | 0.52% VAF | 0.56% VAF |
| PALB2 K908fs*15 | Indel |  | 100 (85.75, 100) | 0.74% VAF | 0.56% VAF |
| PALB2 N280fs*8 | Indel | Colon cancer | 100 (56.6, 100) | 0.48% VAF^1^ | 0.37% VAF |
| PIK3CA D549N | Sub | Contrived | 100 (85.75, 100) | 0.48% VAF | 0.49% VAF |
| PIK3CA D549N | Sub |  | 100 (85.75, 100) | 0.73% VAF | 0.49% VAF |

1. LoD was confirmed for these variants with hit rate (same as the reproducibility) which met the acceptance criteria defined in respective study.
2. LoD was not determined for these specific variants; platform LoD for the variant type is listed.

Assessment of Tumor Profiling Variants

Across 39 unique samples, including 8 contrived samples, and 31 clinical samples, a total of 1,240 variants were evaluated for reproducibility and repeatability of tumor profiling variants, with variant types including substitutions, indels, rearrangements, and copy number alterations. The number of variants in each variant bin are summarized in **Table 24.** The overall reproducibility results were 99.59% with the 95% 2-sided exact CIs [99.58%, 99.60%]. The overall repeatability for all variants were 99.47% with 95% 2-sided exact CIs [99.45%, 99.48%]. The reproducibility and repeatability results for each variant type are summarized in **Table 24**.

Table 24. Number of each variant type

| **Variant Category** | **N** | **# of Pairs Agree/ # of Total Pairs** | **Repeatability (%)**  **[95% Two-Sided Exact CIs (%)]** | **# of Replicates**  **Agree/**  **# of Total**  **Replicates** | **Reproducibility**  **(%)**  **[95% Two-Sided Exact CIs (%)]** |
| --- | --- | --- | --- | --- | --- |
| **Substitutions** | **898** |  |  |  |  |
| Substitution in a non-repetitive region or a repetitive region of <=7 base pairs | 882 |  |  |  |  |
| Substitution in a repetitive region of >7 base pairs | 16 |  |  |  |  |
| **Indels** | **228** | 126475 / 127224 | 99.41  [99.37, 99.45] | 254509 / 255588 | 99.58  [99.55, 99.60] |
| Insertion/Deletion in non-repetitive region or a repetitive region of <=3 base pairs | 52 |  |  |  |  |
| Insertion/Deletion in a repetitive region of 4 to 6 base pairs | 118 |  |  |  |  |
| Insertion/Deletion in a repetitive region of >=7 base pairs | 58 |  |  |  |  |
| **Rearrangements** | **60** | 33105 / 33480 | 98.88  [98.76, 98.99] | 66723 / 67260 | 99.20  [99.13, 99.27] |
| **Copy Number Alterations** | **54** | 29880 / 30132 | 99.16  [99.05, 99.26] | 60115 / 60534 | 99.31  [99.24, 99.7] |
| Copy Number Amplification | 49 |  |  |  |  |
| Copy Number Loss | 5 |  |  |  |  |
| **Total** | **1240** | **688225 / 691920** | **99.47**  **[99.45, 99.48]** | **1384328 / 1390040** | **99.59**  **[99.58, 99.60]** |

##### 9.14 Reagent Lot Interchangeability

The interchangeability of critical reagent lots for library construction (LC), hybrid capture (HC) and sequencing within the FoundationOne Liquid CDx assay was evaluated by testing eight (8) contrived samples from either enzymatically fragmented cell line genomic DNA containing alterations of interest or enzymatically fragmented plasmid DNA. Each of the contrived samples was tested in triplicate using two different lots each of LC, HC, and sequencing reagents. Eight reagent pairings were assessed. A total of eight analyses for each specimen were completed. A total of 192 tests were included in this study. Four Master Pool Libraries (MPLs) were evaluated on each of two flowcells on a NovaSeq 6000 sequencer, using two different sequencing reagent lots. Of the 49 alterations assessed in the sample set, 43 had a percent agreement greater than 90% (39 alterations had percentage agreement equal to 100%, one had percent agreement equal to 95.83%, one had percent agreement equal to 95.65%, and two had percent agreement equal to 91.67%), exceeding the pre-specified acceptance criteria. For the remaining six alterations the observed detection rates for these variants were similar to the predicted detection rate based on the LoD analysis. These results demonstrate the interchangeability of critical reagent lots in the FoundationOne Liquid CDx assay.

##### 9.15 Variant Curator Precision

This study was performed to evaluate the precision of genomic variant call curation, following analysis by the FoundationOne Liquid CDx analysis pipeline. This was established by analyzing targeted alterations, including CDx alterations, and platform-wide alterations within samples used in the FoundationOne Liquid CDx Precision and LoD and Precision Confirmation Study. The study design reflected the intermediate precision design and evaluated curator precision in reporting of targeted and platform alterations. A total of 19 samples were selected for this study. Three curators were chosen randomly amongst all qualified curators to curate variant calls in a set of randomly chosen replicates from each of the 19 samples. The variant calls were generated from each sample per curator. The overall average percent agreement for targeted alterations was 93.3% (95% CI; 83.80%, 98.15%), and for platform genomic alterations was 99.14% (95% CI; 98.47%, 99.57%).

##### 9.16 Stability

**9.16.1 Reagent Stability**

The reagent stability of FoundationOne Liquid CDx was assessed by analyzing data from each of eight samples in triplicate, per each of three different lots of LC, HC, and sequencing reagents. A total of nine analyses for each specimen were completed for each of six time points assessed. A total of 72 tests were assessed per time period; a total of 432 samples and six time points (one baseline timepoint and 5 subsequent experimental timepoints) were included in this study overall. Each of the three sample Master Library Pools (MPLs), representing three LC and HC reagent lots was evaluated per time point on a NovaSeq 6000 sequencer, using three different sequencing reagent lots. The analysis of baseline timepoint zero (T0) identified the baseline variant calls for each sample.

All five experimental time points have been processed and analyzed for Lot #1, Lot #2, and Lot #3. Concordance was assessed among 127,642 data points for tumor profiling variants across the five experimental timepoints. The three reagent lots achieved ≥90% concordance with the baseline variant calls for all the experimental timepoints (including the last two timepoints T4 and T5 at 12 and 13 months respectively) except for a middle timepoint T3 (9 months) which is present in **Table 25**. The reason for the failure of T3 (9 months) was a technical error which resulted in lower than planned DNA being transferred for LC and therefore this was not a reagent failure. Reagent stability can be claimed as 12 months.

**Table 25. Concordance for Tumor Profiling Variants at Replicate Level by Reagent Lot and by Timepoint**

| **Reagent Lot** | **Timepoint^1^** | **# Concordant** | **# Total** | **Concordance (%)** | **95% 2-sided score CI (%)** |
| --- | --- | --- | --- | --- | --- |
| LOT#1 | 3 months | 1921 | 1966 | 97.71% | [96.95%, 98.28%] |
|  | 6 months | 2082 | 2151 | 96.79% | [95.96%, 97.46%] |
|  | 9 months | 1916 | 2151 | 89.07% | [87.69%, 90.32%] |
|  | 12 months | 1609 | 1656 | 97.16% | [96.25%, 97.86%] |
|  | 13 months | 1918 | 1973 | 97.21% | [96.39%, 97.85%] |
| LOT#2 | 3 months | 2083 | 2148 | 96.97% | [96.16%, 97.62%] |
|  | 6 months | 2091 | 2160 | 96.81% | [95.98%, 97.47%] |
|  | 9 months | 1851 | 2160 | 85.69% | [84.15%, 87.11%] |
|  | 12 months | 2087 | 2160 | 96.62% | [95.77%, 97.3%] |
|  | 13 months | 2089 | 2160 | 96.71% | [95.87%, 97.39%] |
| LOT#3 | 3 months | 2086 | 2139 | 97.52% | [96.77%, 98.10%] |
|  | 6 months | 2098 | 2154 | 97.4% | [96.64%, 97.99%] |
|  | 9 months | 1855 | 2154 | 86.12% | [84.59%, 87.51%] |
|  | 12 months | 2097 | 2154 | 97.35% | [96.59%, 97.95%] |
|  | 13 months | 1924 | 1977 | 97.32% | [96.51%, 97.94%] |

A supplemental study is being conducted to evaluate the stability of updated LC reagents. The study will confirm that reagent stability can be claimed as 12 months for the FoundationOne Liquid CDx assay with the changed reagents.

**9.16.2 Whole Blood Specimen Stability**

The recommended storage temperature is 18°C - 25°C. In this study, stress conditions were simulated through extended storage at elevated (35°C ± 2°C) and reduced (4° ± 2°C) temperatures.

In this interim analysis, 22 samples (11 sample pairs) were tested, including baseline (within 24 hours of collection) and experimental time points (after 10, 14, or 15 days of storage).

Overall, 100% of samples yielded a cfDNA input ≥30ng. The success rate for DNAx yield, and LC yield was 100% and the success rate of the HC yield was 96.3%. The variant analysis was conducted for variants at ≥2x LoD. For the aggregate 11 pairs of samples processed and reported, 100% agreement was observed between the baseline and experimental timepoint for short variants and rearrangements for each experimental time point. The percent agreement per sample also resulted in 100% agreement between the baseline and experimental timepoint for short variants and rearrangements. The data is summarized in **Table 26.**

**Table 26. Aggregate percent agreement per temperature and experimental timepoint**

| **Temperature** | **Experimental Timepoint** | **N** | **Short Variants [95% two-sided CI]** | **Rearrangements** |
| --- | --- | --- | --- | --- |
| 4°C | 7 Days | 4 | 100.00 [89.72, 100.00] | 100.00 [39.76, 100.00] |
|  | 14 Days | 3 | 100.00 [91.40, 100.00] | N/A |
|  | 15 Days | 3 | 100.00 [83.89, 100.00] | N/A |
| 35°C | 14 Days | 1 | N/A | N/A |

The impact of potential interferents originating from the FoundationOne Liquid cfDNA blood collection tube (BCT) stopper on the performance of the FoundationOne Liquid CDx assay was assessed by evaluating stability of whole blood in tubes stored in an upright or inverted position at 4°C± 2°C, 25°C± 2°C, and 35°C± 2°C for various durations (10, 14, and 15 days).

First, the success rate of the FoundationOne Liquid CDx assay for processing samples was assessed at the DNA extraction (DNAx), LC, HC, and sequencing steps based on product in-process QC criteria. Samples stratified by the upright and the inverted condition exhibited comparable success rates above 94% at DNAx, LC, HC, and sequencing (**Table 27**). Thus, the stopper of the FoundationOne Liquid cfDNA BCT does not impact FoundationOne Liquid CDx test performance when stored between 4°C - 35°C for up to 15 days.

**Table 27. Process success rate by tube position**

| **Process** | **Tube Position** | **# Passing Samples** | **# Total Samples** | **Success Rate (%)** | **95% 2-sided CIs (%)** |
| --- | --- | --- | --- | --- | --- |
| DNA  Extraction | Upright | 139 | 147 | 94.6% | [89.6%, 97.2%] |
|  | Inverted | 147 | 150 | 98% | [94.3%, 99.3%] |
| LC | Upright | 135 | 136 | 99.3% | [96%, 99.9%] |
|  | Inverted | 146 | 146 | 100% | [97.4%, 100%] |
| HC | Upright | 134 | 135 | 99.3% | [95.9%, 99.9%] |
|  | Inverted | 143 | 146 | 97.9% | [94.1%, 99.3%] |
| Sequencing | Upright | 134 | 134 | 100% | [97.2%, 100%] |
|  | Inverted | 143 | 143 | 100% | [97.4%, 100%] |

Stability was also evaluated by comparing concordance between baseline and experimental samples. PPA and NPA for alteration calls at ≥ 2x LoD were computed along with the corresponding two-sided 95% score confidence interval (CI) across all replicates by variant category using the baseline detection as reference. Note that NPA is under-estimated as variants not detected at any of the treatment conditions were not used in the analysis set and hence counted against the NPA calculation.

Concordance between baseline and experimental results from all samples in the upright and inverted position combined demonstrated >99% PPA and NPA for the detection of short variants and rearrangements. Copy number alterations were only detected in samples treated in the inverted tube position and therefore, not included in this analysis. Furthermore, stratification by the treatment condition (2 tube positions × 3 temperatures × 3 durations) revealed >99.0% PPA and NPA for short variants and rearrangements across the combinations of tube positions, temperatures and durations tested. The data also demonstrate that the detection of copy number alterations is not impacted by the storage of blood in the inverted position at 35°C for up to 14 days. The concordance results by variant type for each of the experimental conditions are provided in **Table 28**.

**Table 28. Concordance of detected alterations between baseline sample and experimental conditions for inverted tube stability study**

| **Variant Type** | **Temp.** | **Tube Position** | **Exp. Time Point** | **N**  **Variants**  **Detected at Baseline**  **Time Point** | **N**  **Variants**  **Detected**  **at Exp.**  **Time Point** | **N**  **Variants Agree** | **PPA** | **PPA [95%**  **CI]** | **N**  **Variants Not**  **Detected**  **at**  **Baseline**  **Time**  **Point** | **N**  **Variants Not**  **Detected at Exp.**  **Time Point** | **NPA** | **NPA [95%**  **CI]** |
| --- | --- | --- | --- | --- | --- | --- | --- | --- | --- | --- | --- | --- |
| **Short variants** | 04°C | Inverted | Day 10 | 50 | 50 | 49 | 98% | [89.5%,  99.6%] | 612 | 612 | 100% | [100%, 100%] |
| **Short variants** | 04°C | Upright | Day 10 | 50 | 51 | 50 | 100% | [92.9%, 100%] | 613 | 612 | 100% | [100%, 100%] |
| **Short variants** | 04°C | Inverted | Day 14 | 59 | 58 | 58 | 98.3% | [90.9%,  99.7%] | 610 | 611 | 100% | [100%, 100%] |
| **Short variants** | 04°C | Upright | Day 14 | 44 | 44 | 44 | 100% | [92.0%, 100%] | 611 | 611 | 100% | [100%, 100%] |
| **Variant Type** | **Temp.** | **Tube Position** | **Exp. Time Point** | **N**  **Variants**  **Detected at Baseline**  **Time Point** | **N**  **Variants**  **Detected**    **at Exp.**  **Time Point** | **N**  **Variants Agree** | **PPA** | **PPA [95%**  **CI]** | **N**  **Variants Not**  **Detected**  **at**  **Baseline**  **Time**  **Point** | **N**  **Variants Not**  **Detected at Exp.**  **Time Point** | **NPA** | **NPA [95%**  **CI]** |
| **Short variants** | 04°C | Inverted | Day 15 | 37 | 37 | 37 | 100% | [90.6%, 100%] | 611 | 611 | 100% | [100%, 100%] |
| **Short variants** | 04°C | Upright | Day 15 | 52 | 52 | 52 | 100% | [93%,  100%] | 611 | 611 | 100% | [100%, 100%] |
| **Short variants** | 25°C | Inverted | Day 10 | 78 | 77 | 76 | 97.1% | [91.1%,  99.2%] | 627 | 628 | 100% | [100%, 100%] |
| **Short variants** | 25°C | Upright | Day 10 | 44 | 44 | 44 | 100% | [92.0%, 100%] | 613 | 613 | 100% | [100%, 100%] |
| **Short variants** | 25°C | Inverted | Day 14 | 46 | 48 | 46 | 100% | [92.3%, 100%] | 611 | 609 | 100% | [100%, 100%] |
| **Short variants** | 25°C | Upright | Day 14 | 42 | 41 | 41 | 97.6% | [87.7%,  99.6%] | 610 | 611 | 100% | [100%, 100%] |
| **Short variants** | 25°C | Inverted | Day 15 | 44 | 44 | 44 | 100% | [92.0%, 100%] | 613 | 613 | 100% | [100%, 100%] |
| **Short variants** | 25°C | Upright | Day 15 | 49 | 48 | 48 | 97.8% | [89.3%,  99.6%] | 616 | 617 | 100% | [100%, 100%] |
| **Short variants** | 35°C | Inverted | Day 10 | 15 | 15 | 15 | 100% | [79.6%, 100%] | 609 | 609 | 100% | [100%, 100%] |
| **Short variants** | 35°C | Upright | Day 10 | 35 | 35 | 35 | 100% | [90.1%, 100%] | 609 | 609 | 100% | [100%, 100%] |
| **Short variants** | 35°C | Inverted | Day 14 | 55 | 55 | 55 | 100% | [93.4%, 100%] | 611 | 611 | 100% | [100%, 100%] |
| **Short variants** | 35°C | Upright | Day 14 | 48 | 47 | 46 | 95.7% | [86.0%,  98.8%] | 609 | 610 | 100% | [100%, 100%] |
| **Short variants** | 35°C | Inverted | Day 15 | 39 | 39 | 38 | 97.4% | [86.8%,  99.5%] | 610 | 610 | 100% | [100%, 100%] |
| **Short variants** | 35°C | Upright | Day 15 | 28 | 29 | 28 | 100% | [87.9%, 100%] | 613 | 612 | 100% | [100%, 100%] |

These results demonstrate that blood is stable in the FoundationOne Liquid CDx cfDNA BCT when stored between 4°C - 35°C for up to 15 days, in an upright or inverted position. Additional data will be generated to further evaluate whole blood stability and potential interference of the blood collection tube cap.

##### 9.17 DNA Extraction

DNA extraction evaluated 72 samples across five cancer types: lung cancer (including NSCLC), CRC, prostate cancer, breast cancer, and skin cancer (melanoma, sarcoma) using three reagent lots and two KingFisher Magnetic Particle processors.

Reproducibility of the FoundationOne Liquid CDx DNA extraction process across KingFisher instruments and extraction reagent lots were analyzed utilizing a factorial design (3 reagent lots × 2 KingFisher instruments × 2 replicates). The success rate of the DNAx yield for three reagent lots range from 95.8% to 100.0% and two King Fisher instruments range from 97.2% to 100.0%.

Variant calls included in the concordance analysis were identified based on the majority call across all 12 replicates for a given disease ontology. PPA and NPA were computed across the replicates for each somatic alteration for each sample, and aggregated by variant type (deletion, insertion, rearrangement, and substitution) for variants at ≥1x LoD. The percent agreement results by disease ontologies are: 90.3% - 99.8 % for PPA, and 99.1% - 100.0% for NPA (**Table 29**) The percent agreement results across all variant types (deletion, insertion, rearrangement and substitution) evaluated at ≥1x LoD are: 90.6%- 96.8% for PPA and 98.9% - 100.0% for NPA (**Table 30**).

**Table 29. Concordance summary by disease ontology at 1x LoD for DNA extraction study**

| **Disease Ontology** | **Positive**  **Detecte d/**  **Positive Total** | **PPA**  **[95% two-sided CI]** | **Negative**  **Detected/**  **Negative**  **Total**^1^ | **NPA**  **[95% two-sided**  **CI]** | **Overall**  **Detected/ Total*** | **OPA**  **[95% two-sided CI]** |
| --- | --- | --- | --- | --- | --- | --- |
| Breast Cancer | 347/348 | 99.7%  [98.4%,100.0%] | 3144/3144 | 100.0%  [99.9%,100.0%] | 3491/3492 | 100.0%  [99.8%,100.0%] |
| CRC | 1122/1188 | 94.4%  [93.0%,95.7%] | 2284/2304 | 99.1%  [98.7%,99.5%] | 3406/3492 | 97.5%  [97.0%,98.0%] |
| Lung Cancer | 431/432 | 99.8%  [98.7%,100.0%] | 3053/3060 | 99.8%  [99.5%,99.9%] | 3484/3492 | 99.8%  [99.5%,99.9%] |
| NSCLC | 600/612 | 98.0%  [96.6%,99.0%] | 2878/2880 | 99.9%  [99.7%,100.0%] | 3478/3492 | 99.6%  [99.3%,99.8%] |
| Prostate Cancer | 486/492 | 98.8%  [97.4%,99.6%] | 2987/3000 | 99.6%  [99.3%,99.8%] | 3473/3492 | 99.5%  [99.2%,99.7%] |
| Skin Cancer | 455/504 | 90.3%  [87.4%,92.7%] | 2987/2988 | 100.0%  [99.8%,100.0%] | 3442/3492 | 98.6%  [98.1%,98.9%] |

^1^Variants detected include variants classified as VUS and benign

**Table 30. Concordance summary by variant type at 1x LoD for DNA extraction study**

| **Variant Type** | **Positive**  **Detected/**  **Positive Total** | **PPA**  **[95% two-sided CI]** | **Negative**  **Detected/**  **Negative Total**^1^ | **NPA**  **[95% two- sided CI]** | **Overall**  **Detected/ Total*** | **OPA**  **[95% two- sided CI]** |
| --- | --- | --- | --- | --- | --- | --- |
| **Deletions** | 386/ 408 | 94.6%  [91.9%, 96.6%] | 2036/ 2040 | 99.8%  [99.5%, 99.9%] | 2422/ 2448 | 98.9%  [98.4%, 99.3%] |
| **Insertions** | 163/ 180 | 90.6%  [85.3%, 94.4%] | 819/ 828 | 98.9%  [97.9%, 99.5%] | 982/ 1008 | 97.4%  [96.2%, 98.3%] |
| **Rearrangements** | 23/ 24 | 95.8%  [78.9%, 99.9%] | 120/ 120 | 100.0%  [97.0%, 100.0%] | 143/ 144 | 99.3%  [96.2%, 100.0%] |
| **Substitutions** | 2869/ 2964 | 96.8%  [96.1%, 97.4%] | 14358/ 14388 | 99.8%  [99.7%, 99.9%] | 17227/ 17352 | 99.3%  [99.1%, 99.4%] |

^1^Variants detected include variants classified as VUS and benign

These results demonstrate robustness of the FoundationOne Liquid CDx DNA extraction process across KingFisher instruments, extraction reagent lots, and cancer types.

##### 9.18 Guard Banding/Robustness

This validation study evaluated the impact on FoundationOne Liquid CDx test performance due to potential process variation with regard to uncertainty in the measurement of DNA concentration. This guard banding evaluation assessed the DNA input into each of the main process steps of the FoundationOne Liquid CDx assay (LC, HC, and sequencing).

Guard bands were evaluated relative to calculated process variability for LC, HC, and sequencing. The assessment of multiple DNA input levels into LC demonstrated robust performance and tolerance of various DNA input levels. The observed results of HC guard banding showed that the HC process is robust within the predefined specifications 1000ng to 2000ng of DNA input into HC. For sequencing, the observed distribution of coverage indicated robust performance within the predefined specifications of 1.0nM of DNA input concentration into sequencing (as summarized in **Table 31**).

**Table 31. Summary of process pass and failure rate at each guard banding DNA input level**

| **Process^1^** |  | **Input Level** | |  | **# of Pass** | **Pass Rate (%)** |
| --- | --- | --- | --- | --- | --- | --- |
| HC | -50% |  |  | 500ng | 18/20 | 90 |
|  | -20% |  |  | 800ng | 20/20 | 100 |

|  | Lower limit | 1000ng | 20/20 | 100 |
| --- | --- | --- | --- | --- |
|  | Upper limit | 2000ng | 20/20 | 100 |
|  | +20% | 2400ng | 20/20 | 100 |
|  | +50% | 3000ng | 18/20 | 90 |
| Sequencing | -50% | 0.5nM | 20/20 | 100 |
|  | -20% | 0.8nM | 20/20 | 100 |
|  | Normal input | 1.0nM | 20/20 | 100 |
|  | +20% | 1.2nM | 20/20 | 100 |
|  | +50% | 1.5nM | 20/20 | 100 |

^1^ Results for guardbanding of LC input levels can be found in **Table 32** below.

A second guard banding study was conducted to evaluate the impact of a range of cfDNA input masses (50% below the lower limit and 33% above the upper limit) for FoundationOne Liquid CDx using an updated LC input range (20-60ng). Results from this second study are described in **Table 32** and **Table** **33.** All 105 sample replicates tested in this study passed processing and post-sequencing metric specifications as shown in **Table 32** below. The results demonstrate robust performance across the intended DNA input range.

**Table 32. Processing Success Rates by cfDNA Input Level for FoundationOne Liquid CDx**

| **Process QC** | **cfDNA Input Level** | **cfDNA Input (ng)** | **# Total** | **# Pass** | **# Fail** | **Success Rate** | **95% Two-sided Score CI** |
| --- | --- | --- | --- | --- | --- | --- | --- |
| **LC** | -50% | 10 | 21 | 21 | 0 | 100% | [84.54%, 100%] |
|  | Lower limit | 20 | 21 | 21 | 0 | 100% | [84.54%, 100%] |
|  | Mid-point | 40 | 21 | 21 | 0 | 100% | [84.54%, 100%] |
|  | Upper limit | 60 | 21 | 21 | 0 | 100% | [84.54%, 100%] |
|  | +33% | 80 | 21 | 21 | 0 | 100% | [84.54%, 100%] |
| **HC** | -50% | 10 | 21 | 21 | 0 | 100% | [84.54%, 100%] |
|  | Lower limit | 20 | 21 | 21 | 0 | 100% | [84.54%, 100%] |
|  | Mid-point | 40 | 21 | 21 | 0 | 100% | [84.54%, 100%] |
|  | Upper limit | 60 | 21 | 21 | 0 | 100% | [84.54%, 100%] |
|  | +33% | 80 | 21 | 21 | 0 | 100% | [84.54%, 100%] |
| **Sequencing** | -50% | 10 | 21 | 21 | 0 | 100% | [84.54%, 100%] |
|  | Lower limit | 20 | 21 | 21 | 0 | 100% | [84.54%, 100%] |
|  | Mid-point | 40 | 21 | 21 | 0 | 100% | [84.54%, 100%] |
|  | Upper limit | 60 | 21 | 21 | 0 | 100% | [84.54%, 100%] |
|  | +33% | 80 | 21 | 21 | 0 | 100% | [84.54%, 100%] |
| **Post-sequencing QC** | -50% | 10 | 21 | 21 | 0 | 100% | [84.54%, 100%] |
|  | Lower limit | 20 | 21 | 21 | 0 | 100% | [84.54%, 100%] |
|  | Mid-point | 40 | 21 | 21 | 0 | 100% | [84.54%, 100%] |
|  | Upper limit | 60 | 21 | 21 | 0 | 100% | [84.54%, 100%] |
|  | +33% | 80 | 21 | 21 | 0 | 100% | [84.54%, 100%] |

**Table 33. Aggregate Percent Agreement Across All Targeted Variants per cfDNA Input Level for FoundationOne Liquid CDx**

| **cfDNA Input Level** | **cfDNA Input (ng)** | **Agreement (# Variants Detected / Total # Variants)**  **[95% Two-sided Score CI]** |
| --- | --- | --- |
| -50% | 10 | 92.86% (117/126) [86.98%, 96.2%] |
| Lower limit | 20 | 99.21% (125/126) [95.64%, 99.86%] |
| Mid-point | 40 | 100% (126/126) [97.04%, 100%] |
| Upper limit | 60 | 100% (126/126) [97.04%, 100%] |
| +33% | 80 | 100% (126/126) [97.04%, 100%] |

##### 9.19 Pan-Tumor Performance

A large-scale retrospective analysis was performed to demonstrate consistent test performance of FoundationOne Liquid CDx across samples derived from patients with different tumor types. This was evaluated by comparing in-process QC metrics across tumor types using historical data from samples processed in Foundation Medicine’s clinical laboratory using two prior versions of the FoundationOne

Liquid CDx assay. The FoundationOne Liquid CDx assay was developed based on two versions of the FoundationOne Liquid Laboratory Developed Test (LDT) assay, each of which targeted a subset of the genomic regions targeted by FoundationOne Liquid CDx. FoundationACT (FACT) targeted 62 genes and FoundationOne Liquid targeted 70 genes. The workflow is substantially similar between the assays. In order to support the use of historical data in this study, the regions commonly baited by the two previous assay versions and by FoundationOne Liquid CDx were evaluated for comparability of test performance (Section 2.15). The sample set for this analysis included 19,868 distinct samples from 25 tumor type categories that had previously been tested using the Foundation Medicine FoundationOne Liquid and FoundationACT assays, previous versions of FoundationOne Liquid CDx. **Table 34** below includes a summary of the tissue types included in the study. Overall, 98.1% of samples yielded ≥25ng DNA, which corresponds to a DNA input mass of 20ng for LC. A total of 89.1% of samples yielded ≥36ng of DNA which corresponds to a DNA input mass of 30ng for LC. The proportion of samples with an LC yield greater than the minimum mass of 500ng and lower than the maximum mass of 27000ng was 99.9%, with one sided 95% confidence interval of [99.8%, 99.9%]. The proportion of samples with an HC yield greater than the minimum mass of 20ng and lower than the maximum mass of 2250ng was 100%, with one sided 95% confidence interval of [99.99%, 100%]. The proportion of samples which met coverage requirements was 96.1%, with one sided 95% confidence interval of [95.9%, 96.3%]. The proportion of samples which met post-sequencing requirements was 95.6%, with one sided 95% confidence interval of [95.3%, 95.8%]. The proportion of samples that generated a passing or qualified (overall pass as results are reported) result after sequencing was 91.7%, with one- sided 95% confidence interval of [91.4%, 92.1%].

**Table 34. FoundationOne Liquid/FACT samples per tumor type and pass rates**

| **Tumor Type** | **Sample Size** | **DNA**  **Extraction**  **Pass Rate**  **(≥25 ng^2^)** | **DNA**  **Extraction**  **Pass Rate**  **(≥36 ng^1^)** | **LC**  **Yield**  **Pass**  **Rate** | **HC**  **Yield**  **Pass**  **Rate** | **Median**  **Coverage**  **Pass Rate** | **Post- sequencing**  **Pass Rate** | **Overall Pass**  **Rate (≥36 ng^1^)** | **Overall**  **Pass Rate**  **(≥25 ng^2^)** |
| --- | --- | --- | --- | --- | --- | --- | --- | --- | --- |
| Biliary Cancer | 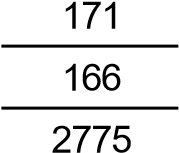 | 99.4% | 95.3% | 100.0% | 100.0% | 98.8% | 97% | 97.5% | 95.9% |
| Bladder Cancer |  | 97.6% | 85.5% | 100.0% | 100.0% | 93.2% | 98.7% | 95.8% | 92% |
| Breast Cancer |  | 97.6% | 87.7% | 99.9% | 100.0% | 96.4% | 95.5% | 95.8% | 91.9% |
| Cholangio- carcinoma | 377 | 98.9% | 96.0% | 99.7% | 100.0% | 98.7% | 97.3% | 97% | 95.7% |
| Colorectal  Cancer (CRC) | 1640 | 98.5% | 92.4% | 99.9% | 100.0% | 97.5% | 96.9% | 96.1% | 94.3% |
| Endocrine-Neuro Cancer | 75 | 100.0% | 85.3% | 100.0% | 100.0% | 100.0% | 93.3% | 96.9% | 93.3% |
| Endometrial Cancer | 231 | 98.3% | 88.3% | 100.0% | 100.0% | 96.5% | 95.9% | 95.1% | 92.5% |
| Esophagus Cancer | 291 | 99.7% | 92.4% | 100.0% | 100.0% | 97.6% | 96.5% | 96.3% | 94.1% |
| Glioma Cancer | 59 | 94.9% | 72.9% | 100.0% | 100.0% | 100.0% | 76.8% | 86% | 76.8% |
| **Tumor Type** | **Sample Size** | **DNA**  **Extraction**  **Pass Rate**  **(≥25 ng^2^)** | **DNA**  **Extraction**  **Pass Rate**  **(≥36 ng^1^)** | **LC**  **Yield**  **Pass**  **Rate** | **HC**  **Yield**  **Pass**  **Rate** | **Median**  **Coverage**  **Pass Rate** | **Post- sequencing**  **Pass Rate** | **Overall Pass**  **Rate (≥36 ng^1^)** | **Overall**  **Pass Rate**  **(≥25 ng^2^)** |
| Head and Neck Cancer | 154 | 96.1% | 81.8% | 100.0% | 100.0% | 89.2% | 96.2% | 95.2% | 85.8% |
| Kidney Cancer | 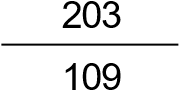 | 99.0% | 87.7% | 100.0% | 100.0% | 95.0% | 95.3% | 94.9% | 90.5% |
| Liver Cancer |  | 98.2% | 95.4% | 100.0% | 100.0% | 100.0% | 95.3% | 95.2% | 95.3% |
| Lung Non-Small  Cell Lung  Carcinoma  (NSCLC) | 5919 | 98.2% | 88.8% | 99.8% | 100.0% | 95.5% | 95.6% | 94.7% | 91.1% |
| Melanoma | 257 | 96.5% | 79.8% | 100.0% | 100.0% | 92.7% | 93.5% | 93.7% | 86.7% |
| Ovary Cancer | 496 | 97.8% | 88.5% | 100.0% | 100.0% | 95.9% | 94.6% | 94.5% | 90.7% |
| Pancreas Cancer | 1359 | 98.8% | 94.0% | 99.9% | 100.0% | 97.8% | 95.8% | 95% | 93.6% |
| Peripheral  Nervous System (PNS) | 44 | 100.0% | 90.9% | 100.0% | 100.0% | 100.0% | 93.2% | 95% | 93.2% |
| Prostate Cancer | 1778 | 97.3% | 87.7% | 99.9% | 100.0% | 96.9% | 95.1% | 95.8% | 92.1% |
| Rare Tumors | 1164 | 97.0% | 86.4% | 99.9% | 100.0% | 93.8% | 94.3% | 93.4% | 88.4% |
| Small Cell Cancer | 135 | 98.5% | 93.3% | 100.0% | 100.0% | 99.2% | 99.2% | 98.4% | 98.5% |
| Soft Tissue Sarcoma | 130 | 97.7% | 83.1% | 100.0% | 100.0% | 95.3% | 91.7% | 94.4% | 87.4% |
| Stomach Cancer | 267 | 98.9% | 89.1% | 100.0% | 100.0% | 98.1% | 93.8% | 95.8% | 92% |
| Thyroid Cancer | 50 | 98.0% | 86.0% | 100.0% | 100.0% | 100.0% | 81.6% | 90.7% | 81.6% |
| Unspecified | 856 | 98.5% | 89.1% | 100.0% | 100.0% | 95.5% | 96.6% | 96.3% | 92.3% |
| Unknown  Primary  Carcinoma  (CUP) | 1162 | 98.1% | 89.7% | 100.0% | 100.0% | 95.2% | 95.9% | 94.8% | 91.3% |

1. 36 ng of extracted cfDNA allows for sufficient cfDNA to process 30 ng of cfDNA
2. 25 ng of extracted cfDNA allows for sufficient cfDNA to process 20 ng of cfDNA

**Table 35** summarizes the overall sample pass rate across tumor types as well as performance metrics from key QC points in the process. These results demonstrate comparable test performance across tumor types.

| **QC Metric** | **QC Pass Rate Across Tumor Types^1^** | **Tumor Types with ≥ 90% QC Pass Rate** |
| --- | --- | --- |
| Overall report Pass/Qualified rate | 76.8%~98.5% | 24/25 (96%)^2^ |
| Library Construction | 99.7%~100% | 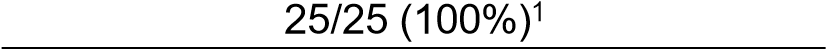  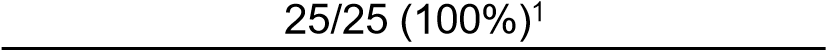  24/25 (96%)^1^ |
| Hybridization Capture | 100% |  |
| Median exon coverage | 89.2%~100% |  |
| Post-sequencing | 76.8%~99.2% | 23/25 (92%)^1^ |


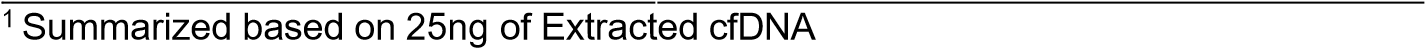
**Table 35. Summary of FoundationOne Liquid/FACT sample data**

^2^ Summarized based on 36ng of Extracted cfDNA

##### 9.20 Concordance – FoundationOne Liquid LDT to FoundationOne Liquid CDx

In order to support the use of historical data from the FoundationOne Liquid LDT to evaluate performance across cancer types, a study was performed to evaluate concordance between FoundationOne Liquid CDx and the FoundationOne Liquid LDT across the genomic regions targeted by both assays. This study evaluated the concordance of 927 unique samples processed on both the FoundationOne Liquid LDT and FoundationOne Liquid CDx assays. A total of 3,366 alterations, consisting of only those in common between the assays were evaluated. The concordance analysis using FoundationOne Liquid LDT or FoundationOne Liquid CDx as the reference assay is summarized by variant category in **Table 36**.

**Table 36. Concordance between FoundationOne Liquid LDT (F1L LDT) and FoundationOne Liquid CDx**

| **Variant/ Mutation Type** | **F1LCDx+ F1L LDT+** | **F1LCDx-**  **F1L LDT+** | **F1LCDx+ F1L LDT-** | **F1LCDx-**  **F1L LDT -** | **PPA [95% CI]** | **NPA [95% CI]** | **OPA [95% CI]** |
| --- | --- | --- | --- | --- | --- | --- | --- |
| **All Short Variants** | 2871 | 123 | 32 | 1171180 | 95.9%  [95.1%-96.6%] | >99.9%  [>99.9%-100.0%] | >99.9%  [>99.9%-100.0%] |
| **Base**  **Substitutions** | 2415 | 104 | 31 | 999032 | 95.9%  [95.0%-96.6%] | >99.9%  [>99.9%-100.0%] | >99.9%  [>99.9%-100.0%] |
| **Indels** | 456 | 19 | 1 | 172148 | 96.0%  [93.8%-97.6%] | >99.9%  [>99.9%-100.0%] | >99.9%  [>99.9%-100.0%] |
| **Rearrangements** | 147 | 20 | 24 | 59587 | 88.0%  [82.1%-92.5%] | >99.9%  [>99.9%-100.0%] | 99.9%  [99.9%-99.9%] |
| **Copy Number Amplifications** | 173 | 32 | 0 | 59463 | 84.4%  [78.7%-89.1%] | 99.8%  [>99.9%-100.0%] | 99.8%  [>99.9%-100.0%] |
| **Total** | 3191 | 175 | 166 | 1290230 | 94.8%  [94.0%-95.5%] | >99.9%  [>99.9%-100.0%] | >99.9%  [>99.9%-100.0%] |

The overall PPA between FoundationOne Liquid LDT and FoundationOne Liquid CDx assays, with FoundationOne Liquid LDT as the reference assay, was 94.8% with a 95% two-sided CI of [94.0%- 95.5%]. The respective short variant, rearrangement, and copy number amplification PPA values, with 95% two-sided CI, were: 95.9% [95.1%-96.6%], 88.0% [82.1%-92.5%], and 84.4% [78.7%-89.1%].

These results support the agreement between FoundationOne Liquid LDT and FoundationOne Liquid CDx and the applicability of the tumor comparability analysis performed using historical FoundationOne Liquid data.

##### 9.21 Molecular Index Barcode Performance

To evaluate the molecular index barcode performance, a total of 7,641 sequenced samples from FoundationOne Liquid CDx validation studies were analyzed with the FoundationOne Liquid CDx assay.

The overall coefficient of variation (% CV) of sequencing coverage across all barcodes was 8.95% for the enhanced sensitivity regions and 7.64% for the standard sensitivity regions. This observed small % CV includes both sample variability and barcode variability as these two components were confounded and inseparable. Results demonstrated that all 480 barcodes analyzed are detectable with low differences in sample coverage variance between barcodes, indicating comparable performance of the barcodes.

##### 9.22 Automation Line Equivalence

An intermediate precision study was performed to establish equivalence between the Hamilton instrumentation and the Biomek/Bravo instrumentation. The study consisted of eight contrived samples run in triplicate across four runs and both instrumentation platforms resulting in a total of 192 sample replicates included in the study overall. The analysis evaluated the negative call rate (NCR) and positive call rate (PCR) for 1,309 variants from eight contrived samples. The PCR and NCR were also evaluated by the seven variant categories.

The Mann-Whitney test was used for the comparison of PCR and NCR across liquid handling platforms for each sample, all samples in aggregate, and for each variant type. The NCR across platforms for each analysis set (per sample, all samples in aggregate, per variant type) were not statistically significant (p >0.05). by sample and by variant type. The PCR across platforms were not statistically significant (p >0.05) with the exception of contrived sample #3, the aggregate of all samples, and substitutions in a non-repetitive region or a repetitive region of ≤7 base pairs. The PCRs for the Hamilton liquid handling platform were slightly higher than the PCRs for the Biomek/Bravo platform (92.08% versus 90.15% for sample #3, 90.75% versus 89.67% for all samples, and 91.14% versus 90.10% for substitutions in a non-repetitive region or repetitive region of ≤7 base pairs). The statistical significance observed was due to large sample sizes allowing for the detection of slight differences that are likely not meaningful in practice; therefore, the Hamilton and Biomek/Bravo liquid handling platforms are considered to be interchangeable in the FoundationOne Liquid CDx assay.

##### 9.23 Updated LC Method Comparison Study

A method comparison study was conducted to demonstrate comparable performance between FoundationOne Liquid CDx assay using original and updated LC input ranges. Eighty-one clinical cfDNA samples from 10 unique disease ontologies were processed in triplicate to create 243 sample replicates. Samples were processed at the lower range for cfDNA input, 30ng for the original recommended minimum for LC input and 20ng for the updated minimum for LC input. 1,815 unique targeted variants were analyzed including CDx variants and variants from all alteration sub-types.

For each of the 81 samples, two of the three replicates were processed with FoundationOne Liquid CDx around a 30ng input level, using the previous LC method, (referred to as CCD_1_ and CCD_2_) and the third replicate was processed with FoundationOne Liquid CDx around a 20ng input level, using the updated LC method, (referred to as UCD_ALL_). The 243 sample replicates tested in this study passed all QC metrics. A non-inferiority analysis was performed. Aggregated PPA and NPA across all 1,815 targeted variants were calculated for pairwise comparisons between CCD_1_ and CCD_2_. PPAs and NPAs for all targeted variants were also calculated for either CCD_1_ or CCD_2_ versus UCD_ALL_. Agreement differences were calculated with corresponding 95% upper 1-sided bounds. The upper bounds of the

1-sided 95% CIs for agreement differences 𝜁_𝑃𝑃𝐴1_, 𝜁_𝑃𝑃𝐴2_, 𝜁_𝑁𝑃𝐴1_ and 𝜁_𝑁𝑃𝐴2_ were all <1% for UCD_ALL_. Therefore, the

FoundationOne Liquid CDx assay using the updated LC input range was demonstrated to be noninferior to FoundationOne Liquid CDx using the original LC input range for the detection of CDx and non-CDx variants.

##### 9.24 Updated F1LCDx LC and HC Processes

Analytical validation studies were performed to support the updated LC and HC workflow to the F1LCDx assay. Two of the studies performed (Method Comparison and Limit of Blank) are detailed below in **Sections 9.24.1** and **9.24.2**)

**9.24.1 Method Comparison Study**

This study evaluated the LC and HC workflow updates to the F1LCDx assay compared to the originally approved F1LCDx test configuration. Seventy-eight (78) unique cfDNA specimens from a wide variety of tumor types were tested in this study using a non-inferiority (NI) study design. Three replicates of each of these 78 cfDNA specimens were prepared (234 sample replicates in total): CCD_1_ and CCD_2_ (two replicates; representing the original test configuration), and UCD (one replicate; representing the updated test configuration with LC/HC improvements). In total, 229 out of 234 CCD/UCD replicates were sequenced successfully for an overall success rate of 97.86%.

For the primary NI analysis, a total of 437 unique targeted variants (including both targeted CDx and targeted non-CDx variants) were analyzed. Targeted variants are defined as variants reported when detected by F1LCDx that have a result ≥ 1x LoD in either CCD1 or CCD2. Non-targeted variants are any other variants detected and reported by F1LCDx, either by CCD_1_ or CCD_2,_ including variants with levels below 1x LoD for which the sample was not selected.

Aggregated PPAs and NPAs across all targeted variants were estimated for the pair-wise comparisons between CCD_1_ and CCD_2_, and between UCD and CCD with CCD_1_ or CCD_2_ as reference and calculated separately. The results of aggregated agreements and differences between aggregated agreements (𝜁_𝑃𝑃𝐴1_, 𝜁_𝑃𝑃𝐴2_, 𝜁_𝑁𝑃𝐴1_, 𝜁_𝑁𝑃𝐴2_) with corresponding 95% upper 1-sided confidence interval (CI) bounds are provided in **Table 37.**

**Table 37. Non-Inferiority Testing of the Aggregated Agreement Results for Targeted Variants**

| **NI Statistics** | **CCD_1_ & CCD_2_ Agreement (%)** | **UCD & CCD**  **Agreement (%)** | **Agreement**  **Difference ζ (%)** | **Upper bound of 95% one-sided CI for ζ^1^ (%)** |
| --- | --- | --- | --- | --- |
| **ζPPA1 = (PPAC1C2 - PPAC1U)** | 96.77 | 96.58 | 0.19 | 1.38 |
| **ζPPA2 = (PPAC2C1 - PPAC2U)** | 95.86 | 97.36 | -1.51 | -0.08 |
| **ζNPA1 = (NPAC1C2 - NPAC1U)** | 99.93 | 99.93 | 0.00 | 0.03 |
| **ζNPA2 = (NPAC2C1 - NPAC2U)** | 99.95 | 99.96 | -0.01 | 0.02 |

^1^CIs for agreement differences were calculated based on Newcombe 1988 method for paired data

The upper bounds of the 1-sided 95% CIs for agreement differences 𝜁𝑃𝑃𝐴1, 𝜁𝑃P𝐴2, 𝜁𝑁P𝐴1 and 𝜁𝑁𝑃𝐴2 were all <1.5%. Aggregated PPAs and NPAs across all tumor profiling variants (includes targeted and non-targeted variants) were also estimated for the pair-wise comparisons between CCD1 and CCD2, and between UCD and CCD with CCD1 or CCD2 as reference and calculated separately. The results of aggregated agreements and differences between aggregated agreements (𝜁𝑃𝑃𝐴1, 𝜁𝑃𝑃𝐴2, 𝜁𝑁𝑃𝐴1, 𝜁𝑁𝑃𝐴2) with corresponding 95% upper 1-sided confidence interval (CI) bounds are provided in **Table 38.**

**Table 38. Non-Inferiority Testing of the Aggregated Agreement Results for Reportable Tumor Profiling Variants**

| **NI Statistics** | **CCD_1_ & CCD_2_ Agreement (%)** | **UCD & CCD Agreement (%)** | **Agreement**  **Difference ζ (%)** | **Upper bound of 95% one-sided CI for ζ^1^ (%)** |
| --- | --- | --- | --- | --- |
| **ζPPA1 = (PPAC1C2 - PPAC1U)** | 88.41 | 88.12 | 0.29 | 2.11 |
| **ζPPA2 = (PPAC2C1 - PPAC2U)** | 81.77 | 86.60 | -4.83 | -2.70 |
| **ζNPA1 = (NPAC1C2 - NPAC1U)** | 99.71 | 99.85 | -0.14 | -0.11 |
| **ζNPA2 = (NPAC2C1 - NPAC2U)** | 99.83 | 99.93 | -0.10 | -0.08 |

The upper bounds of the 1-sided 95% CIs for agreement differences 𝜁𝑃𝑃𝐴1, 𝜁𝑃𝑃𝐴2, 𝜁𝑁𝑃𝐴1 and 𝜁𝑁𝑃𝐴2 were all <2.11%. Concordance analyses were performed for tumor profiling variants by short variant type, i.e., substitutions, insertions, and deletion (**Table 39**), The discordances observed are due to low level VAF (≤1%), however the level of concordance is comparable between CCD1 and CCD2, and between UCD and CCD1 and CCD2 indicating that the updated F1LCDx assay is non-inferior for calling tumor profiling substitutions, insertions, and deletion variants.

**Table 39. Aggregated Agreement Results for Reportable Tumor Profiling Short Variants by Variant Subtype**

| **Aggregated Agreement** | **Point Estimate (%) (Numerator/Denominator) [Two-sided 95% Score CI (%)]** | | |
| --- | --- | --- | --- |
|  | **Substitution** | **Insertion** | **Deletion** |
| 𝛼𝛼**PPAC1C2** | 89.87 (275/306) [85.98,  92.77] | 78.26 (54/69) [67.18, 86.36] | 87.57 (162/185) [82.04, 91.57] |
| 𝛼𝛼**NPAC1C2** | 99.74 (23868/23930) [99.67, 99.80] | 99.78 (5030/5041) [99.61, 99.88] | 99.62 (14506/14561) [99.51, 99.71] |
| 𝛼𝛼**PPAC1U** | 90.85 (278/306) [87.09,  93.59] | 72.46 (50/69)  [60.95, 81.61] | 88.11 (163/185) [82.65, 92.01] |
| 𝛼𝛼**NPAC1U** | 99.85 (23894/23930) [99.79, 99.89] | 99.86 (5034/5041) [99.71, 99.93] | 99.82 (14535/14561) [99.74, 99.88] |
| 𝛼𝛼**PPAC2C1** | 81.60 (275/337) [77.12,  85.38] | 83.08 (54/65)  [72.18, 90.28] | 74.65 (162/217) [68.47, 79.98] |
| 𝛼𝛼**NPAC2C1** | 99.87 (23868/23899) [99.82, 99.91] | 99.70 (5030/5045) [99.51, 99.82] | 99.84 (14506/14529) [99.76, 99.89] |
| 𝛼𝛼**PPAC2U** | 88.61 (319/360) [84.91,  91.49] | 82.35 (56/68) [71.64, 89.61] | 81.17 (181/223) [75.52, 85.75] |
| 𝛼𝛼**NPAC2U** | 99.93 (25518/25536) [99.89, 99.96] | 99.94 (5389/5392) [99.84, 99.98] | 99.92 (15520/15533) [99.86, 99.95] |

To demonstrate that the updated F1LCDx is non-inferior to the originally approved F1LCDx for copy number alterations reported by the test as listed in **Table 1** and **Table 3** above, 2 samples with *BRCA2* homozygous deletions, and 7 samples positive for *ERBB2* amplifications, including 4 samples with copy number amplification near the threshold for positivity were tested. All 9 samples were concordant among all replicates tested (UCD and CCD_1_ and CCD_2_), indicating that the updated F1LCDx assay is non-inferior for calling copy number alterations approved for the device.

To demonstrate that the updated F1LCDx is non-inferior to the originally approved F1LCDx for rearrangements and fusions reported by the test as listed in Table 1 and 3, 1 sample with a *ROS1* fusion, and 5 samples positive for *NTRK1/2/3* fusions/rearrangements, 4 samples positive for *ALK* fusions/rearrangements, 4 samples with *ATM* rearrangements and 5 samples with *BRCA1/2* rearrangements were evaluated. All *ROS1* fusion and NTRK1/2/3 fusion/rearrangement samples with valid results were concordant among all replicates tested (UCD and CCD_1_ and CCD_2_). Among the 4 *ALK* fusion/rearrangements samples tested, only 1 was concordant among CCD_1_ and CCD_2,_ however the variant was not detected in the UCD replicate. All four samples had low fusion read levels near threshold required for positivity. Similarly for *ATM* and *BRCA1/2* rearrangements of the 9 samples positive for these alterations, only 3 samples (1 *ATM* and 2 *BRCA2* rearrangements) were concordant across all three replicate (UCD and CCD_1_ and CCD_2_), while the remaining were discordant among UCD and CCD_1_ and CCD_2_ to a similar extend. The reason for the discordance were attributed low read support.

**9.24.2 Limit of Blank Study**

The study evaluated the LoB of the updated F1LCDx assay using LC and HC improvements. Two plasma cfDNA replicates and one replicate of donor-matched gDNA from 47 donors with no known cancer diagnosis, who were categorized in both age and smoking status cohorts, were collected, extracted, and tested, with 24 out of the 47 donors tested with one reagent lot, and the remaining 23 donors tested with another reagent lot. Of the 94 cfDNA replicates and the paired 47 gDNA replicates, all replicates passed QC specifications.

Therefore, all 47 donors and their matched gDNA and cfDNA replicates were used in the statistical analysis. False positive rates (FPRs) were computed with variants in the universal set in technical blank replicates for each Category reported by F1LCDx and on a per sample replicate basis. Technical blanks replicate results represent variant results obtained after removing variants detected in a patient’s buffy coat which are likely to represent germline variants. However, it should be noted that F1LCDx does not normalize patient samples with their germline DNA. The universal set U consists of all unique variants detected in previous F1LCDx validation studies up until January 2022, and all variants detected in this LoB study. The LoB study results obtained in the technical blank samples are summarized in **Table 40**

below.

**Table 40. LoB Study Results in Technical Blanks**

| **Variant Category** | **# of detected variants**  **across all source**  **samples**  **(n=47) in a variant level** | **Total # of unique variants for a variant level in the**  **universal set ×**  **Total # of source samples** | **False**  **Positive Rate (%)** | **# of detected variants across all sample replicates**  **(n=94) in a variant category** | **Per Sample**  **Replicate False**  **Positive Rate (%)**  **[number of replicates with at least one detected**  **variant/total sample**  **replicates)** |
| --- | --- | --- | --- | --- | --- |
| Category 1:  *ATM* 2921+1G>C | 1 | 42,488 | 0.0024 | 2 | 2.13% [2/94] |
| Category 1:  *BRAF* 1799T>A | 1 | 42,488 | 0.0024 | 1 | 1.06% [1/94] |
| Category 1:  All **other*** CDx variants  noted in Table 1 of Intended Use | 0 | 42,488 | 0.0000 | 0 | 0.00% [0/94] |
| Category 2: cfDNA  Biomarkers with Strong  Evidence of Clinical  Significance in cfDNA | 0 | 47 | 0.0000 | 0 | 0.00% [0/94] |
| Category 3: Biomarkers with  Evidence of Clinical Significance in tissue supported by:  3A: strong analytical validation using cfDNA  3B: analytical validation using  cfDNA | 1 | 14,335 | 0.0070 | 1 | 1.06% [1/94] |
| Category 4: Other Biomarkers with Potential Clinical Significance** | 24 | 338,353 | 0.0071 | 31 | 30.85% [29/94] |

*Note that the two false positives observed in Category 1, identified as ATM 2921+1G>C and BRAF 1799T>A, are excluded from the false positive rate calculation of ‘All other CDx variants’ category.

**False positive results may be due to background signals inherent in sequencing methods designed for high sensitivity, clonal hematopoiesis of indeterminate potential, or germline polymorphisms.

All CDx variants had false positive rate (FPR) < 5% and 2 CDx variants were detected at VAFs below the 0.40% VAF LoD for SNVs and indels in F1LCDx (2 calls for *ATM*_2921+1G>C in paired replicates from the same donor: VAF = 0.12% and 0.13%; a single call for *BRAF*_1799T>A: VAF = 0.10%). Across all four categories reported by F1LCDx, FPR of on a per variant per sample replicate ranged from 1.06% (1 variant call out of 94 valid cfDNA replicates) to 2.13% (2 variant calls out of 94 valid sample replicates) for non-companion diagnostic variants (Categories 2-4 of the tumor profiling variants reported).No rearrangements or copy number alterations were detected in any of the four categories.

Since a large number of non-CDx SNV and indel variants are detected in technical blanks at low VAF levels (less than 0.5% VAF for SNVs and 1% VAF for indels), i.e., in plasma from donors that do not have solid neoplasms after subtracting the presumably germline variants detected in the donors buffy coat, there is a risk that variants detected at very low allele frequency may be a false positive result. This risk of false positivity identified based on the LoB study results in the updated F1LCDx is the same as the risk identified for originally approved F1LCDx, refer to **Section 9.9**. This risk may be due to background signals inherent in sequencing methods designed for high sensitivity. They may also be due to CHIP or. Additional clinical investigation to confirm the presence of the variant in the patient’s tumor with another FDA approved or cleared test is strongly recommended.

**9.24.3 HC Yield Pass Rate**

Confirmation of HC yield pass rate utilized samples and replicates from two LC/HC validation studies (Method Comparison and Limit of Blank). Given a total of 172 samples/replicates across the 2 studies, 172 samples/replicates passed HC yield specification (≥38.5 ng and <600 ng), with an overall HC yield pass rate of 100%, which passed the acceptance criteria of 90%. These results demonstrate that the updated F1LCDx assay using Twist Fast Hyb performs per product requirements.

#### 10 Clinical Validation Studies

##### 10.1 Clinical Bridging Study: Detection of ALK Rearrangements to Determine Eligibility for Treatment with Alectinib

The clinical validity of using FoundationOne Liquid CDx as a companion diagnostic to identify patients with non- small cell lung cancer (NSCLC) harboring *ALK* rearrangements for treatment with alectinib was assessed through a clinical bridging study using screening (i.e., pre-alectinib treatment) plasma samples from Cohort A of the Blood First Assay Screening Trial (BFAST, BO29554).

The BFAST trial is a Phase II/III multicenter study, in which Cohort A evaluated the safety and efficacy of alectinib as a treatment for patients with advanced or metastatic NSCLC who tested positive for an *ALK* rearrangement as determined by a blood-based NGS clinical trial assay (CTA).

The concordance between FoundationOne Liquid CDx and the CTA was evaluated as summarized in **Table 41**.

**Table 41. Concordance between FoundationOne Liquid CDx and the CTA for the detection of ALK rearrangements**

|  | **CTA Pos** | **CTA Neg** | **Total** |
| --- | --- | --- | --- |
| **FoundationOne Liquid CDx Positive**^1^ | 63 | 0 | 63 |
| **FoundationOne Liquid CDx Negative** | 12 | 174 | 186 |
| **Missing** | 4 | 9 | 13 |
| **Total** | 79 | 183 | 262 |

^1^ VAF values down to 0.06%VAF were observed for *ALK* rearrangements.

The PPA and NPA between FoundationOne Liquid CDx and the CTA using the CTA as the reference for the primary analysis set and the corresponding 95% confidence intervals were:

- PPA [95% CI]: 84.0% [73.7%, 91.4%]
- NPA [95% CI]: 100% [ 97.9%, 100.0%]

After adjusting for a 5% prevalence of *ALK* rearrangements in the intended use population, the PPV and NPV calculated using the CTA as the reference and the corresponding 95% confidence intervals were:

- PPV [95% CI]: 100.0% [94.3%, 200.0%]
- NPV [95% CI]: 93.5% [89.0%, 96.6%]

The estimated Overall Response Rate (ORR) and the corresponding 95% confidence intervals was 88.9% [78.4%, 95.4%] for the FoundationOne Liquid CDx *ALK*-positive population which is comparable with the observed ORR and the corresponding 95% confidence intervals of 87.4% [78.5%, 93.5%] for the CTA *ALK*- positive population (BFAST Cohort A).

A sensitivity analysis was performed to estimate the clinical efficacy of treating patients with alectinib when considering missing FoundationOne Liquid CDx results. The estimated ORR and the corresponding 95% confidence intervals were 90.4% [90.1%, 90.6%] for the patient population that are both CTA *ALK*+ and FoundationOne Liquid CDx *ALK*+, demonstrating the robustness of the clinical efficacy analysis to missing FoundationOne Liquid CDx results.

##### 10.2 FoundationOne Liquid CDx Concordance Study for EGFR exon 19 deletion and EGFR exon 21 L858R Alteration

Clinical validity of FoundationOne Liquid CDx assay was established as a companion diagnostic to identify patients with advanced NSCLC who may be eligible for treatment with TARCEVA® (erlotinib), IRESSA® (gefitinib), or TAGRISSO® (osimertinib). Two hundred and eighty retrospective samples from NSCLC patients were included in this study, which were tested for *EGFR* exon 19 deletion and exon 21 L858R alterations (*EGFR* alterations) by the FoundationOne Liquid CDx assay and the previously approved **cobas**® *EGFR* Mutation Test v2 (Roche Molecular Systems, referred to as cobas assay). Both *EGFR* alteration-positive and *EGFR* alteration-negative samples (based on CTA results) were selected from the screen failed population of an unrelated clinical trial in NSCLC. To avoid selection bias, the samples were selected starting with a specific testing date until the predefined number of 150 *EGFR* alteration-positive and 100 *EGFR* alteration-negative samples were fulfilled. Samples were tested across two replicates by the cobas assay (denoted as CCD1 and CCD2) and one replicate by FoundationOne Liquid CDx. The tested samples, from NSCLC patients, were compared against the intended use (IU) population with respect to gender to ensure the screening population is representative of the IU population. The variant calls were evaluated based on the agreement between both the FoundationOne Liquid CDx and the cobas assay results and between the two cobas assay replicates. For any samples in which there was insufficient plasma to process both CCD1 and CCD2, processing was not performed. In total there were 177 samples with complete test results available for analysis. The agreement analysis results between FoundationOne Liquid CDx and the cobas assay for the detection of *EGFR* exon 19 deletions and L858R alterations are presented in **Table 42**.

**Table 42. Agreement analysis results for EGFR exon 19 deletion and L858R separately.**

| **Exon 19 deletion** | PPAC1F | 95.5% | NPAC1F | 95.6% |
| --- | --- | --- | --- | --- |
|  | PPAC1C2 | 97.7% | NPAC1C2 | 98.9% |
|  | PPAC2F | 95.5% | NPAC2F | 96.0% |
|  | PPAC2C1 | 96.2% | NPAC2C1 | 99.4% |
| **L858R** | PPAC1F | 100.0% | NPAC1F | 95.6% |
|  | PPAC1C2 | 92.9% | NPAC1C2 | 98.9% |
|  | PPAC2F | 100.0% | NPAC2F | 94.7% |
|  | PPAC2C1 | 96.0% | NPAC2C1 | 98.0% |

The concordance of *EGFR* mutations as detected by FoundationOne Liquid CDx and the cobas assay were assessed and the data are summarized in **Table 43**.

**Table 43. Concordance among CCD1, CCD2 and FoundationOne Liquid CDx results with eligible samples (n=177)**

|  | **CCD1+** | |  |  | **CCD1-** |  |
| --- | --- | --- | --- | --- | --- | --- |
|  | **CCD2+** | **CCD2-** | **Total** | **CCD2+** | **CCD2-** | **Total** |
| **FoundationOne Liquid CDx+** | 80 | 4 | 84 | 1 | 3 | 4 |
| **FoundationOne Liquid CDx-** | 2 | 0 | 2 | 0 | 87 | 87 |
| **Total** | 82 | 4 | 86 | 1 | 90 | 91 |

The agreement analysis results between FoundationOne Liquid CDx and the cobas assay are presented in **Table 44.**

**Table 44. Agreement analysis results**

|  | **PPA** | **NPA** |
| --- | --- | --- |
| CCD2\|CCD1^1^ | 95.3% | 98.9% |
| CCD1\|CCD2^2^ | 96.1% | 98.7% |
| FoundationOne Liquid CDx\|CCD1* | 97.7% | 95.6% |
| FoundationOne Liquid CDx\|CCD2** | 97.7% | 95.4% |

^1^CCD1: the 1st replicate of cobas assay as the reference

^2^CCD2: the 2nd replicate of cobas assay as the reference

The estimates of ζPPA1, ζPPA2, ζNPA1 and ζNPA2 and the corresponding one-sided 95% upper bounds confidence limit computed using the bootstrap method are presented in **Table 45.**

**Table 45. Point estimate and one-Sided 95% upper confidence limit of ζPPA1, ζNPA1, ζPPA2, and ζNPA**

|  | **Point Estimate** | **Mean one-sided 95% upper confidence limit** |
| --- | --- | --- |
| **ζPPA1** | -2.3% | 2.3% |
| **ζNPA1** | 3.3% | 6.6% |
| **ζPPA2** | -1.6% | 4.7% |
| **ζNPA2** | 3.3% | 6.6% |

Based on these results, FoundationOne Liquid CDx has been demonstrated to be non-inferior to the cobas assay for the detection of *EGFR* exon 19 deletions and *EGFR* exon 21 L858R mutations. This study establishes the clinical validity of the FoundationOne Liquid CDx assay for identifying patients eligible for treatment with erlotinib, gefitinib, and osimertinib.

##### 10.3 Clinical Bridging Study: Detection of BRCA1/BRCA2/ATM Alterations to Determine Eligibility for Treatment with olaparib

The clinical validity of using FoundationOne Liquid CDx as a companion diagnostic to identify patients with metastatic castrate-resistant prostate cancer (mCRPC) harboring *BRCA1, BRCA2 or ATM* alterations for treatment with olaparib was assessed through a clinical bridging study using screening (i.e., pre-olaparib treatment) plasma samples from Cohort A of the PROfound trial.

The PROfound trial is a Phase III, open label, randomized study to assess the efficacy and safety of olaparib (Lynparza™) versus enzalutamide or abiraterone acetate in men with metastatic castration- resistant prostate cancer who have failed prior treatment with a new hormonal agent and have homologous recombination repair gene mutations. Only Cohort A patients with either *BRCA1*, *BRCA2* or *ATM* mutations were tested with the FoundationOne Liquid CDx assay.

In total, 4,425 patients were screened and 387 (9.6%) were randomized into the PROfound study by the CTA. Of these 387 patients, 245 patients were randomized in cohort A. In cohort A, 181 out of the 245 randomized patients both consented to the use of their sample for ctDNA CDx development and had a plasma sample available for testing. In total, 181/245 (73.9%) of the Cohort A patients were tested using the FoundationOne Liquid CDx assay. Of these, 139 (76.8%) Cohort A patients had a successful FoundationOne Liquid CDx test result and 42 Cohort A patients had a failed FoundationOne Liquid CDx test result. This represents 56.7% (139/245) of total Cohort A patients with a FoundationOne Liquid CDx result. In addition, 250 non-HRRm patient samples were randomly selected for ctDNA testing from the screen-failed population to determine the NPA/NPV of the FoundationOne Liquid CDx assay. A total of 194/250 (77.6%) screen failed non-HRRm patients were successfully tested using the FoundationOne Liquid CDx assay.

Of the 139 successfully tested Cohort A patients, 111 patients were reported as *BRCA1/BRCA2/ATM* mutation positive and 28 randomized patients were reported as biomarker negative by FoundationOne Liquid CDx.

Therefore, the FoundationOne Liquid CDx ctDNA biomarker positive subgroup comprises 111 patients with *BRCA1, BRCA2, and/or ATM* mutations.

**Table 46. Sample accountability for olaparib clinical bridging study**

| **Description** | **Number of patients** |
| --- | --- |
| Patients randomized into PROfound | 387 |
| Patients with qualifying *BRCA1, BRCA2,* or *ATM* alterations (Cohort A) | 245 |
| Cohort A patients with samples tested by FoundationOne Liquid CDx | 181 |
| FoundationOne Liquid CDx results available | 139 |
| Cohort A patients, biomarker positive by FoundationOne Liquid CDx | 111 |

**Table 47** shows the agreement analysis between CLIA CTA (tissue test) and the FoundationOne Liquid CDx results for PROfound patients, including Invalid and Not Tested results.

**Table 47. Summary of agreement analyses for FoundationOne Liquid CDx compared against CTA tissue test**

|  |  | **CTA Results (n=495)** | |
| --- | --- | --- | --- |
|  |  | **Biomarker positive** | **Biomarker negative** |
| **FoundationOne Liquid** | **Biomarker positive^1^** | 111 | 16 |

| **CDx assay** | **Biomarker^2^ negative** | 28 | 178 |
| --- | --- | --- | --- |
|  | **Biomarker^3^ Invalid** | 42 | 56 |
|  | **Not Tested** | 64 | 0 |
| **Agreement analyses (only Valid results included)** | **PPA (95% CI^3^)** | 79.9 (72.2, 86.2) [111/139] |  |
|  | **NPA (95% CI^3^)** | 91.8 (87.0, 95.2) [178/194] |  |
|  | **OPA (95% CI^3^)** | 86.8 (82.7, 90.2) [289/333] |  |
|  | **PPV (95% CI^3^)** | 66.6 (56.0, 77.2) |  |
|  | **NPV (95% CI^3^)** | 95.7 (94.3, 97.1) |  |

^1^ VAF values down to 0.11%VAF were observed for short variants and 0.25% VAF for rearrangements in *BRCA1, BRCA2*, or *ATM*. ^2^ Biomarker refers to patients with eligible *BRCA/ATM* mutations

^3^ Confidence intervals calculated using Clopper-Pearson method

The PPA and NPA between FoundationOne Liquid CDx and the CTA using the CTA as the reference for the primary analysis set and the corresponding 95% confidence intervals were:

- PPA [95% CI]: 79.9% [72.2%, 86.2%]
- NPA [95% CI]: 91.8% [87.0%, 95.2%]

After adjusting for a 17.1% prevalence of *BRCA1/2* and *ATM* alterations in the intended use population, the PPV and NPV calculated using the CTA as the reference and the corresponding 95% confidence intervals were:

- PPV [95% CI]: 66.6% [56.0%, 77.2%]
- NPV [95% CI]: 95.7% [94.3%, 97.1%]

The estimated radiological progression-free survival (rPFS) hazard ratio (HR) and the corresponding 95% confidence intervals were 0.331 [0.21, 0.53] for the FoundationOne Liquid CDx biomarker positive population, which were comparable with the observed rPFS HR and the corresponding 95% confidence intervals of 0.34 [0.25, 0.47] for the CTA biomarker positive population (PROfound Cohort A).

Sensitivity analysis to evaluate the robustness of the clinical efficacy estimate against the unknown FoundationOne Liquid CDx results was performed using the multiple imputation method in All Patients. After imputing the missing FoundationOne Liquid CDx results, the median rPFS HR and corresponding [95% CI] across the imputed datasets was 0.44 [0.32, 0.59], demonstrating robustness of the analysis to missing FoundationOne Liquid CDx results.

##### 10.4 Clinical Bridging Study: Detection of BRCA1 and BRCA2 Alterations to Determine Eligibility of mCRPC Patients for Treatment with rucaparib

The clinical performance of FoundationOne Liquid CDx as a companion diagnostic to identify patients with metastatic castration-resistant prostate cancer (mCRPC) harboring breast cancer gene 1 or 2 (*BRCA1* or *BRCA2*) alterations for treatment with rucaparib was demonstrated using pre-rucaparib treatment blood samples from clinical trial NCT0952534 (TRITON2). The clinical data supporting the use of rucaparib in the proposed indication was submitted as New Drug Application (NDA) 209115/S- 004.

A bridging study was conducted to evaluate: 1) the concordance between *BRCA1* and *BRCA2* alteration status by the CTA and FoundationOne Liquid CDx, and 2) the clinical efficacy of rucaparib treatment in patients that would be eligible for therapy based on *BRCA1* and *BRCA2* alteration status as determined by FoundationOne Liquid CDx. A total of 209 patients (All Patients) from TRITON2 were included in NDA 209115/S-004. Genomic status was determined using the FoundationOne LDT (F1 LDT), the FoundationOne Liquid LDT, or a local test, as summarized in **Figure 1**.


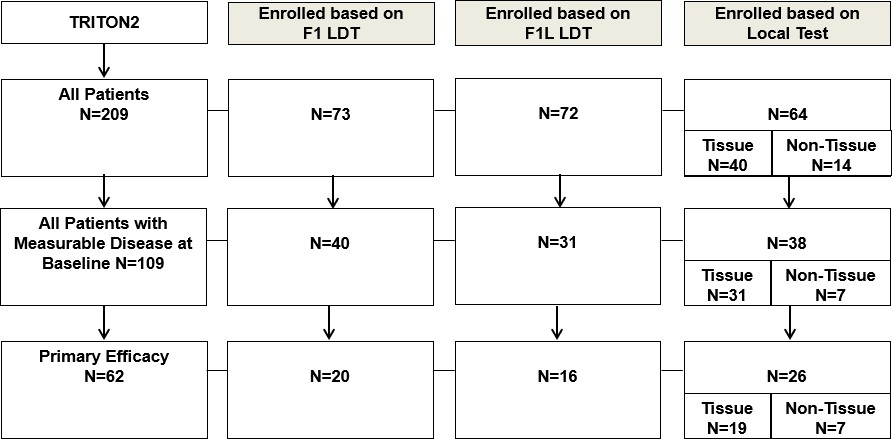


Figure 1: TRITON2 Patient Enrollment

Pre-rucaparib treatment plasma samples were available for 92% (192/209) of the patients. FoundationOne Liquid CDx data were available for 93% (178/192) of the patients with samples tested; inadequate input material resulted in FoundationOne Liquid CDx test data being unavailable for 14 patients. In total, FoundationOne Liquid CDx data were available for 85% (178/209) of All Patients.

Of the 62 patients in the Primary Efficacy Population (those patients with measurable visceral and/or nodal disease at baseline), FoundationOne Liquid CDx test data were obtained for 84% (52/62) and used for concordance and efficacy analyses. The sample accountability for this clinical bridging study is summarized in **Table 48**.

Table 48. Sample accountability for rucaparib prostate clinical bridging study

| **Description** | **Number** |
| --- | --- |
| All Patients in TRITON2 | 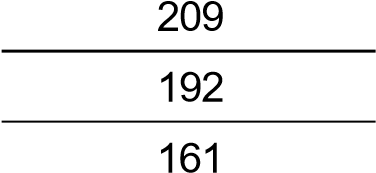 |
| Total samples available for retesting by FoundationOne Liquid CDx |  |
| Patients with evaluable FoundationOne Liquid CDx data and cfDNA input ≥ 30ng (All Patients) |  |
| Patients with evaluable FoundationOne Liquid CDx test results and cfDNA input ≥ 20ng (All  Patients) | 178 |
| Primary efficacy population in TRITON2 | 62 |
| Patients with evaluable FoundationOne Liquid CDx test results and cfDNA input ≥ 30ng (Primary  Efficacy Population) | 48 |
| Patients with evaluable FoundationOne Liquid CDx test results and cfDNA input ≥ 20ng (Primary  Efficacy Population) | 52 |

Concordance between FoundationOne Liquid CDx and the CTAs

The concordance of *BRCA* status between FoundationOne Liquid CDx and CTA test results were evaluated in all patients as summarized in **Table 49** and **Table 50**.

Table 49. Concordance between FoundationOne Liquid CDx BRCA Status and the CTA BRCA Status in All Patients with FoundationOne Liquid CDx cfDNA input ≥30ng

| **All Patients** | |  | **CTA** |  |
| --- | --- | --- | --- | --- |
|  |  | ***BRCA* Positive** | ***BRCA* Negative** | **Total** |
| **FoundationOne**  **Liquid CDx** | ***BRCA* Positive^1^** | 75 | 1 | 76 |
|  | ***BRCA* Negative** | 16 | 69 | 85 |
|  | ***BRCA* Unknown** | 2 | 1 | 3 |
|  | **Total** | 93 | 71 | 164 |

^1^ VAF values down to 0.15%VAF were observed for short variants and 0.85%VAF for rearrangements in *BRCA1* or *BRCA2.*

The PPA, NPA between FoundationOne Liquid CDx and the CTA, based on a cfDNA input ≥30ng, were determined using the CTA as the reference for all patients.

- PPA (95% CI): 82.4% (73.0%, 89.6%)
- NPA (95% CI): 98.6% (92.3%, 100.0%)

Table 50. Concordance between FoundationOne Liquid CDx BRCA Status and the CTA BRCA Status in All Patients with FoundationOne Liquid CDx cfDNA input ≥20ng

| **All Patients** | |  | **CTA** |  |
| --- | --- | --- | --- | --- |
|  |  | ***BRCA* Positive** | ***BRCA* Negative** | **Total** |
| **FoundationOne Liquid CDx** | ***BRCA* Positive^1^** | 82 | 1 | 83 |
|  | ***BRCA* Negative** | 18 | 77 | 95 |
|  | ***BRCA* Unknown** | 3 | 2 | 5 |
|  | **Total** | 103 | 80 | 183 |

^1^ VAF values down to 0.15%VAF were observed for short variants and 0.85%VAF for rearrangements in *BRCA1* or *BRCA2*.

The PPA, NPA between FoundationOne Liquid CDx and the CTA, based on a cfDNA input ≥20ng, were determined using the CTA as the reference for all patients.

- PPA (95% CI): 82.0% (73.1%, 89.0%)
- NPA (95% CI): 98.7% (93.1%, 100%)

Efficacy Based on FoundationOne Liquid CDx Results

*BRCA1* and *BRCA2* alteration status were verified retrospectively by FoundationOne Liquid CDx in 66% (41/62) of the patients in the Primary Efficacy Population. The ORR [95% CI] in the Primary Efficacy Population was 46.3% [30.7%-62.6%] in *BRCA* positive patients determined by FoundationOne Liquid CDx, which is comparable to the ORR of 43.5% [31.0%-56.7%] in patients identified by CTA (**Table 51**).

Table 51. ORR in the primary efficacy population by CTA and FoundationOne Liquid CDx test results

| **Primary Efficacy Population** | **FoundationOne Liquid CDx** |  | **CTA** |
| --- | --- | --- | --- |
|  | ***BRCA* Positive**  **N=38**  **(≥ 30 ng cfDNA input)** | ***BRCA* Positive**  **N = 41**  **(≥ 20 ng cfDNA input)** | ***BRCA* Positive N = 62** |
| **Confirmed ORR (CR + PR), n (%)** | 18 (47.4) | 19 (46.3) | 27 (43.5) |
| 95% CI(%) | 31.0 – 64.2 | 30.7 - 62.6 | 31.0 – 56.7 |

ORR = objective response rate; CR = complete response; PR = partial response.

Sensitivity analysis to evaluate the robustness of the clinical efficacy estimate against the unknown FoundationOne Liquid CDx results was performed using the multiple imputation method and demonstrated that the drug efficacy in the FoundationOne Liquid CDx positive population was robust to missing FoundationOne Liquid CDx results.

##### 10.5 Clinical Bridging Study: Detection of PIK3CA Alterations to Determine Eligibility for Treatment with alpelisib

Clinical validity of using FoundationOne Liquid CDx to identify breast cancer patients harboring *PIK3CA* alterations eligible for treatment with alpelisib was assessed through retrospective testing of plasma samples collected prior to study treatment from advanced or metastatic breast cancer patients enrolled in clinical trial CBYL719C2301 (SOLAR-1). A total of 395 patients were enrolled based on CTA1 results and 177 patients were enrolled based on CTA2 results. All 395 patients enrolled based on CTA1 results were retrospectively tested by CTA2. This clinical bridging study was performed based on CTA2 results.

Samples with ≥30 ng from 375 patients were tested by FoundationOne Liquid CDx. Excluding those with invalid results for either CTA2 or CDx (4 and 12, respectively), the primary efficacy analyses were conducted using data from the 359 subjects who were CTA2-evaluable and CDx-evaluable **Table 52.**

**Table 52. Concordance between FoundationOne Liquid CDx and CTA2**

|  |  |  | **CTA2** |  |  |
| --- | --- | --- | --- | --- | --- |
| **CDx** | **Positive** | **Negative** |  | **Invalid** | **Total** |
| **Positive** | 165 | 0 |  | 1 | 166 |
| **Negative** | 65 | 129 |  | 3 | 197 |
| **Invalid** | 7 | 5 |  | 0 | 12 |
| **Total** | 237 | 134 |  | 4 | 375 |

^1^ VAF values down to 0.14%VAF were observed for short variants in *PIK3CA*. Samples not tested are excluded from the analysis. Samples tested with cfDNA input <30 ng are excluded from the analysis.

The point estimates of PPA and NPA between FoundationOne Liquid CDx and the CTA2 assay and the corresponding 95% confidence intervals were:

- PPA [95% CI]: 71.7% [65.4%, 77.5%]
- NPA [95% CI]: 100% [97.2%, 100%]

The primary efficacy analysis in the *PIK3CA* alteration positive population identified by FoundationOne Liquid CDx was based on PFS by local investigator assessment per Response Evaluation Criteria in Solid Tumors (RECIST) 1.1 criteria. Clinical efficacy of alpelisib in combination with fulvestrant for the

FoundationOne Liquid CDx-positive population with cfDNA input ≥30 ng (N=165) was demonstrated with an estimated 54% risk reduction in disease progression or death in the alpelisib plus fulvestrant arm compared to the placebo plus fulvestrant arm (HR = 0.46, 95% CI: 0.30, 0.70).

As summarized in **Table 53**, the PFS hazard ratio for the 165 tissue CTA2-positive, FoundationOne Liquid CDx- positive patients was 0.46 (95% CI: 0.30, 0.70). Median PFS was 11.0 months for the alpelisib plus fulvestrant arm versus 3.6 months for the placebo plus fulvestrant arm.

**Table 53. Progression-free survival in the CTA2-positive, FoundationOne Liquid CDx-positive patients (primary analysis set)**

| **Progression free survival (months)** | **Alpelisib 300mg qd + Fulvestrant N=84** | **Placebo qd +**  **Fulvestrant N=81** | **HR (95% CI) Alpelisib 300mg qd + Fulv /Placebo qd + Fulv^1^** |
| --- | --- | --- | --- |
| No of events (%) | 54 (64.3) | 67 (82.7) | 0.46 (0.30, 0.70) |
| PD (%) | 52 (61.9) | 61 (75.3) |  |
| Death (%) | 2 (2.4) | 6 (7.4) |  |
| No of censored (%) | 30 (35.7) | 14 (17.3) |  |
| Median (95% CI)^2^ | 11.0 (7.3, 15.9) | 3.6 (2.4, 5.8) |  |

1. Hazard ratio (HR) estimated using Cox regression model stratified by the two stratification factors: presence of lung and/or liver metastases, previous treatment with any CDK4/6 inhibitor, and adjustedfor clinically relevant covariates, as well as the imbalanced covariates.
2. The 95% CI calculated from PROC LIFETEST output using the method of Brookmeyer and Crowley (1982). CDx results from samples tested with cfDNA input <30 ng are treated as missing.

PD = progressive disease

Sensitivity analysis to evaluate the robustness of the clinical efficacy estimate against the missing FoundationOne Liquid CDx results was performed using the multivariate imputation by chained equations (MICE0 method. After imputing the missing FoundationOne Liquid CDx results, the hazard ratio was estimated to be 0.63 (95% CI: 0.45, 0.87), demonstrating robustness of the clinical efficacy analysis to missing FoundationOne Liquid CDx results.

##### 10.6 Clinical Bridging Study: Detection of MET single nucleotide variants (SNVs) and indels that lead to MET exon 14 skipping to Determine Eligibility for Treatment with capmatinib

The clinical performance of FoundationOne Liquid CDx for detecting SNVs and indels that lead to *MET* exon 14 skipping in NSCLC patients who may benefit from treatment with capmatinib (**Table 1**) was established with clinical data generated from a clinical bridging study using samples from patients enrolled in the GEOMETRY mono-1 study. The study demonstrates concordance between the enrollment assay, i.e., CTA, and the FoundationOne Liquid CDx assay and establish the effectiveness of the FoundationOne Liquid CDx assay.

GEOMETRY mono-1 was a prospectively designed, multi-center, open-label, single arm Phase II study of oral cMET inhibitor, TABRECTA (capmatinib), in adult patients with *EGFR* wild-type (wt), and anaplastic lymphoma kinase (ALK) negative advanced NSCLC. Patients were enrolled into multiple cohorts of the study, but the bridging study was focused on the fully-enrolled *MET* exon 14 skipping positive Cohorts 4 and 5b. Cohort 4 only enrolled pretreated (second and third line) patients with *MET* exon 14 skipping, and Cohort 5b only enrolled treatment-naïve patients with *MET* exon 14 skipping. Patients were screened for enrollment into Cohorts 4 and 5b for *MET* exon 14 skipping status using a *MET* exon 14 skipping reverse-transcriptase PCR (RT-PCR) CTA that was detected *MET* exon 14 skipping in a patient’s tissue. Plasma samples were collected and stored prior to study treatment for retrospective testing. Patients enrolled in Cohorts 4 and 5b received 400mg of capmatinib orally twice daily in tablet form. Efficacy was evaluated every six weeks from the first day of treatment until RECIST 1.1 disease progression.

A clinical bridging study was conducted to evaluate: 1) the concordance between *MET* single nucleotide variants (SNVs) and indels that lead to *MET* exon 14 skipping status by the CTA and FoundationOne Liquid CDx, and 2) the clinical efficacy of capmatinib treatment in patients that would be eligible for therapy based on *MET* biomarker positive status as determined by FoundationOne Liquid CDx.

The primary endpoint of GEOMETRY mono-1 was the ORR by Blinded Independent Review Committee (BIRC) assessment by cohort to determine whether treatment with capmatinib is effective. Duration of response (DOR) as assessed by BIRC was the key secondary endpoint.

The primary concordance analysis of the status of *MET* SNVs and indels that led to *MET* exon 14 skipping between FoundationOne Liquid CDx and the tissue CTA test results were evaluated in both analysis sets that met ≥30 ng cfDNA input and ≥20 ng cfDNA input. The analysis on the ≥30 ng cfDNA input population evaluated 150 patients (78 *MET* exon 14 skipping positive patients, and 72 *MET* exon 14 skipping negative patients), excluding invalid CDx results. The analysis on the ≥20 ng cfDNA input population evaluated 171 patients (83 *MET* exon 14 skipping positive patients, and 88 *MET* exon 14 skipping negative patients), excluding invalid CDx results.

Agreement (PPA, NPA and OPA) for combined Cohort 4 and 5b by ≥30 ng cfDNA input and ≥20 ng cfDNA input CDx are shown in **Table 54** and **Table 55**, below. For the 150 patients meeting the ≥30 ng cfDNA input, the PPA, NPA and OPA and respective confidence intervals were determined to be 70.5% (59.1%, 80.3%), 100% (95.0%, 100%) and 84.7% (77.9%, 90.0%). For the 171 patients meeting the

≥20 ng cfDNA input, the PPA, NPA and OPA and respective confidence intervals were determined to be 68.7% (57.6%, 78.4%), 100% (95.9, 100%) and 84.8% (78.5%, 89.8%).

**Table 54. Agreement between CDx and CTA based on CTA results in combined cohorts by cfDNA input** **> 30 ng**

| Cohort 4 and Cohort 5b  (CDx sample requirement: cfDNA input ≥ 30 ng) | **Measure of agreement** | **Percent agreement % (n/N)** | **95% CI (1)** |
| --- | --- | --- | --- |
|  | PPA^1^ | 70.5 (55/ 78) | (59.1, 80.3) |
|  | NPA | 100 (72/ 72) | (95.0, 100) |
|  | OPA | 84.7 (127/150) | (77.9, 90.0) |

﻿1 VAF values down to 0.16%VAF were observed for MET short variants.

N: The total number of patients. It is the denominator for percentage (%) calculation n: Number of patients with agreement between CTA and CDx

(1) The 95% CI calculated using Clopper-Pearson method

| Cohort 4 and Cohort 5b  (CDx sample requirement: cfDNA input ≥20 ng) | **Measure of agreement** | **Percent agreement % (n/N)** | **95% CI (1)** |
| --- | --- | --- | --- |
|  | PPA^1^ | 68.7 (57/ 83) | (57.6, 78.4) |
|  | NPA | 100 (88/ 88) | (95.9, 100) |
|  | OPA | 84.8 (145/171) | (78.5, 89.8) |

**Table 55. Agreement between CDx and CTA based on CTA results in combined cohorts by cfDNA input ≥20 ng**

1 VAF values down to 0.16%VAF were observed for MET short variants.

N: The total number of patients. It is the denominator for percentage (%) calculation n: Number of patients with agreement between CTA and CDx

(1) The 95% CI calculated using Clopper-Pearson method

Based on the PPA of 70.5% (59.1%, 80.3%) between FoundationOne Liquid CDx and the tissue CTA, reflex testing using tissue specimens to an FDA approved tissue test is recommended, if feasible, if the plasma test is negative.

Clinical effectiveness of FoundationOne Liquid CDx was evaluated by estimation of clinical efficacy in the CTA-enrolled *MET* exon 14 deletion positive patient population, as assessed by the primary objective of ORR by BIRC. The GEOMETRY mono-1 clinical trial met its primary objective demonstrating a statistically significant improvement in ORR by BIRC assessments in patients with *MET* exon 14 deletion positive tumors in each cohort.

**Table 56** and **Table 57** present the clinical efficacy of TABRECTA analyzed in CTA-positive patients who were tested as CDx positive (“double positive” patients) in each cohort that met the ≥30 ng cfDNA input and ≥20 ng cfDNA input CDx sample requirements, respectively. In Cohort 4 there were 39 patients with ≥30 ng cfDNA input and 41 with ≥20 ng cfDNA input with valid results for analysis of ORR. In Cohort 5b there were 16 patients, all of whom met the ≥30 ng cfDNA input.

Patients in Cohort 4 that met the ≥30 ng cfDNA input demonstrated an ORR of 51.3% (34.8%, 67.6%). Patients from Cohort 4 that met the ≥20 ng cfDNA input requirements demonstrated an ORR of 48.8%

(32.9%, 64.9%). For patients in Cohort 5b, all patients met the ≥30 ng cfDNA input and demonstrated an ORR of 81.3% (54.4%, 96.0%).

|  | **(CTA+, CDx+)**  **CDx sample requirements** | | | |  | |
| --- | --- | --- | --- | --- | --- | --- |
|  | **cfDNA input ≥ 30 ng N=39** | | **cfDNA input ≥ 20 ng N=41** | | **CTA+ N=69** | |
|  | **n (%)** | **95% CI (1)** | **n (%)** | **95% CI (1)** | **n (%)** | **95% CI (1)** |
| Overall Response Rate (ORR: CR + PR) | 20 (51.3) | (34.8, 67.6) | 20 (48.8) | (32.9, 64.9) | 28 (40.6) | (28.9, 53.1) |

**Table 56. Overall response per BIRC assessment in (CTA-positive, CDx-positive) and CTA- positive patients by cohort and CDx sample requirements (Cohort 4)**

(1) The 95% CI calculated using Clopper-Pearson method

**Table 57. Overall response per BIRC assessment in (CTA-positive, CDx-positive) and CTA- positive patients by cohort and CDx sample requirements (Cohort 5b).**

|  | **(CTA+, CDx+)**  **CDx sample requirements** | | | |  | |
| --- | --- | --- | --- | --- | --- | --- |
|  | **cfDNA input ≥ 30 ng N=16** | | **cfDNA input ≥ 20 ng N=16** | | **CTA+ N=28** | |
|  | **n (%)** | **95% CI (1)** | **n (%)** | **95% CI (1)** | **n (%)** | **95% CI (1)** |
| Overall Response Rate (ORR: CR + PR) | 13 (81.3) | (54.4, 96.0) | 13 (81.3) | (54.4, 96.0) | 19 (67.9) | (47.6, 84.1) |

(1) The 95% CI calculated with the Clopper-Pearson Exact method.

Estimated drug efficacy in FoundationOne Liquid CDx Positive (F1LCDx(+)) patients

The ORR by BIRC assessment in F1LCDx(+) patients was calculated for Cohort 4 and Cohort 5b, separately. Because all CTA(-) patients are tested as negative by CDx (i.e. NPA=100%) and thus PPV is estimated as 100%, the results do not vary with Pr(CTA+) values and the ORR in F1LCDx(+) population is estimated as the same as the ORR in the FoundationOne Liquid CDx [CTA(+)/CDx(+)] population. For F1LCDx(+) patients meeting “Recommended” CDx sample requirement (cfDNA input ≥ 30 ng), the ORR (95% CI) is 51.3% (34.8%, 67.6%) in Cohort 4 and 81.3% (54.4%, 96.0%) in Cohort 5b, respectively. For CDx(+) patients meeting “Minimum” CDx sample requirement (cfDNA input ≥ 20 ng), the ORR (95% CI) is 48.8% (32.9%, 64.9%) in Cohort 4 and 81.3% (54.4%, 96.0%) in Cohort 5b, respectively.

Sensitivity analysis on missing FoundationOne Liquid CDx results

The impact of missing FoundationOne Liquid CDx results on the concordance between CTA and FoundationOne Liquid CDx and final drug efficacy in F1LCDx(+) patients was evaluated by imputing the missing FoundationOne Liquid CDx results using multiple imputation method. For Cohort 4, the imputed ORR (95% CI) by BIRC were estimated to be 46.5% (32.6%, 60.9%) given “Recommended” sample requirement and 47.2% (33.3%, 61.5%) given “Minimum” sample requirement. For Cohort 5b, the imputed ORRs and two-sided 95% CIs by BIRC were estimated to be 75.3% (53.3%, 94.4%) given

“Recommended” sample requirement and 78.1% (55.6%, 95.5%) given “Minimum” sample requirement. The sensitivity analysis results demonstrated that the concordance between CTA and FoundationOne Liquid CDx and final drug efficacy in F1LCDx(+) population are robust to missing FoundationOne Liquid CDx results.

##### 10.7 Clinical Bridging Study: Detection of ROS1 Fusions to Determine Eligibility for Treatment with entrectinib

The clinical performance of using FoundationOne Liquid CDx as a companion diagnostic to identify NSCLC patients harboring *ROS1* fusions eligible for treatment with entrectinib (**Table 1**) was assessed in this clinical bridging study. All available pre-entrectinib treatment plasma samples from patients enrolled in ALKA, STARTRK-1, and STARTRK-2 clinical trials were tested by FoundationOne Liquid CDx as part of this clinical bridging study. Only samples from STARTRK-2 were available for testing by FoundationOne Liquid CDx.

A clinical bridging study was conducted to evaluate the 1) the concordance between the FoundationOne Liquid CDx assay and the CTAs used for clinical trial enrollment for the detection of *ROS1* fusions and 2) the clinical efficacy of entrectinib treatment in patients who would be eligible for therapy based on *ROS1* fusions positive as determined by FoundationOne Liquid CDx.

A total of 255 patients were included in the clinical bridging study. Of these 255 patients, 161 were determined as *ROS1* fusion positive based on testing by the CTAs. Initially, the clinical bridging study included 51 *ROS1* fusion positive NSCLC patients from the new drug application (NDA) efficacy population, 41 additional *ROS1* fusion positive, *ROS1* inhibitor-naive patients with NSCLC with measurable disease who had insufficient follow-up (<12 months) at the time of the NDA submission, 67 *ROS1* fusion positive patients with NSCLC who were enrolled prior to October 31, 2018, and two patients with prior *ROS1* inhibitor treatment and used only for the concordance evaluation. In total, clinical outcome data from 161 *ROS1* fusion positive patients (as determined by the CTAs) enrolled before October 31, 2018 (based on the May 1, 2019 clinical data cutoff date) were planned for use in the bridging analysis. Of the 94 *ROS1* fusion negative samples (as determined by the CTAs), 73 were patients enrolled in the clinical trial by the CTAs as *NTRK1/2/3* fusion positive. The remaining 21 *ROS1* fusion negative samples were FFPE tissue-matched plasma samples procured from a commercial source, with tissue testing by one of the CTAs used for clinical trial enrollment. Only samples from STARTRK-2 were available for testing by FoundationOne Liquid CDx and, thus, 218 of the 255 samples were evaluated by retrospective FoundationOne Liquid CDx testing. Among them, 203 samples met the FoundationOne Liquid CDx quality control metrics, and 175 samples met the recommended sample input of cfDNA ≥ 30ng. An additional 28 samples met the minimum FoundationOne Liquid CDx sample input criteria of cfDNA ≥ 20ng. Sample accountability for this clinical bridging study is summarized in **Table 58**.

**Table 58. Sample Accountability for the ROS1 Clinical Bridging Study**

| **Source of samples** | **Total # of samples (n=255)** | **Sample fail/ unavailable**  **(n=52)** | **F1LCDx evaluable**  **(n=203)** | **DNA ≥ 30 ng (n=175)** | **DNA ≥ 20 ng and < 30 ng (n=28)** |
| --- | --- | --- | --- | --- | --- |
| **Procured *ROS1* Negative samples** | 21 | 2 | 19 | 17 | 2 |
| ***ROS1* Negative by CTA test*** | 73 | 14 | 59 | 51 | 8 |
| ***ROS1* Positive by CTA test** | 161 | 36 | 125 | 107 | 18 |
| **Total** | 255 | 52 (20.4%) | 203 (79.6%) | 175 (68.6%) | 28 (11.0%) |

*The CTA *ROS1*-fusion negative samples were enrolled in the clinical trials as CTA *NTRK*-fusion positive

The primary analyses were conducted for the 175 patients with evaluable FoundationOne Liquid CDx results that also had a DNA input of ≥ 30 ng. The concordance between FoundationOne Liquid CDx and the CTAs is summarized in **Table 59.** Over 20 different types of CTAs with a mix of technologies (RT-PCR, FISH, NGS) and analytes (RNA and DNA) were used to enroll the patients in the clinical trials.

**Table 59. Concordance result between FoundationOne Liquid CDx and CTA for the detection of ROS1fusions for samples with DNA content ≥30 ng (n=175).**

|  |  |  | **CTAs** |  |
| --- | --- | --- | --- | --- |
|  |  | **Detected** | **Not Detected** | **Total** |
| **F1LCDx** | **Detected** | 55 | 0 | 55 |
|  | **Not Detected** | 52 | 68 | 120 |
|  | **Unevaluable** | 54 | 26 | 80 |
|  | **Total** | 161 | 94 | 255 |
|  |  |  | **CTAs** |  |
|  |  | **Detected** | **Not Detected** | **Total** |
| **Agreement Statistics** | | PPA | NPA |  |
| **Excluding CDx-Unevaluable** | | 51.4% (55/107) | 100% (68/68) |  |
| **Results** | | 95% CI*: (42.05%, 60.66%) | 95% CI*: (94.65%, 100%) |  |
| **Percent Unevaluable** | | 33.5% (54/161)  95% CI*: (26.7%, 41.1%) | 27.7% (26/94)  95% CI*: (19.6%, 37.4%) |  |

*Calculated with Wilson two-sided 95% CI

The following concordance statistics were calculated for this sample set using the CTA as the reference:

- PPA [95% CI]: 51.4% [42.05%, 60.66%]
- NPA [95% CI]: 100.0% [94.65%, 100%]

After adjusting for a 1% prevalence of *ROS1* rearrangements in the intended use population PPV and NPV were calculated using the CTA as the reference:

- PPV [95% CI]: 100% [93.47%, 100%]
- NPV [95% CI]: 99.51% [99.41%, 99.61%]

The discordances between the CTAs and FoundationOne Liquid CDx among *ROS1* fusion positive patients was evaluated by stratifying the PPA into 2 subgroups, DNA-based NGS CTAs and RNA- based NGS CTAs. The PPA between FoundationOne Liquid CDx and DNA-based NGS CTAs was 55.6% (10/18) with 95% two-sided CI (33.7%, 75.4%). The PPA between FoundationOne Liquid CDx and RNA-based NGS CTAs was 50.6% (40/79) with 95% two-sided CI (39.8%, 61.4%). Of the 52 CTA positive patients who were FoundationOne Liquid CDx negative, 92.3% (48/52) did not have detectable tumor fraction as determined by FoundationOne Liquid CDx, suggesting that the ctDNA content in these samples was low.

The clinical efficacy of entrectinib in the clinical trials was measured in ORR with either confirmed complete response (CR) or partial response (PR) based on blinded independent centralized review (BICR). Only clinical samples with clinical outcome data were used in this part of the study analysis.

The ORR in the CTA-positive population was 67.3% (107/159) with 95% two-sided CI (59.7%, 74.1%). Fifty-four patients were CTA positive and had FoundationOne Liquid CDx *ROS1* fusion-positive results. The ORR for this population was 66.7% (36/54) with 95% two-sided CI (53.4%, 77.8%). Fifty-one patients were CTA positive but had FoundationOne Liquid CDx *ROS1* negative results. The ORR for this population was 66.7% (34/51) with 95% two-sided CI (53.0%, 78.0%).

Fifty-four patients were CTA positive but were unevaluable by FoundationOne Liquid CDx. The ORR for this population was 68.5% (37/54) with 95% two-sided CI (55.3%, 79.3%) (**Table 60**).

| **Clinical outcome** | **Total CTA positive population**  **(N=159)** | **CTA positive and F1LCDx positive (N=54)** | **CTA positive and F1LCDx negative (N=51)** | **CTA positive and**  **F1LCDx unevaluable (N=54)** |
| --- | --- | --- | --- | --- |
| **ORR%** [95% CI**] | 67.3% | 66.7% | 66.7% | 68.5% |
|  | [59.7%, 74.1%] | [53.4%, 77.8%] | [53.0%, 78.0%] | [55.3%, 79.3%] |
| Complete response | 14 (8.8%) | 5 (9.3%) | 6 (11.8%) | 3 (5.6%) |
| Partial response | 93 (58.5%) | 31 (57.4%) | 28 (54.9%) | 34 (63.0%) |
| Number of responders | **N=107** | **N=36** | **N=34** | **N=37** |
| **Duration of response** |  |  |  |  |

**Table 60. ORR in CTA-positive, FoundationOne Liquid CDx-positive patients**

| **Clinical outcome** | **Total CTA positive population**  **(N=159)** | **CTA positive and F1LCDx positive (N=54)** | **CTA positive and F1LCDx negative (N=51)** | **CTA positive and**  **F1LCDx unevaluable (N=54)** |
| --- | --- | --- | --- | --- |
| Median^±^ in months (range) | 9.5 (1.8, 42.3) | 6.4 (1.8, 20.5) | 13.4 (1.9, 27.6) | 11.1 (4.6, 42.3) |
| % with duration ≥9 months | 61.7% | 38.9% | 70.6% | 75.7% |
| % with duration ≥12 months | 41.1% | 19.4% | 55.9% | 48.6% |
| % with duration ≥18 months | 19.6% | 5.6% | 26.5% | 27.0% |

﻿**Two-sided 95% CI for each subgroup was based on the Wilson-score method

±Arithmetic median used (not Kaplan-Meier methods) since censoring data was not available

Sensitivity analysis to evaluate the robustness of the clinical efficacy estimate against the missing FoundationOne Liquid CDx results was performed using the multiple imputation method. Based on the 100 bootstrap samples with 50 times imputation estimated ORR of the FoundationOne Liquid CDx *ROS1*-positive population was 67.1% [50.7%, 78.9%].

There were 70 *ROS1* positive patients by the CTAs with partial or complete response to entrectinib, who also had an FoundationOne Liquid CDx result. Among them, only 51.4% (36/70) were positive by FoundationOne Liquid CDx (95% CI: 39.9, 62.8). There were 35 *ROS1*-positive patients by the CTAs who did not respond to entrectinb, who also had an FoundationOne Liquid CDx result (54-36=18 and

51-34=17). Among them, 51.4% (18/35) were positive by FoundationOne Liquid CDx (95% CI: 35.6, 67.0).

##### 10.8 Clinical Bridging Study: Detection of NTRK 1/2/3 Fusions to Determine Eligibility for Treatment with entrectinib

The clinical performance of using FoundationOne Liquid CDx as a companion diagnostic to identify patients with solid tumors harboring *NTRK1, NTRK2,* or *NTRK3* fusions eligible for treatment with entrectinib (**Table 1**) was assessed in this clinical bridging study. All patients with available plasma samples from the NDA population from ALKA, STARTRK-1, and STARTRK-2 clinical trials were tested by FoundationOne Liquid CDx as part of this clinical bridging study. Only samples from STARTRK-2 were available for testing by FoundationOne Liquid CDx.

A clinical bridging study was conducted to evaluate the 1) the concordance between the FoundationOne Liquid CDx assay and the CTAs used for clinical trial enrollment for the detection of *NTRK* fusions and 2) the clinical efficacy of entrectinib treatment in patients who would be eligible for therapy based on *NTRK* fusions positive as determined by FoundationOne Liquid CDx.

A total of 256 patients were included in the clinical bridging study. Of these 256 patients, 74 were determined as *NTRK* fusion-positive based on testing by the CTAs. Initially, the clinical bridging study included 54 *NTRK* fusion-positive patients from the NDA efficacy population, as well as 20 *NTRK* fusion-positive patients who were enrolled after the data cutoff. Of the 182 *NTRK* fusion-negative samples, 161 were patients enrolled in the clinical trial by the CTAs as *ROS1* fusion-positive. The remaining 21 *NTRK* fusion-negative samples were FFPE tissue-matched plasma samples procured from a commercial source, with tissue testing by one of the CTAs used for clinical trial enrollment. Only samples from STARTRK-2 were available for testing by FoundationOne Liquid CDx and, thus, 218 of the 256 samples were included for retrospective FoundationOne Liquid CDx testing. Among them, 203 samples met the FoundationOne Liquid CDx quality control metrics, and 175 samples met the recommended sample input of cfDNA ≥ 30ng. An additional 28 samples met the minimum FoundationOne Liquid CDx sample input criteria of cfDNA ≥ 20ng. Sample accountability for this clinical bridging study is summarized in **Table 61**.

**Table 61. Sample Accountability for the NTRK Clinical Bridging Study**

| **Source of samples** | **Total # of samples (n=256)** | **Sample fail/ unavailable**  **(n=53)** | **F1LCDx evaluable**  **(n=203)** | **DNA ≥30 ng (n=175)** | **DNA ≥20 ng and <30 ng**  **(n=28)** |
| --- | --- | --- | --- | --- | --- |
| **Procured *NTRK* Negative samples** | 21 | 2 | 19 | 17 | 2 |
| ***NTRK* Negative by CTA test*** | 161 | 36 | 125 | 107 | 18 |
| ***NTRK* Positive by CTA test** | 74 | 15 | 59 | 51 | 8 |
| **Total** | 256 | 53 (20.7%) | 203 (79.3%) | 175 (68.4%) | 28 (10.9%) |

*The CTA *NTRK*-fusion negative samples were enrolled in the clinical trial as CTA *ROS1*-fusion positive.

The primary analyses were conducted for the 175 patients with evaluable FoundationOne Liquid CDx results that also had a DNA input of ≥ 30 ng. A comparison of the clinical outcomes and baseline characteristics demonstrated that the FoundationOne Liquid CDx-evaluable population was representative of the FoundationOne Liquid CDx-unevaluable population in this bridging study. The concordance between FoundationOne Liquid CDx and the CTAs is summarized in **Table 62**. Over 20 different types of CTAs with a mix of technologies (RT-PCR, FISH, NGS) and analytes (RNA and DNA) were used to enroll the patients in the clinical trials.

**Table 62. Concordance between FoundationOne Liquid CDx and CTAs for the detection of NTRK1, NTRK2, and NTRK3 fusions**

|  |  |  | **CTAs** |  |
| --- | --- | --- | --- | --- |
|  |  | **Detected** | **Not Detected** | **Total** |
| **F1LCDx** | **Detected** | 25 | 0 | 25 |
|  | **Not Detected** | 26 | 124 | 150 |
|  | **Unevaluable** | 23 | 58 | 81 |
|  | **Total** | 74 | 182 | 256 |
| **Agreement Statistics Excluding CDx-Unevaluable Results** | | PPA: 49.0% (25/51)  95% CI*: (35.9%, 62.3%) | NPA: 100% (124/124) 95% CI*: (97.0%, 100%) |  |
| **Percent Unevaluable** | | 31.1% (23/74)  95% CI*: (21.7%, 42.3%) | 31.9% (58/182)  95% CI*: (25.5%, 39.0%) |  |

*Calculated with Wilson 2-sided 95% CI

The following concordance statistics were calculated for this sample set:

- PPA [95% CI]: 49.0% [35.9%, 62.3%]
- NPA [95% CI]: 100.0% [97.0%, 100%]

After adjusting for a 0.32% prevalence of *NTRK* fusions in the intended use population PPV and NPV were calculated using the CTA as the reference:

- PPV [95% CI]: 100% [86.7%,100%]
- NPV [95% CI]: 99.8% [99.79%, 99.88%]

The discordances between the CTAs and FoundationOne Liquid CDx among *NTRK1/2/3* fusion- positive patients was evaluated by stratifying the PPA into 2 subgroups, DNA-based NGS CTAs and RNA-based NGS CTAs. The PPA between FoundationOne Liquid CDx and DNA-based NGS CTAs was 65.0% (13/20) with 95% two-sided CI (43.3%, 81.9%). The PPA between FoundationOne Liquid CDx and RNA-based NGS CTAs was 38.7% (12/31) with 95% two-sided CI (23.7%, 56.2%).

The clinical efficacy of entrectinib in the clinical trials was measured in ORR with either confirmed CR or PR based on BICR. Only clinical samples with clinical outcome data were used in this part of the study analysis.

The ORR in the CTA positive population was 63.5% (47/74) with 95% two-sided CI (52.1%, 73.6%). Twenty-five (25) patients were CTA positive and had FoundationOne Liquid CDx *NTRK* positive results.

The ORR for this population was 72.0% (18/25) with 95% two-sided CI (52.4%, 85.7%). Twenty-six (26) patients were CTA positive but had FoundationOne Liquid CDx *NTRK* negative results. The ORR for this population was 57.7% (15/26) with 95% two-sided CI (38.9%, 74.5%).

23 patients were CTA positive but were FoundationOne Liquid CDx-unevaluable. The ORR for this population was 60.9% (14/23) with 95% two-sided CI (40.8%, 77.8%) ( **Table 63)**.

**Table 63. ORR in CTA-positive, FoundationOne Liquid CDx-positive patients**

| **Clinical outcome** | **Total CTA positive population (N=74)** | **CTA positive and**  **F1LCDx positive (N=25)** | **CTA positive and**  **F1LCDx negative (N=26)** | **CTA positive and**  **F1LCDx unevaluable (N=23)** |
| --- | --- | --- | --- | --- |
| **ORR%** [95% CI**] | 63.5% | 72.0% | 57.7% | 60.9% |
|  | [52.1,73.6] | [52.4, 85.7] | [38.9, 74.5] | [40.8, 77.8] |
| Complete response | 5 (6.8%) | 0 (0.0%) | 1 (3.8%) | 4 (17.4%) |
| Partial response | 42 (56.8%) | 18 (72.0%) | 14 (53.8%) | 10 (43.5%) |
| Number of responders | **N=47** | **N=18** | **N=15** | **N=14** |
| **Duration of response** |  |  |  |  |
| Median^±^ in months (range) | 7.5 (1.4, 26.0) | 5.9 (1.9, 16.6) | 7.9 (1.4, 26.0) | 8.3 (2.8, 25.9) |
| % with duration ≥9 months | 44.7% | 38.9% | 46.7% | 50.0% |
| % with duration ≥12 months | 29.8% | 22.2% | 40.0% | 28.6% |
| % with duration ≥18 months | 10.6% | 0.0% | 13.3% | 21.4% |

﻿**Two-sided 95% CI for each subgroup was based on the Wilson-score method

±Arithmetic median used (not Kaplan-Meier methods) since censoring data was not available

Sensitivity analysis to evaluate the robustness of the clinical efficacy estimate against the missing FoundationOne Liquid CDx results was performed using the multiple imputation method. Based on the 100 bootstrap samples with 50 times imputation, the estimated ORR of the FoundationOne Liquid CDx *NTRK*-positive population was 67.5% [52.4%, 87.1%].

There were 33 *NTRK1/2/3*-positive patients by the CTAs with partial or complete response to entrectinib, who also had an FoundationOne Liquid CDx result. Among them, only 54.5% (18/33) were positive by FoundationOne Liquid CDx (95% CI: 38.0, 70.2). There were 18 CTA-positive patients who did not respond to entrectinb, who also had an FoundationOne Liquid CDx result (25-18=7 and 26- 15=11). Among them, 38.9% (7/18) were positive by FoundationOne Liquid CDx (95% CI: 20.3, 61.4).

There were 25 patients positive for an *NTRK3* fusion in the entrectinib clinical studies. Among them, 68.0% (17/25) were negative for *NTRK3* fusions by FoundationOne Liquid CDx. Among the 17 patients who were negative for *NTRK3* fusions by FoundationOne Liquid CDx, 64.7% (11/17) had response to entrectinib. Furthermore, FoundationOne Liquid CDx detected one of seven different *NTRK3* fusions that were detected by the CTAs.

## 10.9 Clinical Bridging Study: Detection of BRAF V600E to Determine Eligibility for Treatment with encorafenib in combination with cetuximab

The clinical performance of FoundationOne Liquid CDx for the detection of *BRAF* V600E in plasma samples from patients with metastatic CRC for treatment with BRAFTOVI® (encorafenib) in combination with cetuximab was established through a clinical bridging study that assessed clinical efficacy of encorafenib and cetuximab in patients selected based on FoundationOne Liquid CDx results. Baseline plasma samples for patients enrolled in the BEACON (ARRAY-818-302) clinical trial were retrospectively tested by FoundationOne Liquid CDx in the bridging study. The study results demonstrate concordance between the CTA and the FoundationOne Liquid CDx assay, and establishes the clinical effectiveness of the FoundationOne Liquid CDx assay in identifying metastatic CRC patients with *BRAF* V600E for treatment with encorafenib in combination with cetuximab.

The BEACON trial was a randomized, open-label, multi-center, parallel group, three-arm Phase 3 study in patients with *BRAF* V600E in CRC whose disease had progressed after 1 or 2 prior regimens in the metastatic setting. The study compared the efficacy and safety of binimetinib + encorafenib + cetuximab (Triplet Arm), and encorafenib + cetuximab (Doublet Arm) to irinotecan/cetuximab or FOLFIRI/cetuximab (Control Arm).

The supplemental new drug application population included enrolled patients from the Control and Doublet arms from BEACON. The clinical trial tested the efficacy of therapy with these drugs by screening for and selecting metastatic CRC patients that harbor the *BRAF* V600E using the CTA which uses FFPE tissue DNA as the sample input. Overall survival (OS) and objective response rate (ORR) by RECIST v1.1 were the primary efficacy endpoints.

This study evaluated the clinical validity of FoundationOne Liquid CDx as a CDx to identify *BRAF* V600E positive patients from the BEACON clinical trial. FoundationOne Liquid CDx testing was performed on patients with available plasma samples from the BEACON clinical trial that tested positive for *BRAF* V600E by CTA+. Additionally, commercially procured *BRAF* V600E negative CRC patient tissue samples with matched plasma were tested.

The concordance between the CTA and FoundationOne Liquid CDx was evaluated by the PPA and NPA (**Table 64**). The prevalence-adjusted PPV and NPV were also calculated by adjusting for the prevalence of *BRAF* V600E among the intention-to-treat (ITT) population, with 10% and 15% as the estimated prevalence. The PPA, NPA, PPV, NPV, and their two-sided 95% CIs are provided in **Table** **65**.

**Table 64. Concordance for BRAF V600E between FoundationOne Liquid CDx and the CTA**

|  |  |  | **CTAs** |  |
| --- | --- | --- | --- | --- |
|  |  | **Detected** | **Not Detected** | **Total** |
| **F1LCDx** | **Detected** | 286 | 3 | 289 |
|  | **Not Detected** | 42 | 102 | 144 |
|  | **Unevaluable** | 74 | 16 | 90 |
|  | **Total** | 402 | 121 | 523 |
| **Agreement Statistics**  **Excluding CDx- Unevaluable Results** | | PPA: 87.2% (286/328)  95% CI^1^: (83.1%,  90.4%) | NPA: 97.1% (102/105) 95% CI^1^: (91.9%, 99.0%) |  |
| **Percent Unevaluable** | | 18.4% (74/402) | 13.2% (16/121) |  |

^1^Calculated with Wilson 2-sided 95% CI.

**Table 65. Concordance Analysis Results**

|  | **Prevalence** | **Numerator** | **Denominator** | **Point Estimate (%)** | **95% Two-Sided CI* (%)** |
| --- | --- | --- | --- | --- | --- |
| **PPA** | N/A | 286 | 328 | 87.20 | [83.14, 90.39] |
| **NPA** | N/A | 102 | 105 | 97.14 | [91.93, 99.02] |
|  | **Prevalence** | **Numerator** | **Denominator** | **Point Estimate (%)** | **95% Two-Sided CI* (%)** |
| **Adjusted PPV** | 10% | N/A | N/A | 77.23 | [59.41, 100.00] |
| **Adjusted NPV** | 10% | N/A | N/A | 98.56 | [98.17, 98.94] |
| **Adjusted PPV** | 15% | N/A | N/A | 84.34 | [69.92, 100.00] |
| **Adjusted NPV** | 15% | N/A | N/A | 97.73 | [97.12, 98.33] |

*CI was calculated using the Wilson-score method for PPA and NPA, while using the bootstrap method for the adjusted PPV and NPV.

The clinical validity of FoundationOne Liquid CDx was demonstrated by assessing clinical efficacy in the FoundationOne Liquid CDx *BRAF* V600E positive population based on the ORR difference between the Doublet Arm and Control Arm, as well as the log hazard ratio (log(HR)) between the 2 arms from the Cox regression model. The ORR is defined as the proportion of patients with objective response of either confirmed CR or PR based on RECIST V1.1. The ORR for the Doublet Arm and Control Arm as well as the ORR difference are reported in **Table 66** for the following subpopulations: CTA+, F1LCDx+|CTA+, F1LCDx-|CTA+, and FoundationOne Liquid CDx unevaluable|CTA+**. Table 66** also summarizes the median OS by the Kaplan-Meier method for each arm as well as the log(HR) with 95% two-sided CI for each of the aforementioned subpopulations.

**Table 66. Primary Efficacy in the Bridging Study Subpopulations**

|  | **CTA+** | **F1LCDx+ \| CTA+** | **F1LCDx- \| CTA+** | **F1LCDx unevaluable \| CTA+** |
| --- | --- | --- | --- | --- |
| **# Total** | 402 | 286 | 42 | 74 |
| **ORR for Doublet Arm** | 19.90% | 18.49% | 17.39% | 28.13% |
| **ORR for Control Arm** | 1.49% | 1.43% | 0.00% | 2.38% |
| **ORR Difference (95% two-sided CI)*** | 18.41%  [12.74%, 24.55%] | 17.06%  [10.51%, 24.22%] | 17.39% [-2.39%, 37.14%] | 25.74%  [9.73%, 43.10%] |
| **Median OS (months) for Doublet Arm** | 9.49 | 7.62 | NA§ | 18.89 |
| **Median OS (months) for Control Arm** | 5.88 | 5.38 | 12.16 | 7.16 |
| **log(HR) (95% two-sided**  **CI)** | -0.51  [-0.76, -0.26] | -0.47  [-0.75, -0.19] | -2.72  [-4.71, -0.74] | -0.44  [-1.23, 0.34] |

*CI was calculated using the Newcombe method.

^§^The estimated median OS is NA due to the small number of events in this group (3 events).

The clinical validity of FoundationOne Liquid CDx was demonstrated by estimating the ORR difference and log(HR) between the Doublet Arm and Control Arm. The estimated efficacy results for the FoundationOne Liquid CDx-positive (F1LCDx+) population are shown in **Table 67** below. Estimated efficacy results for the F1LCDx+ population, which were comparable to that in the CTA+ population are shown in **Table 66** above.

**Table 67. Estimated Efficacy for the F1LCDx+ Population**

|  | **Estimated F1LCDx+ Efficacy with 95% CI (log (HR))** | **Estimated F1LCDx+ Efficacy with 95% CI (ORR difference)** |
| --- | --- | --- |
|  | **prev = 10%** | |
| **c*=0%** | -0.36 [-0.61, -0.12] | 13.18 [6.86, 19.50] |
| **c=30%** | -0.40 [-0.63, -0.16] | 14.34 [8.48, 20.21] |
| **c=50%** | -0.42 [-0.65, -0.18] | 15.12 [9.48, 20.76] |
| **c=70%** | -0.44 [-0.67, -0.21] | 15.90 [10.42, 21.38] |
| **c=100%** | -0.47 [-0.70, -0.24] | 17.06 [11.67, 22.46] |
|  | **prev = 15%** | |
| **c*=0%** | -0.40 [-0.65, -0.15] | 14.39 [8.20, 20.58] |
| **c=30%** | -0.42 [-0.67, -0.17] | 15.19 [9.25, 21.14] |
| **c=50%** | -0.43 [-0.68, -0.19] | 15.73 [9.90, 21.55] |

|  | **Estimated F1LCDx+ Efficacy with 95% CI (log (HR))** | **Estimated F1LCDx+ Efficacy with 95% CI (ORR difference)** |
| --- | --- | --- |
| **c=70%** | -0.45 [-0.69, -0.21] | 16.26 [10.52, 22.01] |
| **c=100%** | -0.47 [-0.71, -0.23] | 17.06 [11.37, 22.76] |

*c is the ratio of efficacy between F1LCDx+|CTA- and F1LCDx+|CTA+ populations.

A sensitivity analysis was performed to assess the robustness of the concordance and efficacy results subject to the missing FoundationOne Liquid CDx test results. FoundationOne Liquid CDx *BRAF* V600E status were predicted for the FoundationOne Liquid CDx unevaluable patients (patients with missing or invalid FoundationOne Liquid CDx test results). The concordance analysis and the clinical efficacy were updated by accounting for the imputed data.

The PPA and prevalence adjusted PPV estimates were computed for each of the 50 imputed complete data sets and the summary statistics are shown in **Table 68**.

**Table 68. Summary Statistics of PPA and PPV on Imputed Complete Data**

|  | **Prev** | **Min** | **Q1** | **Median** | **Mean** | **Q3** | **Max** | **2.5%** | **97.5%** |
| --- | --- | --- | --- | --- | --- | --- | --- | --- | --- |
| **PPA (%)** | N/A | 84.29 | 85.04 | 85.54 | 85.46 | 85.79 | 86.78 | 84.54 | 86.53 |
| **PPV (%)** | 10% | 76.62 | 76.78 | 76.89 | 76.87 | 76.94 | 77.14 | 76.68 | 77.09 |
| **PPV (%)** | 15% | 83.89 | 84.01 | 84.08 | 84.07 | 84.12 | 84.28 | 83.93 | 84.24 |

In addition, the drug efficacy for the F1LCDx+|CTA+ population with the imputed complete data set is shown in **Table 69**. The estimated efficacy results for the F1LCDx+ population in the sensitivity analysis are shown in **Table 70**. The sensitivity analysis demonstrated the robustness of the concordance between CTA and FoundationOne Liquid CDx and drug efficacy estimated in the F1LCDx+ population by accounting for the missingness of FoundationOne Liquid CDx status. This study demonstrated the clinical validity of using FoundationOne Liquid CDx as a CDx device to select metastatic CRC patients with *BRAF* V600E for the treatment with encorafenib in combination with cetuximab.

**Table 69. Summary Statistics of Estimated log(HR) and ORR Difference for the F1LCDx+|CTA+ Population on Imputed Complete Data**

| **F1LCDx+\|CTA+** | **Min** | **Q1** | **Median** | **Mean** | **Q3** | **Max** | **2.5%** | **97.5%** |
| --- | --- | --- | --- | --- | --- | --- | --- | --- |
| log (HR) | -0.59 | -0.55 | -0.53 | -0.53 | -0.51 | -0.47 | -0.59 | -0.48 |
| ORR (%) Difference | 17.88 | 18.61 | 18.96 | 18.91 | 19.28 | 19.82 | 17.92 | 19.72 |

**Table 70. Estimated Efficacy for the F1LCDx+ Population in the Sensitivity Analysis**

|  | **Estimated F1LCDx+ Efficacy with 95% CI (log (HR))** | **Estimated F1LCDx+ Efficacy with 95% CI (ORR difference)** |
| --- | --- | --- |
|  | **prev = 10%** | |
| **c*=0%** | -0.41 [-0.66, -0.15] | 14.54 [8.27, 20.81] |
| **c=30%** | -0.44 [-0.71, -0.18] | 15.85 [9.81, 21.89] |
| **c=50%** | -0.47 [-0.74, -0.20] | 16.73 [10.71, 22.74] |
| **c=70%** | -0.49 [-0.77, -0.22] | 17.60 [11.50, 23.70] |
| **c=100%** | -0.53 [-0.82, -0.24] | 18.91 [12.49, 25.34] |
|  | **prev = 15%** | |
| **c*=0%** | -0.45 [-0.71, -0.18] | 15.90 [9.78, 22.02] |
| **c=30%** | -0.47 [-0.74, -0.20] | 16.81 [10.75, 22.86] |
| **c=50%** | -0.49 [-0.76, -0.21] | 17.41 [11.32, 23.50] |
| **c=70%** | -0.50 [-0.79, -0.22] | 18.01 [11.83, 24.19] |
| **c=100%** | -0.53 [-0.82, -0.24] | 18.91 [12.49, 25.34] |

*c is the ratio of efficacy between F1LCDx+|CTA- and F1LCDx+|CTA+ populations.

## 10.10 Clinical Bridging Study: Detection of BRAF V600E to Determine Eligibility for Treatment

### with encorafenib in combination with binimetinib

The clinical performance of FoundationOne Liquid CDx for the detection of *BRAF* V600E in plasma samples from patients with NSCLC for treatment with BRAFTOVI® (encorafenib) in combination with MEKTOVI® (binimetinib) was established through a clinical bridging study. All available baseline plasma samples for patients enrolled in the PHAROS (ARRAY-818-202) clinical trial were tested by FoundationOne Liquid CDx in the bridging study. The study results demonstrate concordance between the CTAs and the FoundationOne Liquid CDx assay and establishes the clinical effectiveness of the FoundationOne Liquid CDx assay in selecting *BRAF* V600E positive patients with NSCLC for the treatment with encorafenib in combination with binimetinib.

The PHAROS trial is an open-label, multicenter, single-arm study in patients with *BRAF* V600E-positive metastatic NSCLC. Eligible patients were either treatment-naïve or had received treatment with chemotherapy and/or immunotherapy (previously treated).

The efficacy population for the supplemental new drug applications included 59 treatment-naïve patients, and 39 previously treated patients with the locally confirmed *BRAF* V600E who were enrolled into the PHAROS clinical trial. The clinical trial evaluated the efficacy of encorafenib + binimetinib by screening and enrolling NSCLC patients whose tumors harbored a *BRAF* V600E substitution using either a PCR or NGS-based local laboratory assay and using either tumor tissue or blood.

This study evaluated the clinical validity of FoundationOne Liquid CDx as a CDx to identify *BRAF* V600E positive patients from the PHAROS clinical trial. FoundationOne Liquid CDx testing was performed on patients with available plasma samples from the PHAROS clinical trial that tested positive for *BRAF* V600E by CTA+. Additionally, commercially procured *BRAF* V600E negative NSCLC patient tissue samples with matched plasma were tested.

**Table 71. FoundationOne Liquid CDx Bridging Study Sample Accountability**

| **Source of Samples** | **Total # of Patients** | **# of Failed or**  **Unavailable for**  **F1LCDx Testing** | **Total # of F1LCDx Evaluable** |
| --- | --- | --- | --- |
| **CTA+*** | 98 | 17 | 81 |
| **CTA- Procured Samples Tested by Cobas PCR assay** | 28 | 9 | 19 |
| **CTA- Procured Samples Tested by UW OncoPlex** | 42 | 12 | 30 |
| **CTA- from Foundation Medicine Archived Samples** | 50 | 0 | 50 |
| **Total** | 218 (100%) | 38 (17.43%) | 180 (82.56%) |

^*^Six (6) patients from the clinical trial were enrolled by FoundationOne CDx, and were treated as CTA+.

The PPA was 59.26% (48/81) with two-sided 95% confidence interval (CI) (48.38%, 69.30%) and the NPA was 100% (99/99) with two-sided 95% CI (96.26%, 100%) after excluding FoundationOne Liquid CDx-unevaluable results when considering both patients that were treatment naïve and previously treated (**Table 72**). Since patients were enrolled and initially tested by local CTAs, the prevalence- adjusted PPVs and NPVs were calculated using the PPA and NPA, after adjusting for the prevalence of *BRAF* V600E among the ITT population. In the analysis with 1% prevalence, FoundationOne Liquid CDx demonstrated an adjusted PPV of 100% with two-sided 95% CI (92.59%, 100%) and NPV of 99.59% with two-sided 95% CI (99.48%, 99.69%). PPAs of 62% (31/50) and 55% (17/31) were observed for patients that were from treatment-naïve and previously treated patient cohorts, respectively, indicating comparable detection of the *BRAF* V600E substitution in plasma by FoundationOne Liquid CDx among the two patient populations.

**Table 72. Concordance for BRAF V600E between FoundationOne Liquid CDx and the CTAs**

|  |  |  | **CTAs** |  |
| --- | --- | --- | --- | --- |
|  |  | **Detected** | **Not Detected** | **Total** |
| **F1LCDx** | **Detected** | 48 | 0 | 48 |
|  | **Not Detected** | 33 | 99 | 132 |
|  | **Unevaluable** | 17 | 21 | 35 |
|  | **Total** | 98 | 120 | 218 |
| **Agreement Statistics**  **Excluding CDx- Unevaluable Results** | | PPA: 59.26% (48/81)  95% CI^1^: (48.38%,  69.30%) | NPA: 100% (99/99)  95% CI^1^: (96.26%,  100%) |  |
| **Percent Unevaluable** | | 17.3% (17/98) | 17.5% (21/120) |  |

^1^Calculated with Wilson 2-sided 95% CI.

The clinical validity of FoundationOne Liquid CDx was demonstrated by assessing clinical efficacy in the FoundationOne Liquid CDx *BRAF* V600E positive population based on ORR as the primary efficacy endpoint, which is defined as the proportion of patients with best overall response of confirmed CR or PR as determined by independent review committee (IRC) per RECIST v1.1. To evaluate the secondary efficacy endpoint, duration of response (DOR), the median of DOR was calculated by the Kaplan-Meier method along with its two-sided 95% CI for all the 98 CTA+ patients. The results are reported in **Table 73** and **Table 74.** The estimated ORR and the corresponding 95% confidence intervals for the F1LCDx+ population in the treatment-naïve cohort and previously treated cohort were 74.19% (58.79%, 89.60%) and 35.29% (12.58%, 58.01%), respectively, which were comparable with the observed ORR for CTA biomarker positive population.

**Table 73. Primary Efficacy in the Bridging Study Subpopulations (treatment-naïve Cohort)**

|  | **CTA+** | **F1LCDx+ \| CTA+** | **F1LCDx- \| CTA+** | **F1LCDx unevaluable \|**  **CTA+** |
| --- | --- | --- | --- | --- |
| **No. of patients** | 59 | 31 | 19 | 9 |
| **No. of events (CR or PR)** | 44 | 23 | 13 | 8 |
| **ORR (%)** | 74.58 | 74.19 | 68.42 | 88.89 |
| **Two-sided 95% CI for**  **ORR**£ | [62.20, 83.94] | [56.75, 86.30] | [46.01, 84.64] | NA* |
| **Median DOR (Two- sided 95% CI)** | N/A** [23.1, N/A^§^] | 23.1  [12.0, N/A^§^] | N/A** [N/A^§^, N/A^§^] | 16.13  NA* |
| **# Patients with DOR**  **≥6 months (%)** | 33 (75) | 15 (65.2) | 12 (92.3) | 6 (75) |
| **# Patients with DOR**  **≥12 months (%)** | 26 (59.1) | 11 (47.8) | 11 (84.6) | 4 (50) |

*CI was not calculated since the sample size is less than 10.

^£^CI was calculated using the Wilson-Score method. Please refer to the drug label for the CI calculated using the exact method.

^**^Median DOR is unavailable since the response rate did not fall below 50% in the Kaplan-Meier estimate.

^§^NAs in the lower (upper) 95% CI of median DOR are due to the lower (upper) 95% CI of the response rate that did not fall below 50%.

**Table 74. Primary Efficacy in the Bridging Study Subpopulations (previously treated Cohort)**

|  | **CTA+** | **F1LCDx+ \| CTA+** | **F1LCDx- \| CTA+** | **F1LCDx unevaluable \|**  **CTA+** |
| --- | --- | --- | --- | --- |
| **No. of patients** | 39 | 17 | 14 | 8 |
| **No. of events (CR or PR)** | 18 | 6 | 6 | 6 |
|  | **CTA+** | **F1LCDx+ \| CTA+** | **F1LCDx- \| CTA+** | **F1LCDx unevaluable \|**  **CTA+** |
| **ORR (%)** | 46.15 | 35.29 | 42.86 | 75.00 |
| **Two-sided 95% CI for**  **ORR**£ | [31.57, 61.42] | [17.31, 58.70] | [21.38, 67.41] | NA* |
| **Median DOR (Two- sided 95% CI)** | 16.72  [11.93, N/A^§^] | 16.72  N/A* | 11.93  N/A* | N/A**  N/A* |
| **# Patients with DOR**  **≥6 months (%)** | 12 (66.7) | 5 (83.3) | 4 (66.7) | 3 (50.0) |
| **# Patients with DOR**  **≥12 months (%)** | 6 (33.3) | 2 (33.3) | 2 (33.3) | 2 (33.3) |

^*^CI was not calculated since the sample size is less than 10.

^£^CI was calculated using the Wilson-Score method. Please refer to the drug label for the CI calculated using the Exact method.

^**^Median DOR is unavailable since the response rate did not fall below 50% in the Kaplan-Meier estimate.

^§^NAs in the upper 95% CI of median DOR are due to the upper 95% CI of the response rate that did not fall below 50%.

To assess the robustness of the data subject to missing FoundationOne Liquid CDx test results, a sensitivity analysis was performed, and multiple imputations were used to impute the FoundationOne Liquid CDx *BRAF* V600E status in the FoundationOne Liquid CDx unevaluable population. The concordance analysis and the clinical efficacy for FoundationOne Liquid CDx *BRAF* V600E positive patients were updated by accounting for the imputed data. Multiple imputations were conducted in the original dataset and a total of 200 imputation data sets were generated. The sensitivity analysis demonstrated the robustness of the clinical bridging results. In the sensitivity analysis, the mean PPA between the CTAs and the FoundationOne Liquid CDx was 61.2% with two-sided 95% empirical confidence interval [57.73%, 63.92%], which was comparable to the observed data 59.26% with two- sided 95% confidence interval [48.38%, 69.30%]. The adjusted PPV on the imputed complete data was 100% across all prevalence values.

The primary efficacy outcome (ORR) was also estimated for each of the imputed complete datasets.The mean ORR for the F1LCDx+ was estimated to be 76.32% with two-sided 95% confidence interval [62.40%, 90.25%] for treatment naïve patients , and 43.28% with two-sided 95% confidence interval [22.31%, 64.2%] for previously treated patients, respectively. The efficacy results for F1LCDx+ population in the sensitivity analysis are comparable to that of the CTA+ population, which demonstrates the clinical validity of FoundationOne Liquid CDx in identifying *BRAF* V600E positive patients with NSCLC for treatment with encorafenib in combination with binimetinib. The sensitivity analysis also demonstrated the robustness of the concordance and efficacy results to the missing FoundationOne Liquid CDx results.

## 10.11 Clinical Bridging Study: Detection of BRCA1 and BRCA2 Alterations to Determine Eligibility for Treatment with niraparib + abiraterone acetate

The clinical performance of FoundationOne Liquid CDx in detecting *BRCA1*, *BRCA2* alterations (*BRCA1/2*) in patients with prostate cancer (PC) who may benefit from treatment with niraparib in combination with abiraterone acetate and prednisone (AAP) (i.e., AKEEGA plus prednisone) was established via a clinical bridging study. All available baseline plasma samples for patients enrolled in the MAGNITUDE clinical trial were tested by FoundationOne Liquid CDx.

The efficacy of AKEEGA (niraparib and abiraterone acetate) was investigated in Cohort 1 of MAGNITUDE (NCT03748641). The MAGNITUDE study was a Phase 3, randomized, double-blinded, placebo-controlled, multicenter study of AKEEGA versus placebo for treatment of patients with metastatic PC. All patients received prednisone. Participants in the MAGNITUDE study were assigned to cohorts based on homologous recombination repair (HRR) alteration status as determined by tissue (majority F1CDx) and/or plasma CTAs. Cohort 1 includes patients with an HRR gene alteration as detected (i.e., HRR+) by at least one assay (tissue or plasma), and Cohort 2 includes patients where no HRR gene alteration was detected (i.e., HRR-) in both assays or HRR- by one assay and unevaluable by the other.

The clinical validity of FoundationOne Liquid CDx was demonstrated by assessing clinical efficacy in the FoundationOne Liquid CDx *BRCA1/2* positive population based on radiographic progression free survival (rPFS) determined by blinded independent central radiology (BICR) review.

Sample accountability

The clinical bridging analysis population included 473 patients from the MAGNITUDE clinical trial, including all patients from Cohort 1 (n=423) and 50 patients randomly selected from Cohort 2. Both Cohort 1 and Cohort 2 patients were used to support concordance analysis while only Cohort 1 samples were used for the efficacy analysis. Among the 473 samples, 443 were tested by FoundationOne Liquid CDx, 396 yielded valid FoundationOne Liquid CDx testing results and 47 failed lab processing (LC, HC, and sequencing) or post-sequencing QC metrics. The overall success rate is 89.39% (396/443, 95% CI: [86.18%, 91.93%]).

Among the 473 patients enrolled into Cohort 1 and Cohort 2, 225 (47.57%) were *BRCA1/2+* by the enrolling CTA (i.e., CTA+) and 183 passed F1LCDx testing; 248 (52.43%) were *BRCA1/2-* by the enrolling CTA (i.e., CTA-) and 213 passed F1LCDx testing.

Table 75. Sample Accountability for FoundationOne Liquid CDx vs Enrolling CTA

| **Enrolling CTA Status** | **F1LCDx Testing Status** | **COHORT** | **N (%)** |
| --- | --- | --- | --- |
| CTA+ | Available for F1LCDx testing | COHORT 1 | 202 (42.71%) |
|  | Passed F1LCDx Testing |  | 183 (38.69%) |
|  | Failed F1LCDx Testing |  | 19 (4.02%) |
|  | Not Tested by F1LCDx, Not Enough Plasma |  | 8 (1.69%) |
|  | Not Tested by F1LCDx, plasma CTA Unevaluable |  | 15 (3.17%) |
|  | Total | | 225 (47.57%) |
| CTA- | Available for F1LCDx testing | COHORT 1 | 191 (40.38%) |
|  |  | COHORT 2 | 50 (10.57%) |
|  | Passed F1LCDx Testing | COHORT 1 | 169 (35.73%) |
|  |  | COHORT 2 | 44 (9.30%) |
|  | Failed F1LCDx Testing | COHORT 1 | 22 (4.65%) |
|  |  | COHORT 2 | 6 (1.27%) |
|  | Not Tested by F1LCDx, Not Enough Plasma | COHORT 1 | 3 (0.63%) |
|  | Not Tested by F1LCDx, plasma CTA Unevaluable | COHORT 1 | 4 (0.85%) |
|  | Total | | 248 (52.43%) |
| Total | | | 473 (100.00%) |

The concordance analysis between FoundationOne Liquid CDx and enrolling CTA was performed using samples from both Cohort 1 and Cohort 2. The PPA and NPA were calculated and presented in **Table 76**. Adjusted PPV and NPV were calculated using a prevalence of 9.4% (280/2982), which is the proportion of the *BRCA1/2+* population identified by enrolling CTA from the screening population. The

PPV and NPV were estimated as

- Adjusted PPV: 64.09% (95% CI: [51.90%, 80.64%])
- Adjusted NPV: 97.13% (95% CI: [96.46%, 97.78%])

Table 76. Concordance for BRCA1, BRCA2 alterations between FoundationOne Liquid CDx and the Enrolling CTA

|  |  | **CTA** |  |  |
| --- | --- | --- | --- | --- |
|  |  | **Detected** | **Not Detected** | **Total** |
| **F1LCDx** | **Detected** | 133 | 9 | 142 |
|  | **Not Detected** | 50 | 204 | 254 |
|  | **Unevaluable** | 42 | 35 | 77 |
|  | | **CTA** |  |  |
|  |  | **Detected** | **Not Detected** | **Total** |
|  | **Total** | 225 | 248 | 473 |
| **Agreement Statistics**  **Excluding CDx-Unevaluable**  **Results** | | PPA: 72.68% (133/183)  95% CI^1^: (65.80%, 78.62%) | NPA: 95.77% (204/213)  95% CI^1^: (92.17%, 97.76%) |  |
| **Percent Unevaluable** | | 18.67% (42/225) | 14.11% (35/248) |  |

^1^Calculated with Wilson 2-sided 95% CI.

A summary of the median rPFS and hazard ratio (HR) was provided in **Table 77** for each subgroup within Cohort 1. For the F1LCDx+|enrolling CTA+ subgroup the estimated HR using a stratified Cox regression was 0.49 (95% CI: [0.29, 0.81]) , which suggested a 51% reduction in the risk of radiographic progression when using AKEEGA compared with placebo, comparable with the HR of 0.53 (95% CI: [0.36, 0.79]) for the CTA+ population

Table 77. Estimation of median rPFS and HR

| **Subgroup** | **No. of Patients** | | **No. of Events** | | **Median rPFS (months) [95% CI**^1^**]** | | **HR**  **[95% CI]** |
| --- | --- | --- | --- | --- | --- | --- | --- |
|  | **AKEEGA** | **Placebo** | **AKEEGA** | **Placebo** | **AKEEGA** | **Placebo** |  |
| CTA+ | 113 | 112 | 45 | 64 | 16.56  [14.42, NA] | 10.87  [8.34, 13.93] | 0.53  [0.36, 0.79] |
| F1LCDx+\|CTA+ | 63 | 70 | 25 | 42 | 18.43  [13.83, NA] | 11.04  [8.31, 16.39] | 0.49  [0.29, 0.81] |
| F1LCDx-\|CTA+ | 30 | 20 | 12 | 11 | 16.66  [14.98, NA] | 8.44  [8.28, NA] | 0.49  [0.19, 1.26] |
| F1LCDx- evaluable\|CTA+ | 93 | 90 | 37 | 53 | 16.66  [14.98, NA] | 9.03  [8.31, 13.93] | 0.53  [0.34, 0.81] |
| F1LCDx- unevaluable\|CTA+ | 20 | 22 | 8 | 11 | 13.86  [11.10, NA] | 10.91  [7.59, NA] | 0.52  [0.18, 1.48] |

^1^The 95% CI upper/lower bound was NA when the upper/lower bound of the confidence band for rPFS probability did not drop below 50% and thus could not be estimated.

Among the 50 F1LCDx-|CTA+ patients, 23 were plasma CTA+ only (13 F1CDx- and 10 F1CDx unevaluable), 23 were F1CDx+ only (all plasma CTA-), 2 were F1CDx+ and plasma CTA+, and 2 were CTA+ by a local tissue-based test (all F1CDx unevaluable and plasma CTA-). The 25 F1LCDx-|F1CDx+ included 15 *BRCA1/2* homozygous deletions (HD), 2 rearrangements (RE), and 8 short variants (SVs). A total of 33 patients enrolled and treated in MAGNITUDE had a homozygous deletion in *BRCA1/2* as detected by F1CDx; 7 of them were F1LCDx unevaluable, 21 were F1LCDx HD-, and 5 were F1LCDx HD+. The HR estimated by the stratified Cox regression was 0.233 (95% CI: [0.0044, 1.23]) in the F1LCDx-|F1CDx+ HD subpopulation. These results indicate that F1LCDx may miss a subset of patients with prostate cancer harboring *BRCA1/2* homozygous deletions who may derive benefit from AKEEGA since the data demonstrated that the FoundationOne Liquid CDx test did not detect approximately 81% of prostate cancer patients with *BRCA/2* homozygous deletions who responded to AKEEGA therapy.

Since insufficient samples were observed in the F1LCDx+|CTA- subgroup and the prevalence-adjusted PPV was <100%, the efficacy of the F1LCDx+|CTA- population was estimated using the tipping point method where *c* is a constant that varies from 0% (worst-case scenario) to 100% (best-case scenario) to assess if the estimates of the efficacy for the F1LCDx positive population in cohort 1 are robust under different values of *c*. The HR estimated by the stratified Cox regression ranged between 0.49 (95% CI: [0.33, 0.71]) and 0.63 (95% CI: [0.42, 0.93]) as the scaling factor *c* went from 100% to 0% which demonstrated statistically significant clinical efficacy among the FoundationOne Liquid CDx *BRCA1/2*+ population (**Table 78**).

Table 78. Estimations of HR for the F1LCDx BRCA1/2+ population

| ***c***1 | **HR between treatment and control arms** | **95% CI for HR between treatment and control arms** |
| --- | --- | --- |
| 0% | 0.63 | [0.42, 0.93] |
| 30% | 0.58 | [0.40, 0.86] |
| 50% | 0.55 | [0.38, 0.81] |
| 70% | 0.53 | [0.36, 0.77] |
| 100% | 0.49 | [0.33, 0.71] |

^1^ *c* is a constant ranging from 0% to 100% and is used in the tipping point method for efficacy estimation of the F1LCDx+|CTA- population.

A sensitivity analysis was performed to assess the robustness of concordance and efficacy results subject to missing F1LCDx results. Multiple imputation methodology was used to impute the FoundationOne Liquid CDx *BRCA1/2* status for the F1LCDx-unevaluable but CTA-evaluable patients. The concordance and efficacy were re-estimated using the complete dataset including the imputed status. The median of the PPAs calculated using the complete datasets was 73.78% (95% CI: [72.44%, 76.00%]); the median of the prevalence-adjusted PPV was 63.45% (95% CI: [59.55%, 68.45%]); the HR estimated using the complete datasets ranged between 0.49 (95% CI: [0.30, 0.81]) and 0.63 (95% CI: [0.44, 0.91]). A separate sensitivity analysis using the weighted efficacy that considered the efficacy in Cohort 2 as missing was conducted. Following this approach, the HR estimated based on the stratified Cox regression using the complete dataset ranged from 0.49 (95% CI: [0.34, 0.71]) and 0.69 (95% CI: [0.49, 0.96]). Comparable results were seen with those from the observed dataset, which demonstrated that the concordance and efficacy results were robust after accounting for the missing FoundationOne Liquid CDx results.

In summary, this study demonstrated the clinical validity of using FoundationOne Liquid CDx as a CDx device to select patients with PC with *BRCA1*, *BRCA2* alterations for treatment with AKEEGA.

## 10.12 Clinical Bridging Study: Detection of BRCA1 and BRCA2 Alterations to Determine Eligibility for Treatment with LYNPARZA® in combination with abiraterone

The clinical performance of FoundationOne Liquid CDx in detecting *BRCA1* and *BRCA2* alterations (*BRCA1/2*) in patients with metastatic castration-resistant prostate cancer who may benefit from LYNPARZA (olaparib) in combination with abiraterone was established with clinical data generated from FoundationOne Liquid CDx in the clinical study D081SC00001 (hereafter referred to as PROpel).

The PROpel trial was a randomized, double-blind, placebo-controlled, multicenter study of olaparib plus abiraterone relative to placebo plus abiraterone as first line therapy in men with mCRPC. Patients were randomized 1:1 to receive either olaparib plus abiraterone or placebo plus abiraterone. All patients received either prednisone or prednisolone 5 mg twice daily, and a GnRH analog or prior bilateral orchiectomy. Enrollment into the PROpel trial followed an all-comer approach. Genomic testing was conducted after patient randomization and before the primary analysis of the study.

The major efficacy outcome measure of the PROpel study was investigator-assessed radiographic progression free survival (rPFS). Overall survival (OS) was an additional efficacy outcome measure. *BRCA1/2* mutation (*BRCAm*) status was assessed after randomization and before the primary analyses by both NGS-based tumor tissue (FoundationOne CDx) and ctDNA (FoundationOne Liquid CDx) tests.

A total of 1103 patients were enrolled in PROpel. Of these, 796 patients were randomized in PROpel while 307 patients were not randomized. Of the 796 patients randomized in PROpel, tissue samples were available for 782 patients (98.2%; 782/796) to enable FoundationOne CDx testing, and ctDNA samples were available for 794 patients (99.7%; 794/796) to enable FoundationOne Liquid CDx testing.

The concordance between FoundationOne CDx (as reference) and FoundationOne Liquid CDx was evaluated by calculation of the positive percent agreement (PPA), negative percent agreement (NPA), positive predictive value (PPV) and negative predictive value (NPV) in the 491 patients randomized in PROpel with valid results for both FoundationOne CDx and FoundationOne Liquid CDx tests (**Table 79**).

**Table 79. Concordance between FoundationOne CDx and FoundationOne Liquid CDx BRCAm status in PROpel**

|  |  | **FoundationOne CDx tissue test** | |  |
| --- | --- | --- | --- | --- |
|  |  | ***BRCAm*** | **Non-*BRCAm*** | **Total** |
| **FoudationOne**  **Liquid CDx**    **ctDNA test** | ***BRCAm*^a^** | 34 | 18 | 52 |
|  | **Non-*BRCAm*^b^** | 12 | 427 | 439 |
|  | **Total** | 46 | 445 | 491 |
|  |  |  | |  |
|  | PPA % (95% CI) |  | 73.9 (59.7, 84.4) |  |
|  | NPA % (95% CI) |  | 96.0 (93.7, 97.4) |  |
|  | PPV % (95% CI) |  | 65.4 (51.8, 76.8) |  |
|  | NPV % (95% CI) |  | 97.3 (95.3, 98.4) |  |

Note: The reference is the tumor tissue test (FoundationOne CDx).

^a^ Defined as any deleterious or suspected deleterious *BRCA1/2* gene mutation detected. ^b^ Defined as no deleterious or suspected deleterious *BRCA1/2* gene mutation detected. Test failed/samples are not included in concordance analysis. PPA = Positive Percentage Agreement, NPA = Negative Percentage Agreement, PPV = Positive Predictive Value, NPV = Negative Predicted Value. The 95% confidence interval (CI) for PPA, NPA, PPV and NPV is calculated using the Wilson method.

The 12 F1LCDx-|F1CDx+ patients included 7 *BRCA1/2* homozygous deletions (HD),3 short variants (SVs), and 2 rearrangements. A total of 10 patients enrolled and treated in PROpel had a homozygous deletion in *BRCA1/2* as detected by FoundationOne CDx, 3 of them were HD+ and 7 were HD- by FoundationOne Liquid CDx testing. All (7/7) of the HD F1CDx+|F1LCDx- had tumor fraction (TF) below the *BRCA2* HD LoD of the FoundationOne Liquid CDx assay. These results indicate that FoundationOne Liquid CDx may miss a subset of patients with prostate cancer harboring *BRCA1/2* homozygous deletions.

The clinical validity of FoundationOne Liquid CDx was demonstrated by assessing clinical efficacy in the *BRCA1/2* positive population based on rPFS and OS. Of the 796 randomized patients in PROpel,

85 had *BRCA*m determined by either a positive ctDNA test or a tumor tissue test. The clinical efficacy was evaluated in terms of rPFS by investigator assessment and OS. The HR was calculated using a Cox proportional hazards model.

A statistically significant improvement in rPFS for Lynparza/abiraterone compared to placebo/abiraterone was observed in the intention to treat (ITT) population. In an exploratory analysis in the subgroup of 711 patients without an identified *BRCA*m, the rPFS hazard ratio was 0.77 (95% CI: 0.63, 0.96) and the OS hazard ratio was 0.92 (95% CI: 0.74, 1.14), indicating that the improvement in the ITT population was primarily attributed to the results seen in the subgroup of patients with *BRCA*m.

In the analysis of *BRCA*m patients as determined by FoundationOne Liquid CDx, the rPFS hazard ratio was 0.18 (95% CI [0.08, 0.36] and the OS hazard ratio was 0.31 (95% CI [0.15, 0.63].The magnitude of treatment effect was also numerically greater in comparison to the non-*BRCAm* subgroup (rPFS HR=0.78; 95% CI [0.63, 0.97] and OS HR=0.89; 95% CI [0.72, 1.10]).

**Table 80** summarizes the efficay results in the 85 patients in PROpel with *BRCA*m as determined by either a positive FoundationOne Liquid CDx test and/or a positive FoundationOne CDx test (the total *BRCA*m population) and in the 69 patients with *BRCAm* as determined by FoundationOne Liquid CDx.

**Table 80. Efficacy analyses in PROpel**

|  | **Total *BRCAm* population (n=85)** | | **F1LCDx+ *BRCAm* population (n=69)** | |
| --- | --- | --- | --- | --- |
|  | **Lynparza/abiraterone**  N = 47 | **Placebo/abiraterone** | **Lynparza/abiraterone** | **Placebo/abiraterone** |
|  |  | N = 38 | N = 39 | N = 30 |
|  | **Radiological Progression-Free Survival (rPFS)^a^** | | | |
| Events, n (%) | 14 (30) | 28 (74) | 12 (31) | 25 (83) |
| Median (95% CI), months | NR (NR, NR) | 8 (6, 15) | NR (NR, NR) | 8 (5, 11) |
| Hazard ratio (95% CI)^b^ | 0.24 (0.12, 0.45) | | 0.18 (0.08, 0.36) | |
|  | **Overall Survival (OS)** | | | |
| Events, n (%) | 13 (28) | 25 (66) | 12 (31) | 21 (70) |
| Median (95% CI), months | NR (NR, NR) | 23 (18, 34) | NR (NR, NR) | 23 (16, 28) |
| Hazard ratio (95% CI)^b^ | 0.30 (0.15, 0.59) | | 0.31 (0.15, 0.63) | |

NR: Not reached ^a^Investigator-assessed

^b^Calculated using an unstratified univariable Cox proportional hazards model

A sensitivity analysis was performed to assess the robustness of the clinical efficacy estimated after accounting for the F1LCDx-unevaluable (failed FoundationOne Liquid CDx testing or not tested by FoundationOne Liquid CDx) results. Following imputation, the HR estimated for rPFS based on the Cox proportional hazards model using the complete dataset was 0.19 (95% CI: [0.09, 0.39]) and the HR estimated for OS based on the Cox proportional hazards model following imputation was 0.32 (95% CI: [0.16, 0.65]) in the *BRCA*m subgroup of patients as determined by FoundationOne Liquid CDx testing. Comparable results were seen with those from the observed dataset, which demonstrated the efficacy results were robust after accounting for the missing FoundationOne Liquid CDx results.

In summary, the data from this study demonstrated the clinical validity of using the FoundationOne Liquid CDx assay in identifying patients with metastatic castration-resistant prostate cancer with *BRCA1/2* alterations for the treatment with LYNPARZA (olaparib) in combination with abiraterone.

## 10.13 FoundationOne Liquid CDx Clinical Efficacy Study: Detection of PIK3CA mutations to determine eligibility with ITOVEBI™ (inavolisib) in combination with palbociclib and fulvestrant

The clinical validity of FoundationOne Liquid CDx assay as a companion diagnostic device for identifying *PIK3CA* mutations in locally advanced or metastatic breast cancer patients who may benefit from treatment with inavolisib in combination with palbociclib and fulvestrant was established using clinical data from the INAVO120 clinical trial.

INAVO120 (WO41554/NCT04191499) is a randomized, double-blind, placebo-controlled trial evaluating the efficacy of the triplet combination of inavolisib plus palbociclib and fulvestrant versus placebo plus palbociclib and fulvestrant in patients with PIK3CA-mutated, Hormone Receptor-Positive (HR+), HER2-negative (HER2-) locally advanced or metastatic breast cancer, who have not received prior systemic therapy for locally advanced or metastatic disease and whose disease progressed during or within 12 months of completing adjuvant endocrine therapy. PIK3CA mutation (PIK3CAm) status was prospectively determined in a central laboratory using the FoundationOne Liquid CDx assay on plasma-derived ctDNA or in local laboratories using various validated PCR or NGS assays on tumor tissue or plasma. All patients were required to provide both a freshly collected pre-treatment blood sample and a tumor tissue sample for central evaluation and determination of PIK3CAm status.

The major efficacy outcome measure was investigator (INV)-assessed progression-free survival (PFS) per Response Evaluation Criteria in Solid Tumors (RECIST) version 1.1. Additional efficacy outcome measures included overall survival (OS), INV-assessed objective response rate (ORR), and INVassessed duration of response (DOR).

A total of 325 patients were included in the NDA efficacy population for inavolisib or placebo plus palbociclib and fulvestrant. Of the 325 patients from the INAVO120 NDA population (after excluding 43 patients from China who were not available for F1LCDx testing), 1.4% (4/282) of patients had unevaluable F1LCDx test results and 98.6% (278/282) of patients had evaluable F1LCDx test results, including 95.4% (269/282) that were positive for one or more study-eligible *PIK3CA* mutation(s) by F1LCDx (i.e., F1LCDx-positive) and 3.2% (9/282) with no *PIK3CA* mutation detected (i.e., F1LCDxnegative). One additional F1LCDx-positive patient was excluded from the efficacy analyses due to not ultimately receiving their planned treatment with inavolisib in combination with palbociclib and fulvestrant.

In the efficacy analysis for the NDA population, the median PFS (months) with 95% 2-sided CI for the inavolisib cohort was 15.0 [11.3, 20.5] months, and the median PFS for the placebo cohort was 7.3 [5.6, 9.3] months. The PFS hazard ratio (HR) for inavolisib in combination with palbociclib and fulvestrant vs. placebo in combination with palbociclib and fulvestrant and the associated 2-sided 95% CI was estimated as 0.43 [0.32, 0.59].

The clinical validity of F1LCDx was demonstrated by assessing clinical efficacy in the F1LCDx PIK3CA mutation-positive population based on progression free survival (PFS) as the primary efficacy endpoint. The efficacy analysis dataset consists of PIK3CA mutation(s) positive, HR+, HER2- locally advanced or metastatic breast cancer patients from the NDA efficacy population, enrolled by a local assay or by the central F1LCDx assay, whose tumors (cfDNA) were F1LCDx-positive.

For each treatment arm, Kaplan-Meier curves were used to estimate the time-to-event distributions. The 50^th^ percentile of Kaplan-Meier estimates were used to estimate the median duration of PFS and are reported with a 2-sided 95% CI in **Table 81**. The HR was calculated using a Cox proportional hazards model.

**Table 81. Summary of PFS for Each Treatment Group for F1LCDx-positive Population***

*Treatment groups were defined by actual received treatment indicator (TRT01A) for the clinical validation study.

|  | **Total PIK3CAm population (n=325)** | | **F1LCDx+ PIK3CAm population**  **(n=268)** | |
| --- | --- | --- | --- | --- |
| **Efficacy Endpoint** | **ITOVEBI +**  **Palbociclib + Fulvestrant N=161** | **Placebo +**  **Palbociclib + Fulvestrant N=164** | **ITOVEBI +**  **Palbociclib + Fulvestrant N=137** | **Placebo +**  **Palbociclib + Fulvestrant N=131** |
| **Progression-Free Survival^a,b^** | | |  |  |
| Patients with event, n (%) | 82 (51) | 113 (69) | 68 | 92 |
| Median, months (95%  CI) | 15.0 (11.3, 20.5) | 7.3 (5.6, 9.3) | 16.6 (13.4, 24.2) | 7.3 (5.8, 10.1) |
| Hazard ratio (95% CI) | 0.43 (0.32, 0.59) | | 0.42 (0.30, 0.59) | |

CI = confidence interval. ^a^ Per RECIST version 1.1. ^b^ Based on investigator assessment.

Compared to the placebo cohort, in which the median PFS was 7.3 [5.8, 10.1] months, the median PFS for the inavolisib treatment cohort was 16.6 [13.4, 24.2] months. The PFS hazard ratio (HR) for inavolisib in combination with palbociclib and fulvestrant vs. placebo in combination with palbociclib and fulvestrant and the associated 2-sided 95% CI was estimated as 0.42 [0.30, 0.59], which were comparable to that in the total PIK3CA positive population.

A sensitivity analysis was performed to assess the robustness of the clinical efficacy estimated after accounting for the F1LCDx-unevaluable (failed FoundationOne Liquid CDx testing or not tested by FoundationOne Liquid CDx) results. Following imputation, the estimated HR for PFS based on the Cox proportional hazards model using the complete dataset was 0.44 (95%CI: [0.43, 0.44]) in the PIK3CA+ patients as determined by FoundationOne Liquid CDx testing. Comparable results were seen with those from the observed dataset, which demonstrated the efficacy results were robust after accounting for the missing FoundationOne Liquid CDx results.

## 10.14 Clinical Bridging Study: Detection of MET single nucleotide variants (SNVs) and indels that

### lead to MET exon 14 skipping to Determine Eligibility for Treatment with tepotinib

The clinical performance of F1LCDx in detecting SNVs and indels that lead to *MET* exon 14 skipping alterations in patients with advanced or metastatic NSCLC who may benefit from treatment with tepotinib was established through a clinical bridging study with clinical data for patients from the VISION clinical study (NCT02864992) and biomarker data from F1LCDx results.

The VISION clinical trial is a single-arm, open-label, multicenter, non-randomized, multicohort study of *MET* inhibitor, TEPMETKO (tepotinib) in adult patients with advanced or metastatic NSCLC harboring *MET* exon 14 skipping alterations. Identification of *MET*ex14 skipping alterations was prospectively determined using central laboratories employing either a PCR-based or next-generation sequencingbased clinical trial assay using tissue (58%) and/or plasma (65%) samples.

The efficacy population for tepotinib included 69 treatment-naïve patients, and 83 previously treated patients with locally confirmed *MET*ex14 skipping alterations. The bridging study evaluated the clinical validity of F1LCDx as a CDx to identify *MET* exon 14 skipping alteration positive patients with available plasma samples from the VISION trial that tested positive for *MET* exon 14 skipping alteration by the CTAs (CTA+). Additionally, VISION trial enrollment screen-fails that were determined to be negative for *MET* exon 14 skipping alterations by the CTA (CTA-) were tested by F1LCDx.

The concordance between the CTAs and F1LCDx was assessed by positive percent agreement (PPA) and negative percent agreement (NPA). The PPA was 67.20% (84/125) with two-sided 95% confidence interval (CI) [58.56%, 74.81%] and the NPA was 100% (116/116) with two-sided 95% CI [96.79%, 100%] after excluding F1LCDx-unevaluable results when considering both patients that were treatment naïve and previously treated (**Table 82**). Since patients were enrolled and initially tested by local CTAs, the prevalence-adjusted PPVs and NPVs were calculated using the PPA and NPA, after adjusting for the prevalence of *MET* exon 14 skipping alteration among the ITT population (**Table 83**). In the analysis with 3% prevalence, F1LCDx demonstrated an adjusted PPV of 100% with two-sided 95% CI [95.63%, 100%] and NPV of 99.00% with two-sided 95% CI [98.75%, 99.24%].

**Table 82. Contingency Table for MET exon 14 Status Between CTAs (Tissue and Plasma) and F1LCDx**

|  |  | **CTA** |  |
| --- | --- | --- | --- |
|  | **CTA+** | **CTA-** | **Total** |
| **F1LCDx+** | 84 | 0 | 84 |
| **F1LCDx-** | 41 | 116 | 157 |
|  |  | **CTA** |  |
|  | **CTA+** | **CTA-** | **Total** |
| **F1LCDx Unevaluable** | 27 | 3 | 30 |
| **Total** | 152 | 119 | 271 |
| **Agreement Statistics Excluding CDxUnevaluable Results** | PPA:67.20% (84/125)  95% 2-sided CI^1^: [58.56,  74.81] | NPA: 100.00% (116/116) 95% 2-sided CI^1^:  [96.79, 100.00] |  |
| **Percent Unevaluable** | 17.8% (27/152) | 2.52% (3/119) |  |

^1^Calculated with the Wilson-score method 2-sided 95% CI.

**Table 83. Concordance Analysis Results**

|  | **Prevalence** | **Numerator** | **Denominator** | **Point Estimate (%)** | **95% Two-Sided CI* (%)** |
| --- | --- | --- | --- | --- | --- |
| **PPA** | N/A | 84 | 125 | 67.20 | [58.56, 74.81] |
| **NPA** | N/A | 116 | 116 | 100.00 | [96.79, 100.00] |
| **Adjusted PPV** | 3% | N/A | N/A | 100.00 | [95.63, 100.00] |
| **Adjusted NPV** | 3% | N/A | N/A | 99.00 | [98.75, 99.24] |

*CI was calculated using the Wilson-score method for PPA, NPA, and PPV while using the bootstrap method for NPV .

The clinical validity of F1LCDx was demonstrated by assessing clinical efficacy in the F1LCDx *MET*ex14 positive population based on ORR as the primary efficacy endpoint, which is defined as the proportion of patients with best overall response of confirmed Complete Response (CR) or Partial Response (PR) as determined by Blinded independent review committee (BIRC) per Response Evaluation Criteria in Solid Tumors (RECIST) v1.1. To evaluate the secondary efficacy endpoint, duration of response (DOR), the median DOR was calculated by the Kaplan-Meier method along with its two-sided 95% CI for all the patients. The results are reported in **Table 84** and **Table 85** for treatment-naïve patients and previously treated patients, respectively. Because NPA and PPV are 100%, the point estimate of ORR for F1LCDx+ population was the same as that for CTA+/F1LCDx+ population within the respective cohort by treatment lines. The estimated ORR and the corresponding 95% CIs for the F1LCDx+ population in the treatment-naïve cohort and previously treated cohort were 50.0% [34.10%, 65.90%] and 36.96% [23.01%, 50.91%], respectively, which were comparable with the observed ORR for the CTA+ population in the respective cohort by treatment lines.

**Table 84. Efficacy in Treatment-naïve Patients (1L) from NDA Population**

|  | **CTA+** | **F1LCDx+ \| CTA+** | **F1LCDx- \| CTA+** | **F1LCDx**  **evaluable \|**  **CTA+** | **F1LCDx**  **unevaluable \|**  **CTA+** |
| --- | --- | --- | --- | --- | --- |
| **No. of patients** | 69 | 38 | 21 | 59 | 10 |
| **No. of events (CR or PR)** | 30* | 19 | 9 | 28 | 2* |
| **ORR (%)** | 43.48 | 50.00 | 42.86 | 47.46 | 20.00 |
| **95% two-sided**  **CI for ORR (%)** | [32.43,55.21] | [34.85, 65.15] | [24.47, 63.45] | [35.27, 59.96] | [5.67, 50.98] |

*One (1) 1L patient determined as a responder by IRC was not considered a responder by the FDA and was treated as a non-responder in the clinical bridging analysis. Note that this patient was F1LCDx unevaluable.

**Table 85. Efficacy in Previously Treated Patients (2L+) from NDA Population**

|  | **CTA+** | **F1LCDx+ \| CTA+** | **F1LCDx- \| CTA+** | **F1LCDx**  **evaluable \|**  **CTA+** | **F1LCDx**  **unevaluable \|**  **CTA+** |
| --- | --- | --- | --- | --- | --- |
| **No. of patients** | 83 | 46 | 20 | 66 | 17 |
| **No. of events (CR or PR)** | 36* | 17 | 9* | 26* | 10 |
| **ORR (%)** | 43.37 | 36.96 | 45.00 | 39.39 | 58.82 |
| **95% two-**  **sided CI for**  **ORR (%)** | [33.24, 54.09] | [24.52, 51.40] | [25.82, 65.79] | [28.50, 51.45] | [36.01, 78.39] |

*One (1) 2L+ patient determined as a responder by IRC was not considered as a responder by the FDA and was treated as a nonresponder in the clinical bridging analysis. Note that this patient was F1LCDx negative.

To assess the robustness of the data subject to missing F1LCDx test results, a sensitivity analysis was performed, and multiple imputations were used to impute the F1LCDx *MET* exon 14 skipping alteration status in the F1LCDx unevaluable population. The concordance analysis was re-conducted based on the imputed data. The PPA estimates based on the imputed complete datasets has a mean of 67.13% with two-sided 95% empirical CI [64.58%, 69.44%], comparable to the PPA calculated using the observed data (i.e., excluding the F1LCDx-unevaluable samples) which was 67.20% with two-sided 95% Wilson-score CI [58.56%, 74.81%]. The adjusted PPV based on the imputed complete datasets was 100% across all imputations.

The clinical efficacy (ORR) were also re-estimated based on the imputed data. The mean ORR for the F1LCDx+ population was 46.00% with two-sided 95% CI [31.14%, 60.86%] for the treatment-naïve patients, and 39.88% with two-sided 95% CI [26.28%, 53.47%] for the previously treated patients, respectively. The efficacy results after imputation are comparable to those estimated using observed data among treatment-naïve patients and previously treated patients, respectively. In summary, the sensitivity analysis demonstrated the robustness of concordance and efficacy results by accounting for the missing F1LCDx status.

# 11 CDx Classification Criteria

## 11.1 CDx classification criteria for ALK rearrangements, qualifying NSCLC patients for therapy with ALECENSA® (alectinib):

- The *ALK* rearrangement must have pathogenic driver status (Foundation Medicine driver status of “known” or “likely”)
- AND the disease type must be NSCLC
- AND one of the following two conditions must hold:
  1. The partner gene is *EML4*, or
  2. The *ALK* breakpoint occurs within *ALK* intron 19

## 11.2 CDx classification criteria for EGFR alterations, qualifying NSCLC patients for therapy with EGFR Tyrosine Kinase Inhibitors (TKI) approved by FDA:

- Base substitutions resulting in *EGFR* L858R
- In-frame deletions occurring within *EGFR* exon 19

## 11.3 CDx classification criteria for BRCA1, BRCA2, and ATM alterations, qualifying prostate cancer patients for therapy with LYNPARZA® (olaparib):

**Table 86** describes the criteria for classifying *BRCA1*, *BRCA2, or ATM* alterations known to be deleterious to protein function.

Table 86. Classification Criteria for BRCA1, BRCA2, and ATM

| **Genes**  **(Transcript)** | **Variant class** | **Biomarker description** |
| --- | --- | --- |
| ATM  (NM_000051)  BRCA1  (NM_007294)  BRCA2  (NM_000059) | Short variant | Any nonsense^1^, frameshift, or splice site^2^ variant  • For BRCA2, truncating mutations must occur upstream of bases encoding amino acid 3326  Any of the ATM, BRCA1, or BRCA2 alterations listed in **Table 87, Table 88**, and **Table 89.** |
|  | Copy number | Homozygous deletion of one or more exons, regardless of transcript  • F1LCDx will only include copy number loss in BRCA1 and BRCA2 |
|  | Rearrangement | Any inactivating rearrangement, regardless of transcript |

1. Missense mutations in the start codon (except for those in the appendix tables) and short variant deletions spanning from upstream of the start codon (annotated as M1?) are biomarker negative.
2. This rule is limited to splice site variants within the donor or acceptor sites, defined as the first two or last two bases of the intron.

Table 87. List of short variants in ATM NM_000051

| M1I | D2708N |
| --- | --- |
| M1L | V2716A |
| M1T | G2765S |
| P292L | F2827C |
| D2016G | R2832C |
| R2032K | S2855_V2856>RI |
| A2067D | D2913Y |
| R2227C | R3008C |
| Y2470D | R3008H |
| R2547_S2549del | splice site 331+5G>A |
| A2622V | splice site 8418+5_8418+8delGTGA |
| D2625_A2626>EP |  |

Table 88. List of short variants in BRCA1 NM_007294

| M1I | C44Y | R1495K | D1692Y | K1759N | Y1853C |
| --- | --- | --- | --- | --- | --- |
| M1R | C47F | R1495M | C1697R | L1764P | C1787_G1788>SD |
| M1T | C47S | R1495T | R1699Q | I1766N | splice site 212+3A>G |
| M1V | C47Y | E1559K | R1699W | I1766S | splice site 213-11T>G |
| M18T | C61G | E1559Q | L1705P | G1770V | splice site 213-12A>G |
| L22S | C61S | A1623G | G1706E | M1775K | splice site 302-3C>G |
| C24R | C61Y | S1655F | G1706R | M1775R | splice site 4986+3G>C |
| I26N | C64G | T1685A | A1708E | L1780P | splice site 4986+5G>A |
| T37K | C64R | T1685I | S1715N | C1787S | splice site 4986+6T>C |
| C39G | C64W | H1686R | S1715R | G1788V | splice site 4986+6T>G |
| C39R | C64Y | V1688del | W1718C | P1812A | splice site 5074+3A>G |
| C39W | R71G | M1689R | S1722F | A1823T | splice site 5194-12G>A |
| C39Y | R71K | T1691I | V1736A | V1833M | splice site 5406+4A>G |
| H41R | R71M | T1691K | V1736G | W1837C |  |
| C44F | R71T | D1692H | G1738E | W1837R |  |
| C44S | S770L | D1692N | G1738R | V1838E |  |

Table 89. List of short variants in BRCA2 NM_000059

| M1I | V211L | W2626C | L2686P | G2748D | splice site 316+4delA |
| --- | --- | --- | --- | --- | --- |
| M1R | Y600C | I2627F | L2688P | G2793R | splice site 316+5G>A |
| M1T | K1530N | L2653P | T2722K | A2911E | splice site 8487+3A>G |
| M1V | R2336H | R2659G | T2722R | E3002K | splice site 8754+3G>C |
| D23N | R2336L | R2659K | D2723A | R3052W | splice site 8754+4A>G |
| D23Y | R2336P | R2659T | D2723G | G3076V | splice site 8754+5G>A |
| S142I | T2412I | Y2660D | D2723H | D3095E |  |
| S142N | L2510P | E2663V | D2723V | D3095G |  |
| V159M | R2602T | S2670L | G2724W | N3124I |  |
| V211I | H2623R | I2675V | Y2726C | N3187K |  |

## 11.4 CDx classification criteria for BRCA1 and BRCA2 alterations, qualifying prostate cancer patients for therapy with RUBRACA® (rucaparib):

**Table 90** describes the criteria for classifying *BRCA1* or *BRCA2* alterations known to be deleterious to protein function.

Table 90. Classification Criteria for BRCA1 and BRCA2

| **Genes (Transcript)** | **Variant class** | **Biomarker description** |
| --- | --- | --- |
| BRCA1  (NM_007294)  BRCA2  (NM_000059) | Short variant | Any nonsense^1^, frameshift, or splice site^2^ variant  • For BRCA2, truncating mutations must occur upstream of bases encoding amino acid 3326  Any of the BRCA1 or BRCA2 alterations listed in **Table 88** and **Table 89.** |
|  | Copy number | Homozygous deletion of one or more exons, regardless of transcript |
|  | Rearrangement | Any inactivating rearrangement, regardless of transcript |

1. Missense mutations in the start codon (except for those in the appendix tables) and short variant deletions spanning from upstream of the start codon (annotated as M1?) are biomarker negative.
2. This rule is limited to splice site variants within the donor or acceptor sites, defined as the first two or last two bases of the intron.

## 11.5 CDx classification criteria for PIK3CA alterations, qualifying breast cancer patients for therapy with PIQRAY® (alpelisib):

Presence of *PIK3CA* mutation(s): H1047R; E545K; E542K; C420R; E545A; E545D [1635G>T only]; E545G; Q546E; Q546R; H1047L; or H1047Y

## 11.6 CDx classification criteria for SNVs and Indels that lead to MET exon 14 skipping:

A SNV or indel in *MET* shall be considered to result in skipping of exon 14 if one or more of the following criteria are met:

1. Deletions greater than or equal to 5 bp that affect positions -3 to -30 in the intronic region

immediately adjacent to the splice acceptor site at the 5′ boundary of *MET* exon 14.

1. Indels affecting positions -1 or -2 at the splice acceptor site of the 5′ boundary of *MET* exon 14.
2. Base substitutions and indels affecting positions 0, +1, +2, or +3 at the splice donor site of the 3′

boundary of *MET* exon 14.

## 11.7 CDx classification criteria for NTRK fusions:

Rearrangements in *NTRK1*, *NTRK2*, or *NTRK3* shall be considered CDx biomarker positive, that is, to lead to a *NTRK1*, *NTRK2*, or *NTRK3* RNA fusion, if the following criterion is met:

• In-strand rearrangement events that may lead to an *NTRK1*, *NTRK2* or *NTRK3* RNA fusion with a previously reported or novel partner gene in which the kinase domain is not disrupted. This also includes rearrangement events that result in reciprocal fusions (*NTRK* may be on either the 5' or the 3' end of the detected fusion).

In this regard out-of-strand events are considered as non-fusion rearrangements and are classified as CDx biomarker-negative. Intragenic fusions in which genomic rearrangement events are wholly internal to the *NTRK1*, *NTRK2*, or *NTRK3* genes (i.e., *NTRK1*-*NTRK1*, *NTRK2*-*NTRK2*, *NTRK3*-*NTRK3* events) are also considered biomarker-negative. Unidentified partners (encoded as N/A) or LINC non-coding partners are also considered CDx biomarker-negative.

## 11.8 CDx classification criteria for ROS1 fusions

Rearrangements in *ROS1* shall be considered CDx biomarker positive, i.e., to lead to *ROS1* RNA fusion, if the following condition is met:

• In-strand rearrangement events that may lead to a *ROS1* RNA fusion with another protein coding gene in which the *ROS1* kinase domain is not disrupted. *ROS1* must be on the 3′ end of the detected fusion.

In this regard, out-of-strand events are considered as non-fusion rearrangements and are classified as CDx biomarker-negative. Intragenic fusions in which genomic rearrangement events are wholly internal to the *ROS1* (i.e., *ROS1*-*ROS1* events) are also considered biomarker-negative. Unidentified partners (encoded as N/A) or LINC non- coding partners are also considered CDx biomarker-negative. *ROS1* fusions with novel partners are required to be in frame.

## 11.9 CDx classification criteria for BRAF V600E

• Base alterations resulting in *BRAF* V600E

## 11.10 CDx classification criteria for BRCA1 and BRCA2 alterations, qualifying prostate cancer

### patients for therapy with AKEEGA® (niraparib + abiraterone acetate)

**Table 91** describes the criteria for classifying *BRCA1* or *BRCA2* alterations known to be deleterious to protein function.

**Table 91. Classification Criteria for BRCA1 and BRCA2**

| **Genes**  **(Transcript)** | **Variant class** | **Biomarker description** |
| --- | --- | --- |
| BRCA1  (NM_007294)  BRCA2  (NM_000059) | Short variant | Any nonsense^1^, frameshift, or splice site^2^ variant  • For BRCA2, truncating mutations must occur upstream of bases encoding amino acid 3326  Any of the BRCA1 or BRCA2 alterations listed in **Table 88** and **Table 89.** |
|  | Copy number | Homozygous deletion of one or more exons, regardless of transcript |
|  | Rearrangement | Any inactivating rearrangement, regardless of transcript |

1. Missense mutations in the start codon (except for those in the appendix tables) and short variant deletions spanning from upstream of the start codon (annotated as M1?) are biomarker negative.
2. This rule is limited to splice site variants within the donor or acceptor sites, defined as the first two or last two bases of the intron.

## 11.11 CDx classification criteria for BRCA1 and BRCA2 alterations, qualifying prostate cancer

### patients for therapy with LYNPARZA® in combination with abiraterone

**Table 92** describes the criteria for classifying *BRCA1* or *BRCA2* alterations known to be deleterious to protein function.

**Table 92. Classification Criteria for BRCA1 and BRCA2**

| **Genes (Transcript)** | **Variant class** | **Biomarker description** |
| --- | --- | --- |
| BRCA1 (NM_007294)  BRCA2 (NM_000059) | Short variant | Any nonsense^1^, frameshift, or splice site^2^ variant  • For BRCA2, truncating mutations must occur upstream of bases encoding amino acid 3326  Any of the BRCA1 or BRCA2 alterations listed in **Table 88** and **Table 89**. |
|  | Copy number | Homozygous deletion of one or more exons, regardless of transcript |
|  | Rearrangement | Any inactivating rearrangement, regardless of transcript |

1. Missense mutations in the start codon (except for those in the appendix tables) and short variant deletions spanning from upstream of the start codon (annotated as M1?) are biomarker negative.
2. This rule is limited to splice site variants within the donor or acceptor sites, defined as the first two or last two bases of the intron.

## 11.12 CDx classification criteria for *PIK3CA* alterations, qualifying breast cancer patients for therapy with ITOVEBI™ (inavolisib) in combination with with palbociclib and fulvestrant

Plasma samples meet the biomarker eligibility requirements to assign patients to treatment with inavolisib plus palbociclib and fulvestrant if any of the short variant alterations listed in **Table 93** below is detected.

**Table 93. List of Eligible PIK3CA Short Variant Alterations**

| R88Q | N345D | E453G | E542V | Q546H | H1047L | G1049R |
| --- | --- | --- | --- | --- | --- | --- |
| G106A | N345H | E453K | E545A | Q546K | H1047N | G1049S |
| G106D | N345I | E453Q | E545D | Q546L | H1047P |  |
| G106R | N345K | E453V | E545G | Q546P | H1047Q |  |
| G106S | N345S | E542A | E545K | Q546R | H1047R |  |
| G106V | N345T | E542D | E545L | M1043I | H1047T |  |
| K111N | N345Y | E542G | E545Q | M1043T | H1047Y |  |
| K111R | C420R | E542K | E545R | M1043V | G1049A |  |
| K111E | E453A | E542Q | E545V | H1047D | G1049C |  |
| G118D | E453D | E542R | Q546E | H1047I | G1049D |  |

3100 Hanover Street
Palo Alto, CA 94304

1.855.698.8887

[clientservices@guardanthealth.com](mailto:clientservices@guardanthealth.com)


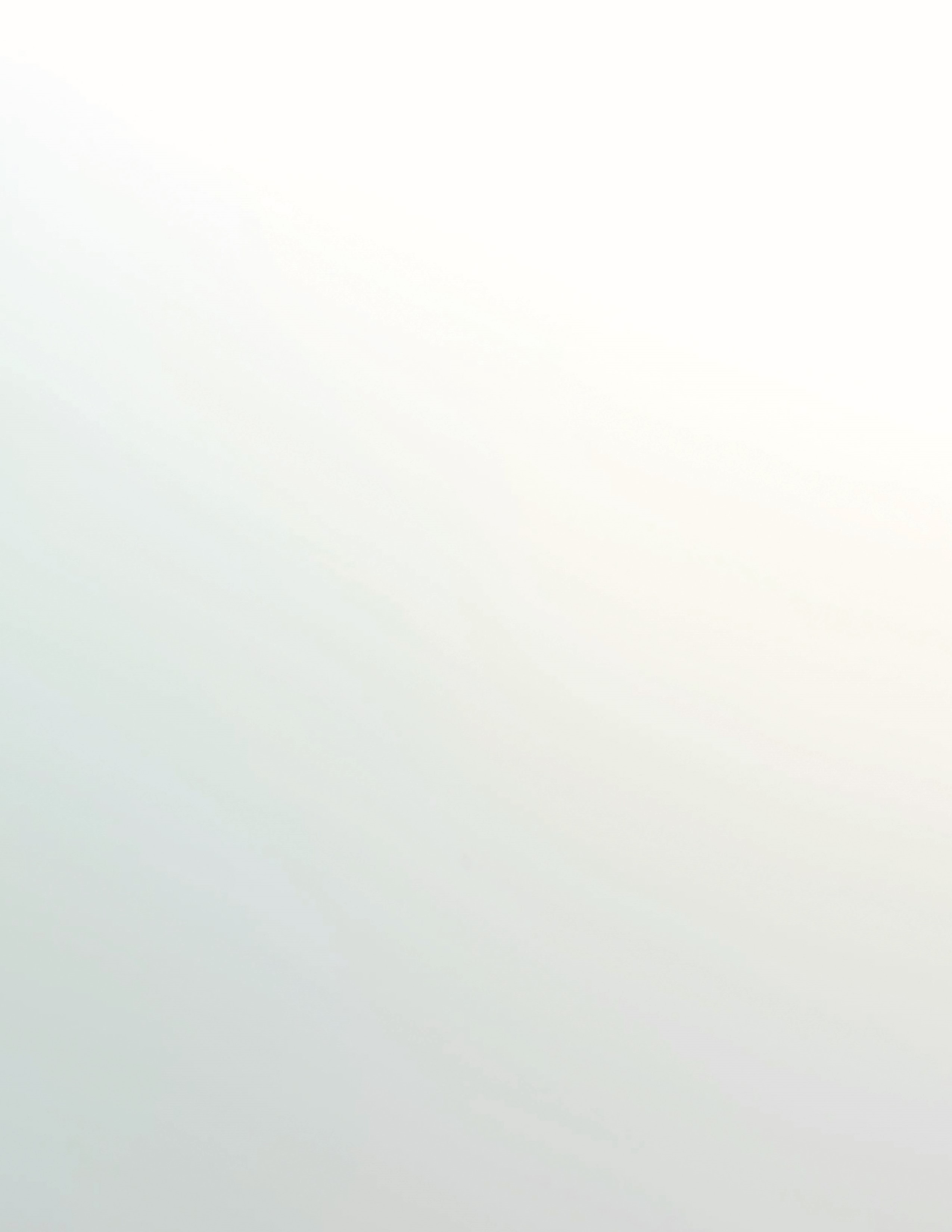

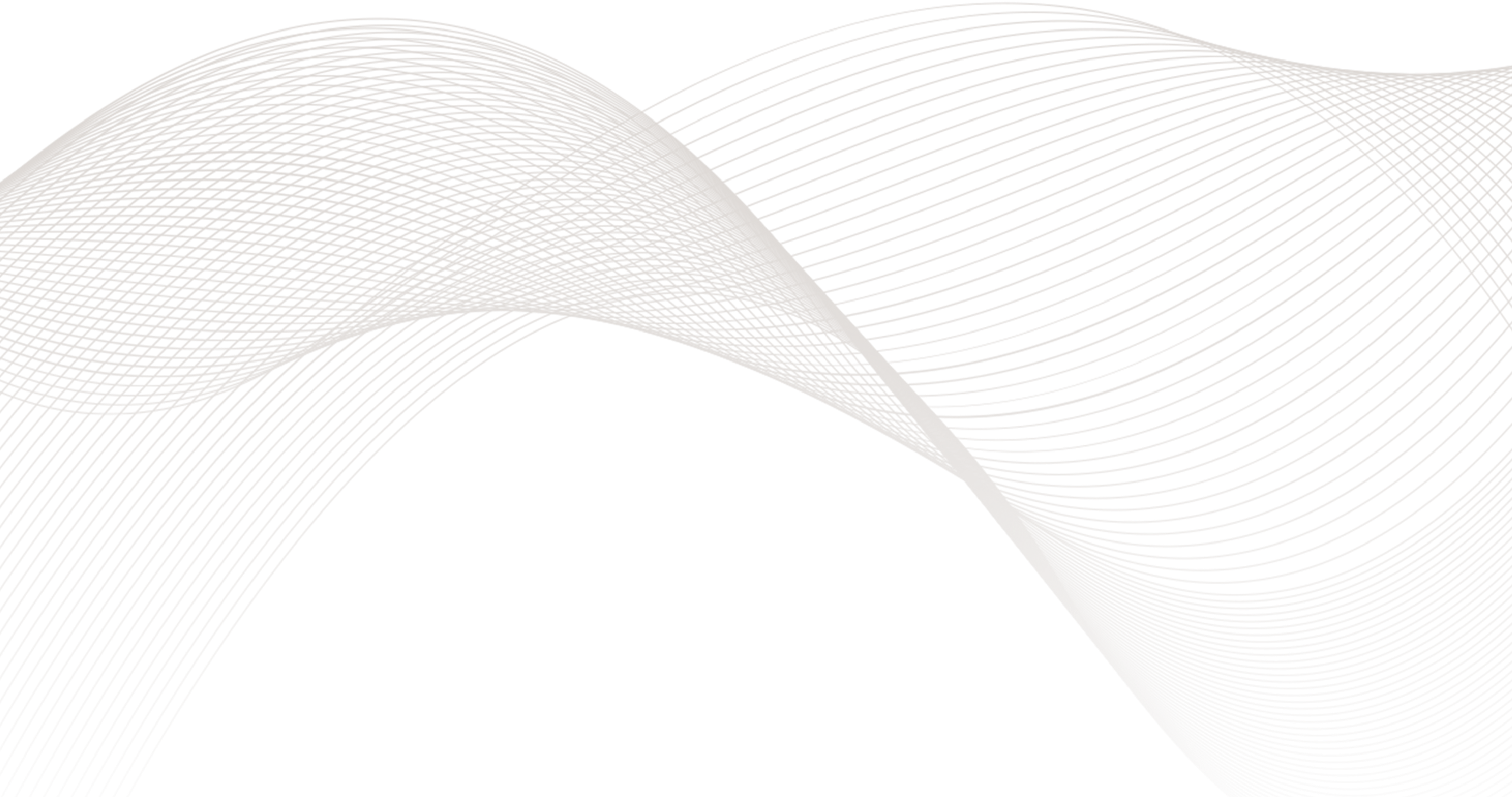


Quantifying ctDNA Using

a Tissue-Free Test for

Minimal Residual Disease

(

MRD) Detection

**Important Note:**

Guardant Reveal on Infinity was developed, and its performance

characteristics were determined, by the Guardant Health Clinical Laboratory in Redwood

City, CA, USA, which is certified under the Clinical Laboratory Improvement Amendments

of 1988 (CLIA) as qualified to perform high-complexity clinical testing. Guardant Reveal is

a Laboratory Developed Test.

# Background

Minimal Residual Disease (MRD) describes the presence of tumor cells after a curative intent surgery or therapy. Genomic and/or epigenomic MRD assays have been developed to identify circulating tumor DNA (ctDNA) in blood from patients with earlystage solid tumors. The presence of ctDNA after the completion of curative intent treatment is prognostic, indicating a high likelihood of cancer recurrence.^1,2^ Additionally, emerging data indicate that initiating adjuvant treatment may reduce the risk of recurrence for patients with ctDNA detected after surgery.^3^

Guardant Infinity is the first commercially available platform that integrates epigenomics and genomics to transform precision oncology. In this context, we describe how Guardant Reveal, a tissue-free, MRD monitoring test that analyzes thousands of epigenetic signals, detects and quantifies ctDNA in patients with early-stage cancer using the Guardant Infinity platform.

# Methylation and Cancer

Genomic alterations significantly contribute to carcinogenesis by activating oncogenes, inactivating tumor suppressor genes, and inducing genomic instability. Epigenomic alterations also play a significant role in carcinogenesis by affecting gene expression.^4^ Methylation, a chemical modification of DNA, is one type of epigenomic alteration.^4^ Methylation can serve as an early driver of tumorigenesis by either silencing tumor suppressor genes or activating oncogenes.^5^

Compared to normal cells, tumor cells exhibit a distinct methylation pattern.^6^ Analyzing differentially methylated regions (DMRs) associated with specific solid tumors can differentiate normal cell-free DNA (cfDNA) from cancer-derived ctDNA (Figure 1).

## Figure 1. Identification of DMRs to detect ctDNA


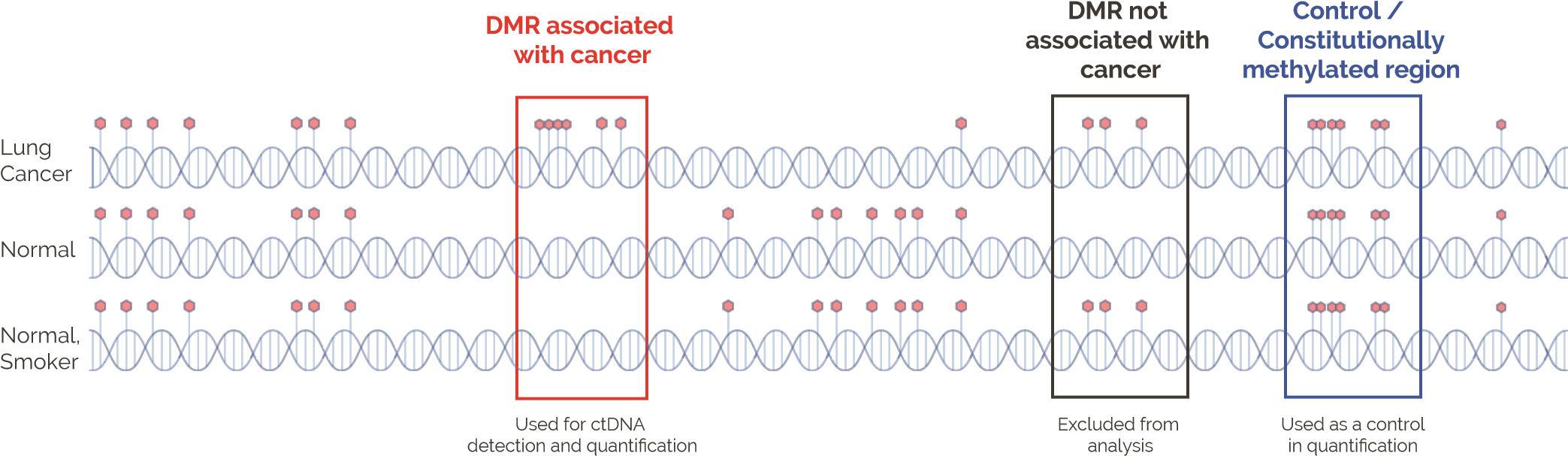


Informative methylation regions were identified by comparing normal vs. cancer samples from large cohorts

Guardant Reveal evaluates DMRs to identify ctDNA. To develop this approach, Guardant Health analyzed nearly 5,000 samples from both cancer-free donors and from patients with cancer to identify DMRs that accurately and reliably identify ctDNA molecules in plasma samples. Regions of hypermethylation distinctive to cancer samples and absent in cancer-free samples were identified from over 1,700 patients with colorectal, breast, lung, or bladder cancer and 3,000 cancer-free donors, including patients with common comorbidities. These distinctive regions are utilized to identify and detect ctDNA with Guardant Reveal.

# Guardant Reveal Employs a Novel Method for Methylation Analysis and ctDNA detection

Bisulfite sequencing is the most common method for analyzing DNA methylation, but requires harsh chemical treatment, leading to significant molecule loss and low sequencing efficiency.^7^ Molecule loss impairs assay performance in the context of MRD, where low tumor burden is typical. To achieve highly sensitive detection of ctDNA through methylation analysis, Guardant Health developed the Guardant Infinity platform. Guardant Infinity’s proprietary technology separates methylated from non-methylated cfDNA molecules and preferentially enriches methylated tumor DNA, minimizing molecule loss and reducing background noise (Figure 2). Guardant Reveal utilizes this technology for MRD detection.


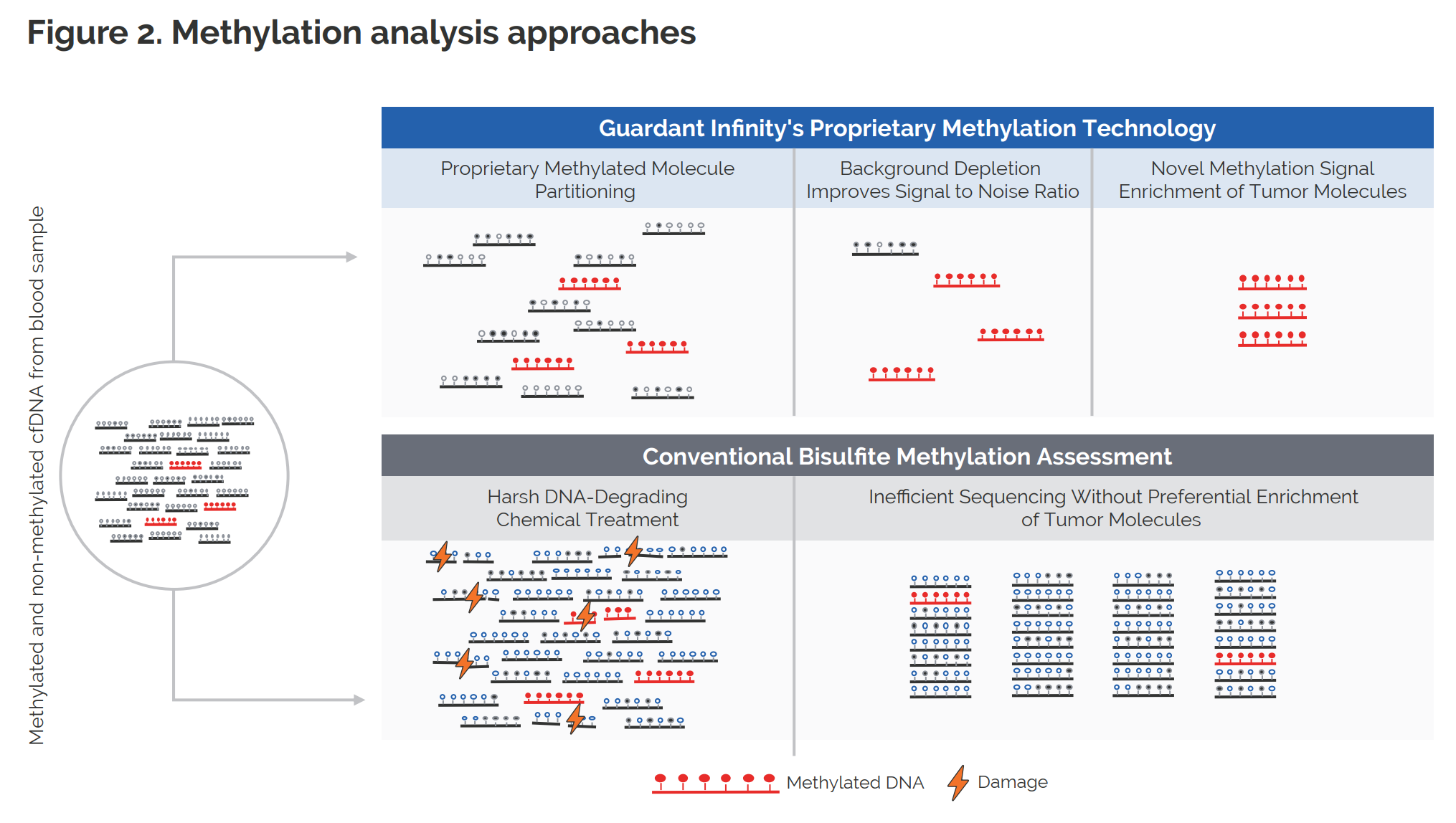


Guardant Reveal analyzes cell-free DNA (cfDNA) extracted from plasma to determine the presence and quantity of tumor-associated cfDNA, eliminating the need for tumor tissue or separate sequencing of peripheral blood mononuclear cells (PBMCs). Briefly, the process is as follows:

1. Using automated methods, cfDNA is extracted from plasma, partitioned based on its methylation state, repaired, ligated with custom-made adapters for molecular tracking, and then amplified to create libraries.
2. The libraries are subsequently enriched for specific regions using an epigenomic panel that targets tumor-associated DNA methylation signatures.
3. The enriched libraries undergo next-generation sequencing. The sequencing data is analyzed using Guardant Health’s proprietary bioinformatics pipeline software (BIP), which is trained to detect the presence of ctDNA based on epigenomic signals.
4. Following the bioinformatics analysis, Guardant Reveal reports either “ctDNA detected” along with an estimated tumor fraction, or “ctDNA not detected”.

#
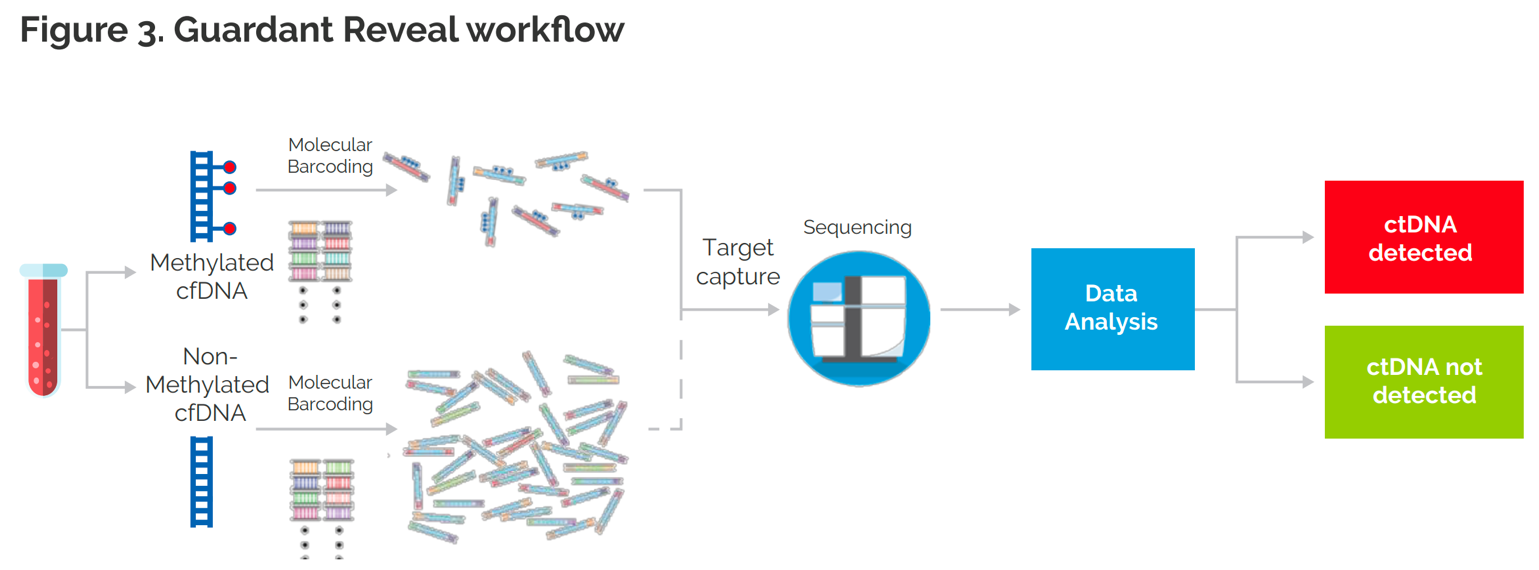


# Quantification of Tumor Fraction on Guardant Reveal Powered by Guardant Infinity

The presence or absence of ctDNA after completion of curative intent therapy has been shown to predict cancer recurrence. Quantifying ctDNA to monitor tumor burden and ctDNA dynamics is increasingly important. A patient’s tumor burden can be estimated by comparing the fraction of ctDNA to the total cfDNA molecules in a sample. The maximum genomic allele fraction (maxMAF) of a genomic assay can be used to quantify tumor burden.

However, this method may have limited precision and accuracy in the early-stage setting, where tumor shedding is lower than in the metastatic setting. Tumor fraction can also be derived from methylation assays, such as Guardant Reveal, by assessing the ratio of methylated molecules in differentially methylated regions compared to the total number of methylated molecules in constitutively methylated regions (Figure 4). Tumor fraction derived from methylation reduces the impact of clonal hematopoiesis of indeterminate potential (CHIP) and biological noise, thus improving precision and accuracy.


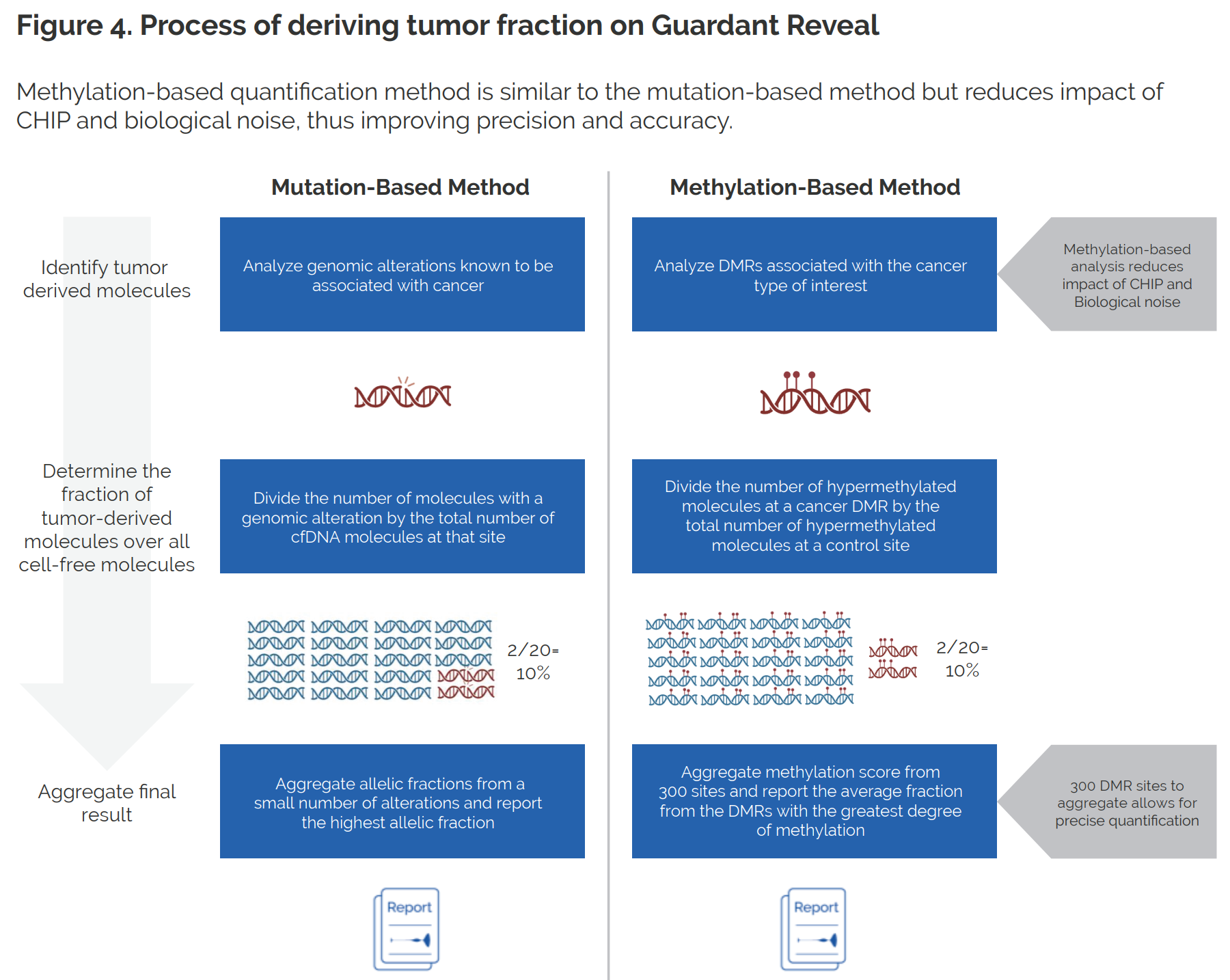


Briefly, the process of generating tumor fraction through a methylation-based method is as follows:

- In a patient’s sample, cancer specific DMRs are normalized with appropriately matched control regions. This normalization is achieved by dividing the methylated molecule count for each cancer DMR by the methylated molecule count of the matched control region. The matched control region is identified as a region with similar opportunity for methylation across samples and patients.
- A methylation percentage is calculated for each cancer-specific DMR, derived from the count of methylated molecules.
- The DMRs with the highest scores are selected and averaged to calculate the tumor fraction.

# Early Insights: Guardant Reveal and Tumor Fraction

In an interim analysis of COSMOS*-CRC-01 utilizing Guardant Reveal^8^, which included over 130 patients and more than 700 samples, all patients were evaluable for MRD analysis without the need for tumor tissue. This cohort included patients with R0 stage II or III colon cancer. When assessing outcomes at the 28-day, post-operative time point, a significant difference was observed in 24-month recurrence-free survival between patients with detected ctDNA and those without (Figure 5A).

Additionally, surveillance monitoring, which involved patients with two or more postsurgery samples, was conducted in 134 patients, providing early insights into tumor fraction relative to treatment and recurrence. Among 12 patients who experienced recurrence, each having at least two post-surgery samples, the estimated tumor fraction increased as the time of recurrence approached (Figure 5B). Tumor fraction was also influenced by adjuvant therapy, as evidenced in select patient examples below.

For patients who had more than two post-surgery samples and were included in the surveillance analysis, a high sensitivity of 80% for detecting recurrence was achieved, with a specificity of 98%**. This interim analysis from a large, prospective ctDNA monitoring study demonstrates sensitive and specific MRD detection and tumor fraction quantification in stage II-III colon cancer using a tissue-free epigenomic assay.

* The COSMOS acronym stands for Conquer Solid Malignancies by blood screening ** Among samples for those with at least one year of follow-up.

## Figure 5. Recurrence-free survival (RFS) and Tumor Fraction from clinical validation cohort

1. Recurrence-free survival from single, post-operative time point prior to ACT, if given (n=135)


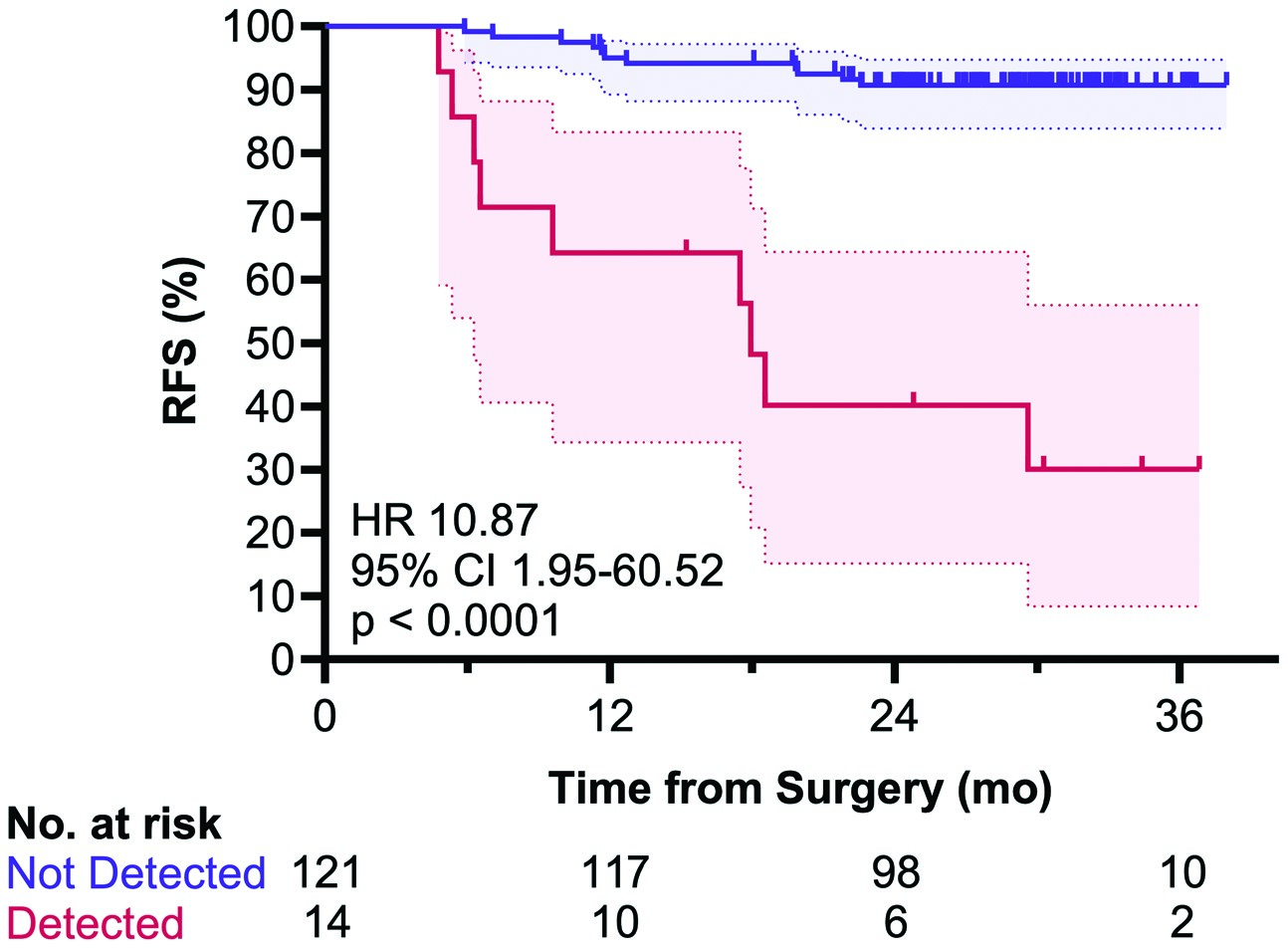


| **ctDNA Status** | Not Detected | Detected |
| --- | --- | --- |
| **No. of Events / Total No.** | 11 / 121 | 9 / 14 |
| **Median RFS** | Not Reported | 18.0 mo |
| **24M RFS (95% CI)** | 90.7%  (83.9-94.8) | 40.2% (15.1-64.4) |

1. Tumor Fraction trends concordant with genomics, treatment, and recurrence

**Tumor fraction**


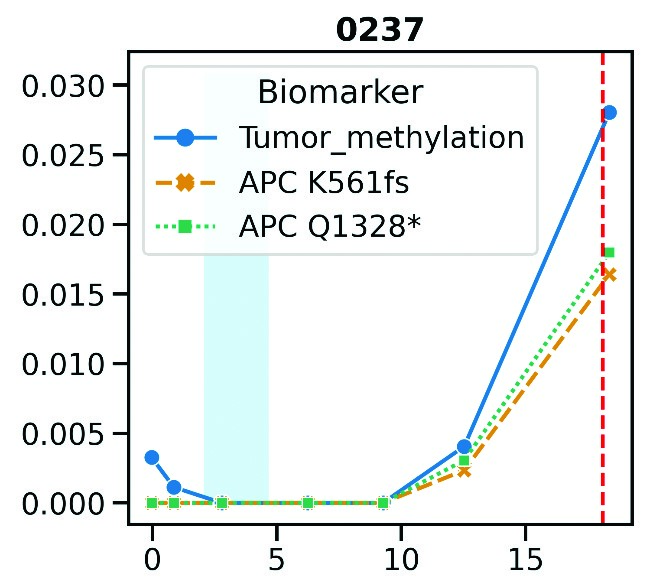

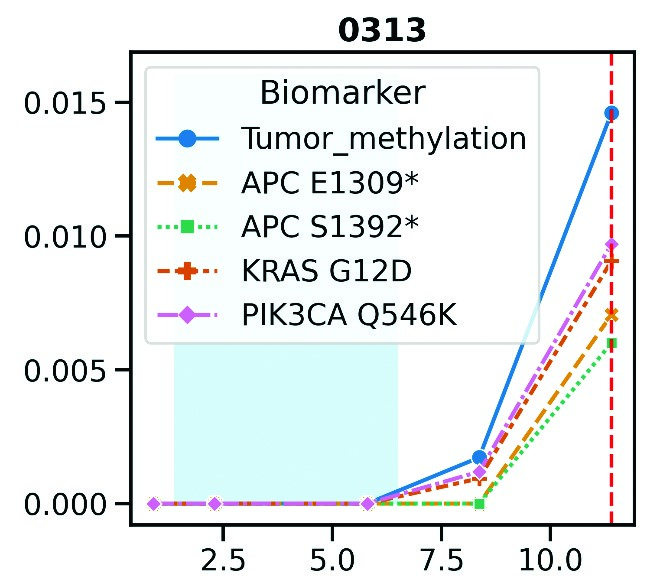

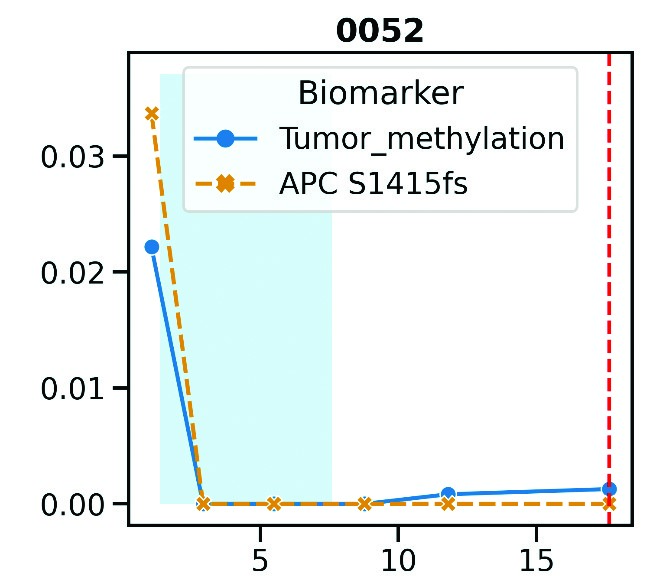


### Months since surgery

Notes: ”Tumor_methylation” is tumor fraction and can be tracked by the blue line. The blue box defines when patient received adjuvant therapy. The red, dotted, vertical line is when recurrence occurred.*Denotes mutation type and means the protein is now a stop codon, which is annotated here as an asterisk.

# Method and Limitations

Guardant Reveal is a next generation sequencing (NGS)-based assay for the detection of cancer-derived circulating tumor DNA (ctDNA) in patients with colorectal cancer (CRC), breast cancer, and lung cancer. Cell-free DNA (cfDNA) is extracted from plasma, sequenced, and the data analyzed for the presence of epigenomic alterations in cancer. Based on this analysis, the Guardant Reveal test returns a result of either:

ctDNA detected or ctDNA not detected. A predicted tumor fraction (TF) based on epigenomic signals is included if ctDNA is detected. The analytical sensitivity of the assay is 95% at the limit of detection (0.005% T F for CRC with minimum cfDNA input).

The Guardant Reveal test is not designed to provide comprehensive genomic profiling results. Certain biological and clinical factors can impact the sensitivity and/or specificity of this test including treatment history, second primary tumor, pregnancy, rate of any residual tumor growth, tumor size, number of somatic alterations in the tumor, vascularization, and/or location. Guardant Reveal results should be interpreted in the appropriate clinical context and patients should continue participating in guideline-recommended programs for minimal residual disease monitoring and recurrence detection.


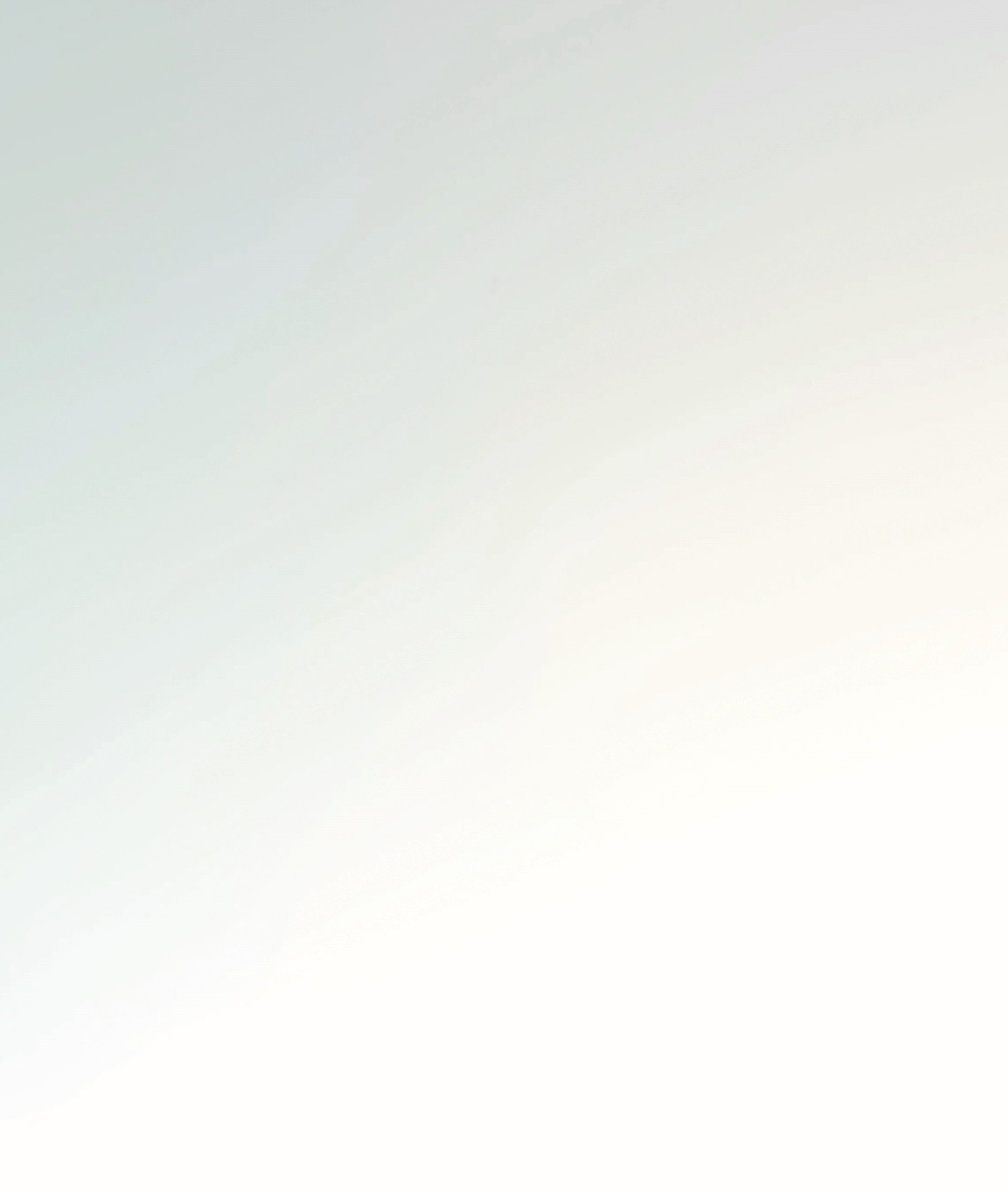

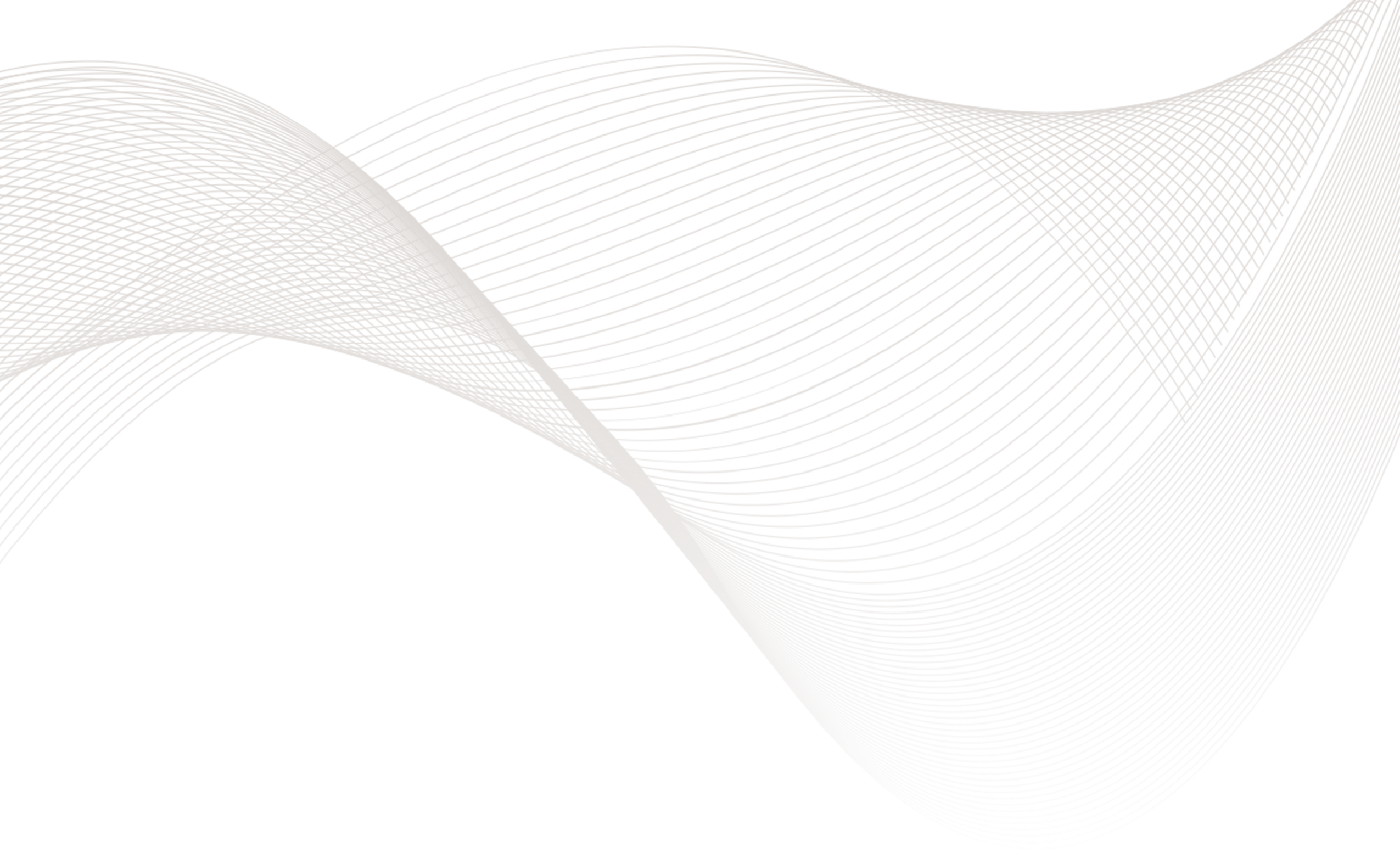


**References**

1.

Parikh et al. 2021. https://doi.org/10.1158/1078-0432.CCR-21-0410

2.

Tie et al. 2020. https://doi.org/10.1002/ijc.33312

3.

Tie et al. 2022. https://doi.org/10.1056/nejmoa2200075

4.

Jones et al. 2014. https://doi.org/10.1016%2Fj.cell.2007.01.029

5.

Lakshminarasimhan et al. 2020. https://doi.org/10.1007%2F978-3-319-43624-1_7

6.

Liu, P. 2021. https://doi.org/10.3389%2Ffgene.2021.798748

7.

Genereux et al. 2008. https://doi.org/10.1093%2Fnar%2Fgkn691

8.

Nakamura et al. ASCO GI 2024 Abstract #180

© 2024 Guardant Health, Inc. All rights reserved. MKT-000316 R1

Summary

•

Guardant Reveal leverages the Guardant Infinity platform to preserve methylated regions in

cell-free DNA, enabling highly efficient detection of minimal residual disease.

•

Guardant Reveal calculates ctDNA tumor fraction from the Guardant Infinity platform using

an epigenomics approach, determined by comparing the degree of methylation across

thousands of DMRs compared with that in controls.

•

Early analysis demonstrates the ability to detect and report tumor fraction clinically using an

epigenomics-based assay.

•

Guardant Health continues to lead liquid biopsy technology with the Guardant Infinity

platform, including accelerating advancements in the field of minimal residual disease with

Guardant Reveal.

1. Confirmation of LoD was performed without direct LoD establishment data. Platform LoD was used for the targeted dilution level. [↑](#footnote-ref-1)
2. Confirmed LoD for BRCA1 RE was using the DIBv1 primer set. LoD was also confirmed using the DIBv2 primer set at 1.27% VAF. [↑](#footnote-ref-2)
