# Supplementary material for: ctDNA guided management of POLE mutant GI malignancies promotes exceptional responses and prolonged survival to immunotherapy
Source: Front Immunol. 2026 Feb 17;17:1738295. doi: 10.3389/fimmu.2026.1738295 (PMC12953476; doi:10.3389/fimmu.2026.1738295)
Supplement: Supplementary file 2 [file Table1.docx]

**Supplementary Table 1:** Variants of unknown significance detected in patient # 1’s liquid biopsy specimen prior to commencing systemic treatment.

| ﻿***ATM***  R2854C | ﻿***BCORL1***  V694M | ﻿***CD22***  amplification | ﻿***MST1R***  S708R |
| --- | --- | --- | --- |
| ***NTRK3***  R201H | ﻿***PIK3CB***  Y246D | ﻿***SPEN***  R229P | ***TSC2***  A583T |

**Supplementary Table 2A:** Miscellaneous genetic aberrations detected in patient # 2’s liquid biopsy sample prior to commencing systemic treatment.

| ﻿﻿***APC***  S1276*, H1490fs*24,  E1309*, S1400L | ﻿***AR***  Q24H | ﻿***ATM***  S2549*, H2125R | **ATR**  E148* |
| --- | --- | --- | --- |
| ***AXIN1***  R146Q | ﻿***DNMT3A***  Q816* | ﻿***ERRFI1***  E416* | ﻿***FUBP1***  Y545* |
| ﻿***JAK3***  M511I | ﻿***MLL2***  L3984* | ﻿***PDGFRA***  K770N | ﻿***PIK3CA***  E542K, R357Q |
| ﻿***PTEN***  R130Q | ***RB1***  E125* | ﻿***SETD2***  E743* | ***TBX3***  E111*, splice site 942-1G>A |
| ***TP53***  D49N | ﻿ |  |  |

| ﻿***﻿AKT3***  K364T | ***ALK***  R401* | ﻿***APC***  I1417S, L2198W, L752R | ﻿***ARID1A***  R2024W |
| --- | --- | --- | --- |
| ***ATM***  L1598I | ﻿***ATR***  D1047Y, N1668T | ﻿***ATRX***  K1225T | ***BCL2***  F212L |
| ***BRCA1***  F1316L, K991Q | ﻿***BRCA2***  F2200L, F2794L, K1691Q, N905H, R2389I, T2197P | ﻿***CCNE1***  L188V | ***CD22***  T615A |
| ***CD274 (PD-L1)***  A85V | ﻿***CREBBP***  E2404K | ﻿***CTNNA1***  R98Q | ***EPHA3***  A411V |
| ﻿***EPHB1***  V562I | ﻿***ERG***  A387T | ﻿***FGFR1***  F114L, K655T, R209C | ﻿***GRM3***  F630L |
| ﻿***IKZF1***  L85R | ﻿***KEL***  R292Q | ﻿***MAP3K1***  R854C, S1255Y | ﻿***MAP3K13***  S399Y |
| ﻿***MET***  F931L, S916Y, Y1234* | ﻿***MITF***  D227N | ﻿***MLL2***  S2115I, T4271A | ﻿***NBN***  R218I |
| ﻿***NKX2-1***  G322S | ﻿***NTRK1***  E413K | ﻿***PALB2***  E554K | ﻿***PDGFRA***  E301* |
| ﻿***PDGFRB***  L42F | ﻿***PPP2R1A***  S587F | ﻿***PTEN***  K197N | ﻿***QKI***  S217Y |
| ﻿***RAF1***  S529L | ﻿***RPTOR***  R788C | ﻿***SETD2***  D1585N | ﻿***SOX2***  L81R |
| ﻿***TEK***  F279L | ﻿***TET2***  K110N | ﻿***TNFAIP3***  I207S |  |

**Supplementary Table 2B:** Variants of unknown significance detected in patient # 2’s liquid biopsy specimen prior to commencing systemic treatment.

**Supplementary Table 3A:** Miscellaneous genetic aberrations detected in subject # 3’s liquid biopsy sample prior to commencing systemic treatment.

| ﻿**APC**  E1309*, S1189*, R1114*, R2237*, R2204*, Y2645* | ﻿**ATR**  E254*, R1886*, R1015Q | **BARD1**  E550* | ﻿**CASP8**  R430Q |
| --- | --- | --- | --- |
| ﻿﻿ ﻿ ﻿**CDC73**  R484C | ﻿**CTNNA1**  E359* | ﻿**EZH2**  splice site 2195+1G>A | ﻿**FAS**  R250* |
| **FBXW7**  splice site 1122+2T>C,  E369* | ﻿**KMT2A (MLL)**  R1976Q | ﻿**MAP2K4**  E203* | **MAP3K1**  E874* |
| ﻿**MLL2**  R2734* | ﻿**MSH2**  E483* | ﻿**MSH3**  E691* | **MSH6**  E908* |
| ﻿**MYCN**  R373Q | ﻿ **﻿NBN**  E383* | ﻿**PIK3R1**  R461* | **PTEN**  R173H, E299* |
| ﻿**RB1**  E137*, E125*, E54*,  E323*, R552* | ﻿**SF3B1**  R831Q | **SMAD2**  R120Q | **SMARCA4**  R381Q |
| ﻿**SPEN**  R1366* | ﻿ **﻿TP53**  R306*, S127P, R213Q | ﻿**WT1**  Y261* |  |

**Supplementary Table 3B:** Variants of unknown significance detected in patient # 3’s liquid biopsy specimen prior to commencing systemic treatment.

| ﻿***AKT2***  Q104R | ﻿***AKT3***  A106S, L77F | ﻿***ALK***  D605G, E1161K, S1034L, S1501N | ﻿***APC***  F2099C, L2092I, S1861Y,  S1971Y, T1073A |
| --- | --- | --- | --- |
| ﻿***AR***  K826T, R753Q,  S207N, Y764* | ﻿***ASXL1***  E865K, G1347D, K1202N, V335G | ﻿***ATM***  E1009D, L1408F, R2691C | ***TR***  E1918K, F2072C, F2436S, K1595N, K483N, L1155V, N811H, R1179I, S1465Y, S727Y |
| ﻿***ATRX***  E2015K, K923T | ﻿***AURKA***  E260D, N40S | ﻿***AXIN1***  E195* | ﻿***AXL***  D519A, V429G |
| ﻿***BARD1***  D601A | ﻿***BCOR***  H1458N | ﻿***BRCA1***  K1109N, K996T, N343S, R691I, S561Y, T676A | ﻿***BRCA2***  A2227T, E1511K, H1731N, H2417N, K1082N, K1226Q,  K1860E, K3179T, K585N,  K862Q, M3322I, N1377H,  N1448H, N1642S, S137A,  T3165N, V464M |
| ﻿***BRD4***  D389N, K317T | ﻿***BRIP1***  G212D, K703Q | ﻿***BTG1***  S91R | ﻿***BTG2***  A129T |
| ﻿***C11ORF30 (EMSY)***  R206Q | ﻿***CARD11***  D799N, E399K,  E813K, R848C | ﻿***CASP8***  I160T, R449Q,  R52I, R68Q | ﻿***CBFB***  F17L |
| ﻿***CBL***  R343Q | ﻿***CCNE1***  V107A | ﻿***CD22***  E126D, Q89H and S395F | ﻿***CD274 (PD-L1)***  Q173H, R140I |
| ﻿***CD79B***  S7Y | ﻿***CDC73***  V323A | ﻿***CDH1***  F810V and N56S | ﻿***CDK12***  E1024K, M143T,  S838L, V302G |
| ﻿***CDK6***  T70I | ﻿***CDKN1B***  F64V | ﻿***CDKN2A/B***  p15INK4b R114C | ﻿***CHEK2***  E457D, E64D,  K279T, S435P |
| ﻿***CIC***  K194Q | ﻿***CREBBP***  K389N | ﻿***CTCF***  E7K | ﻿***CTNNA1***  E110G |
| ﻿***CTNNB1***  G84R, K671T, V166L | ﻿***CYP17A1***  R125Q | ﻿***DAXX***  E438D, E68K | ﻿***DDR2***  D710Y |
| ﻿***DIS3***  D776E | ﻿***DNMT3A***  E426A, T437M | ﻿***EGFR***  F1176V, L450V | ﻿***EP300***  L2154R, R1356Q,  S2302Y, S964Y |
| ﻿***EPHA3***  E930* | ﻿***EPHB1***  I894L, R637C, R767H | ﻿***EPHB4***  R81W | ﻿***ERBB2***  N1219T |
| ﻿***ERBB3***  D1014Y, K314T | ﻿***ERCC4***  L185I, R292* | ﻿***ERG***  I163L, R287C | ﻿***ERRFI1***  P333L |
| ﻿***EZH2***  E162A, K497T, T383A | ﻿***FAM123B***  E933K, S246Y | ﻿***FANCC***  Q13P | ﻿***FGF10***  L90I |
| ﻿***FGF19***  R99W | ﻿***FGF6***  E75A | ﻿***FGFR2***  Y345D | ﻿***FLT1***  R665Q |
| ﻿***FLT3***  E293K, E916* | ﻿***FUBP1***  K446N | ﻿***GABRA6***  L424I, T282A, Y420H | ﻿***GATA4***  F384L, R362C |
| ﻿***GATA6***  F139fs*161 | ﻿***GNA13***  K121N | ﻿***GNAQ***  N222H, S268L | ﻿***GNAS***  R169Q, R601Q |
| ﻿***GRM3***  F765V | ﻿***GSK3B***  S237I | ﻿***HDAC1***  K464R | ﻿***HGF***  E351*, F593S, V200F |
| ***HSD3B1***  F218L, F286I | ***IKZF1***  F282C | ***INPP4B***  D276E | ***IRF2***  C83R |
| ***IRF4***  K55N | ***JAK2***  K607T, L545V, R234H, R938Q, S593P | ***KDM5A***  K1554I, K96T,  N372H, V405A | ***KDM5C***  F671L |
| ***KDM6A***  T1323A | ***KDR***  I784N, K270N, K286N, N622T, V1088I | ***KEAP1***  F220L | ***KEL***  T101A |
| ***KIT***  E849K, T322A | ***KMT2A (MLL)***  K1270Q | ***KRAS***  E98*, R161* | ***MAP2K4***  R304Q, T349M |
| ***MAP3K1***  M225I | ***MAP3K13***  L304F | ***MAPK1***  S266Y | ***MDM4***  D173Y |
| ***MERTK***  A258V, E240K, E243D, K953N, Q241H | ***MET***  F274C, F479L, H1106N, N285K, R1170*, S663L | ***MITF***  D442N, E422G, R355Q | ***MLH1***  L521I |
| ***MLL2***  Q3757R, R5454Q | ***MPL***  R43Q | ***MRE11A***  D498N | ***MSH***  ***2***N583H |
| ***MSH6***  K1042T, K885N | ***MST1R***  G1099R | ***MTAP***  H88P | ***MTOR***  D523N, F821C, K384N |
| ***MUTYH***  R177W | ***MYCL1***  N243S | ***MYCN***  R286C | ***MYD88***  D288N |
| ***NF1***  D287Y, I1782M, K757N, L2325R, L611I, R440Q | ***NF2***  D277G, G302R | ***NFE2L2***  R499W | ***NKX2-1***  G358D |
| ***NOTCH1***  T1602A | ***NOTCH2***  L1426R, R1718H | ***NOTCH3***  R1915H | ***NRAS***  S89* |
| ***NSD3 (WHSC1L1)***  E1332K | ***NT5C2***  N126H | ***NTRK1***  T18A | ***NTRK2***  E84K, T290K |
| ***NTRK3***  K107Q, V468A, splice site 676-1G>T | ***PALB2***  E227K, K353T, N442H, Q347H | ***PAX5***  A385T, R305H | ***PBRM1***  R202C |
| ***PDCD1LG2 (PD-L2)***  P219S | ***PDGFRA***  K702N, R376Q, S78N | ***PDGFRB***  E935D | ***PIK3C2B***  E762D |
| ***PIK3C2G***  D313Y, E60G,  splice site 679-2A>C | ***PIK3CA***  R1023*, S1015Y | ***PIK3CB***  A593V, D509N, R149* | ***PIK3R1***  I538S |
| ***PIM1***  E283D | ***POLD1***  F965, R306H | ***PPARG***  R316C | ***PTEN***  E99D, S364F |
| ***PTPN11***  D64Y | ***QKI***  S217Y | ***RAD21***  R391C | ***RAD51***  I288S |
| ***RAD51B***  I383M | ***RAD52***  S138P | ***RAD54L***  K354N | ***RB1***  F131L, I835F,  R910Q, S302Y |
| ***REL***  N605H, R45Q | ***ROS1***  A1103T, D791Y, E1244*, E1589*, E1598*, E402*, H1701R, I929M, K1820T, R1310,  R360I, V1251A, V614L | ***SETD2***  F40L, H216P, K650E, N811T | ***SF3B1***  E776*, K252N, R135Q, R318Q, R495S, R957Q and rearrangement |
| ***SGK1***  K137N, S145L | ***SMAD2***  E185K | ***SMARCA4***  R397Q | ***SMARCB1***  L319R |
| ***SNCAIP***  Y881* | ***SOCS1***  E189D | ***SPEN***  D453Y, F3634L, G42E, R1917H, R2327W, R795Q, T922A, Y3573H | ***STAG2***  E534G, I144L |
| ***STK11***  D355N | ***SUFU***  Y90C | ***TBX3***  E275K | ***TEK***  *1125Yext*32, D455A, F404L, F754S, S658F |
| ***TET2***  S153Y | ***TIPARP***  F626L | ***TNFAIP3***  N414K | ***TSC1***  R484K |
| ***TYRO3***  N626T, S845C | ***VHL***  P154S | ***WHSC1 (MMSET)***  E185K, R526I | ***XPO1***  *E1037D, F927V, G128R* |

**Supplementary Table 3C:** Variants of unknown significance detected in patient # 3’s liquid biopsy sample after completing systemic treatment.

| ﻿***﻿DIS3***  ﻿D776E | ***﻿FGF19***  G73V | ***MYCN***  ***﻿***S380fs*4 | ***PAX5***  ***﻿***R305H |
| --- | --- | --- | --- |
